# Parallelism in eco-morphology and gene expression despite variable evolutionary and genomic backgrounds in a Holarctic fish

**DOI:** 10.1371/journal.pgen.1008658

**Published:** 2020-04-17

**Authors:** Arne Jacobs, Madeleine Carruthers, Andrey Yurchenko, Natalia V. Gordeeva, Sergey S. Alekseyev, Oliver Hooker, Jong S. Leong, David R. Minkley, Eric B. Rondeau, Ben F. Koop, Colin E. Adams, Kathryn R. Elmer

**Affiliations:** 1 Institute of Biodiversity, Animal Health and Comparative Medicine, University of Glasgow, Glasgow, United Kingdom; 2 Vavilov Institute of General Genetics, Russian Academy of Sciences, Moscow, Russia; 3 Koltzov Institute of Developmental Biology, Russian Academy of Sciences, Moscow, Russia; 4 Severtsov Institute of Ecology and Evolution, Russian Academy of Sciences, Moscow, Russia; 5 Scottish Centre for Ecology and the Natural Environment, University of Glasgow, Rowardennan, Loch Lomond, Glasgow, United Kingdom; 6 Biology/Centre for Biomedical Research, University of Victoria, British Columbia, Canada; University of Wyoming, UNITED STATES

## Abstract

Understanding the extent to which ecological divergence is repeatable is essential for predicting responses of biodiversity to environmental change. Here we test the predictability of evolution, from genotype to phenotype, by studying parallel evolution in a salmonid fish, Arctic charr (*Salvelinus alpinus*), across eleven replicate sympatric ecotype pairs (benthivorous-planktivorous and planktivorous-piscivorous) and two evolutionary lineages. We found considerable variability in eco-morphological divergence, with several traits related to foraging (eye diameter, pectoral fin length) being highly parallel even across lineages. This suggests repeated and predictable adaptation to environment. Consistent with ancestral genetic variation, hundreds of loci were associated with ecotype divergence within lineages of which eight were shared across lineages. This shared genetic variation was maintained despite variation in evolutionary histories, ranging from postglacial divergence in sympatry (ca. 10-15kya) to pre-glacial divergence (ca. 20-40kya) with postglacial secondary contact. Transcriptome-wide gene expression (44,102 genes) was highly parallel across replicates, involved biological processes characteristic of ecotype morphology and physiology, and revealed parallelism at the level of regulatory networks. This expression divergence was not only plastic but in part genetically controlled by parallel *cis*-eQTL. Lastly, we found that the magnitude of phenotypic divergence was largely correlated with the genetic differentiation and gene expression divergence. In contrast, the direction of phenotypic change was mostly determined by the interplay of adaptive genetic variation, gene expression, and ecosystem size. Ecosystem size further explained variation in putatively adaptive, ecotype-associated genomic patterns within and across lineages, highlighting the role of environmental variation and stochasticity in parallel evolution. Together, our findings demonstrate the parallel evolution of eco-morphology and gene expression within and across evolutionary lineages, which is controlled by the interplay of environmental stochasticity and evolutionary contingencies, largely overcoming variable evolutionary histories and genomic backgrounds.

## Author summary

A renowned natural model system for adaptive evolution is the repeated and rapid divergence of fishes into different sympatric trophic and morphological specialists, known as ecotypes. The drivers and constraints of these repeated divergences are complex and not well understood but it is often observed that postglacial fishes diverge in predictable patterns. Here we use a framework of parallel (or convergent) evolution to test the predictability of divergence in the most variable northern freshwater fish, the Arctic charr. Using a hierarchy of replication—from individuals to divergent phylogeographic lineages—we detect parallel evolution of foraging-related traits despite variation in genomic backgrounds and evolutionary histories. The level of phenotypic parallelism can be explained by the complex interplay of environment, shared genetic variation, and variability in gene expression. While phenotypic divergence may be determined and/or constraint by genetic and molecular divergence, the direction of change is largely determined by molecular parallelism and shared adaptive genetic variation. These are in turn associated with environmental similarity, reflected as ecosystem size. We suggest that gene expression facilitates parallel ecotype evolution, but that the extent of parallelism is further influenced by the level of shared genetic variation and ecological opportunity.

## Introduction

The degree to which the pathways of evolution are predictable, particularly under complex natural conditions, remains one of the greatest questions in evolutionary biology [1,2]. Numerous species in nature have repeatedly evolved similar phenotypes in response to similar environmental challenges, strongly suggesting repeatability and predictability of evolutionary trajectories [3–7] and highlighting the pervasive role of natural selection in evolution [8,9]. Despite this, extensive variation in the magnitude and direction of evolutionary trajectories has been observed in some classic examples of ‘parallel evolution’ [7,10–12]. Stochastic factors such as differences in the local environment, gene flow, and selection regimes, or contingencies such as genomic background, demographic history, and the genetic architecture of adaptive traits, can lead to departures from phenotypic parallelism and non-parallelism at the genomic level [6,7,13]. Despite a growing number of studies into the parallelism or convergence of evolutionary trajectories, our ability to predict evolutionary outcomes is still limited [14]. To improve predictability, it is critical to understand the evolutionary routes leading to replicated ecological divergence in a range of independent systems [15] and to disentangle the impact of various contingent and stochastic factors on parallel evolution.

Evolutionary predictability is difficult to quantify in a biologically meaningful way when selection is multifarious and traits are quantitative [10,14]. Multiple statistical frameworks have been developed recently to address parallel evolution in natural systems, as a proxy for predictability of evolution, consistency of selection gradients, and pervasiveness of deterministic processes [16,17]. Parallel evolution of phenotypes can be inferred from specific traits, overall morphology, and/or ecological niche [7,12,18,19] and can be quantified as replicated evolutionary trajectories [16] or as the amount of variation explained by ecotype [17]. Parallel evolution at the genetic level is evidenced by shared genes and genetic regions underpinning parallel phenotypes across replicates, either due to repeated *de novo* mutation, recruitment of shared standing genetic variation, or introgression [3,13]. Furthermore, parallel evolution can potentially occur through convergent functional genomic changes despite non-parallel genetic changes, e.g. through differential expression of the same genes or genes in the same pathway [3,20–23]. Here, we describe parallel evolution as the replicated, independent evolution of quantitatively similar adaptive phenotypes (ecotypes) by similar genomic underpinnings [3]. The study of replicated natural systems at different organismal levels is critical to identifying the genetic, environmental, and selective components underlying parallel evolution and its deviations but to date has been underexplored [11,24].

The replicated ecological and morphological post-glacial diversification of fishes into distinct trophic specialists in freshwater lakes is a powerful natural experiment for testing evolutionary predictability [25–27]. Divergence in such fishes typically occurs due to influences of disruptive selection and often involves the independent evolution of similar ecotypes [10,28–32]. In the Holarctic salmonid species Arctic charr (*Salvelinus alpinus*), sympatric ecotype pairs that differ in several heritable phenotypic traits, such as body shape, body size, trophically relevant morphology, and life history, are abundantly replicated [26,27,33–35]. Ecotypes distinguished by diet and foraging tactics include: Planktivores and Piscivores which feed in the pelagic, and Benthivores which feed in the benthic-profundal or benthic-littoral zone [26,27,33,34]. These ecotypes can be found across the distribution of Arctic charr [26,27] and most abundantly in the Atlantic and Siberian lineages, which likely diverged before the last glacial maximum during the Pleistocene [36]. Arctic charr ecotypes likely evolved following the last glacial maximum (around 10,000–20,000 years ago), after the colonization of newly formed postglacial lakes by putatively pelagic charr from different glacial refugia populations. However, the phenotypic parallelism, evolutionary histories, and adaptive genomic responses of ecotypes across lakes and lineages has never been investigated.

**Fig 1:**
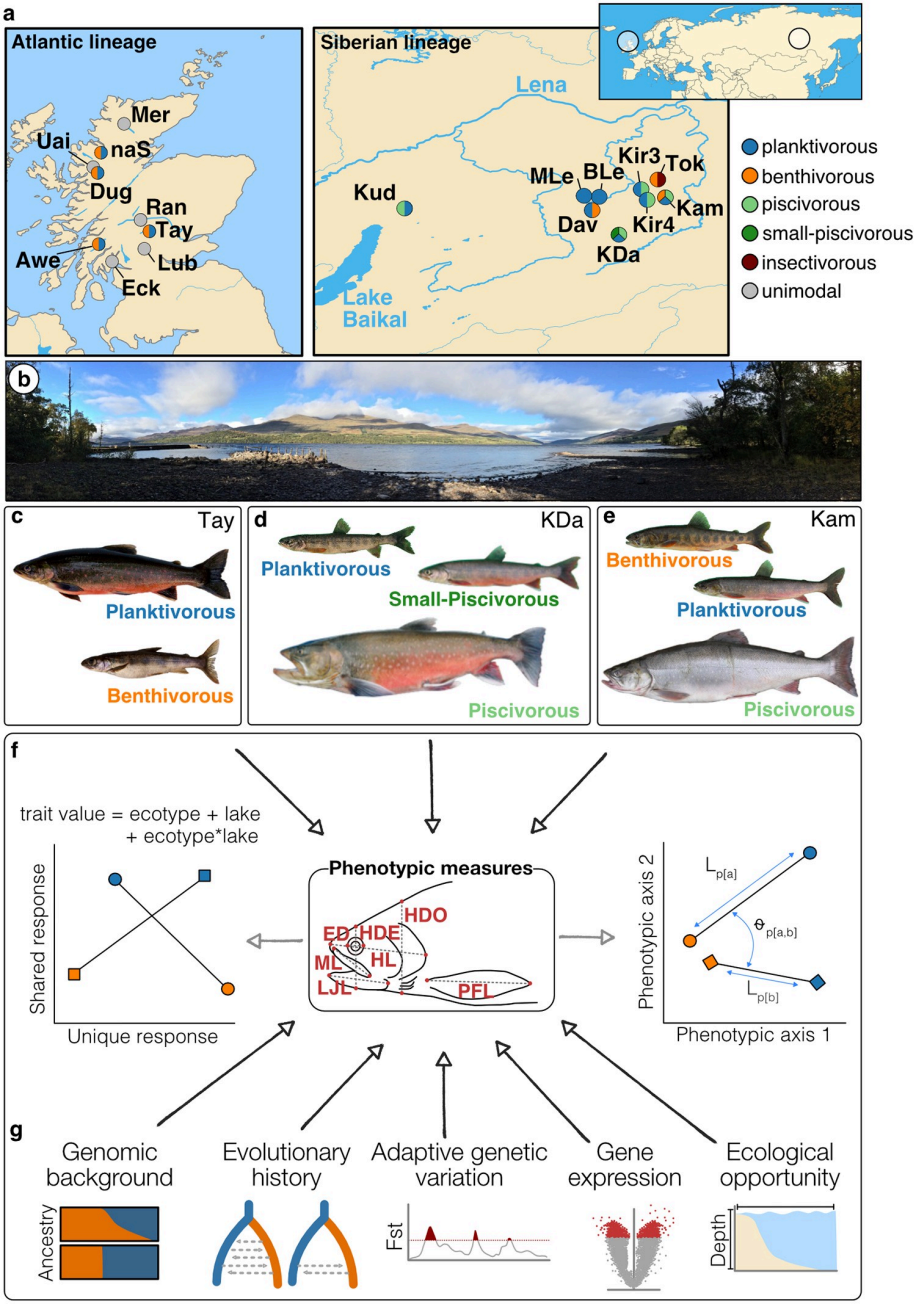
Sampling locations, ecotypes and study design. (**A**) Maps showing the sampling locations of Arctic charr from the Atlantic lineage in Scotland (N = 440) and the Siberian lineage in Transbaikalia, Russia (N = 1,009). The colour combination of each dot shows the ecotypes sampled from each location. Full names of lakes are given in S1 Table. (**B**) Picture showing the sampling site at Loch Tay in Scotland. (**C**–**E**) Representative individuals of the (**C**) two sympatric ecotypes from Loch Tay in Scotland and the three sympatric ecotypes from (**D**) Kalarskii Davatchan (KDa) and (**E**) Kamkanda (Kam) in Transbaikalia. Individuals not to scale. (**F-G**) The study design is centred around testing the (**F**) extent of phenotypic parallelism between replicated ecotypes within and across two evolutionary lineages using phenotypic trajectory analyses (illustrated on the right) and variance partitioning with linear models (illustrated on the left) based on 7 linear measurements (shown in the middle). Linear traits measured: HDE–head depth at eye, HDO–head depth at operculum, HL–head length, PFL–pectoral fin length, ED–eye diameter, ML–maxilla length, LJL–lower jaw length. Fork length (FL) was also measured. (**G**) In addition, we analysed the extent of parallelism from the genomic background to gene expression and assessed the impact of variation on these different organisational levels on the extent of phenotypic parallelism.

Here we tested the extent of parallelism in phenotype, evolutionary history, genomic patterns, and gene expression in repeated divergences of Arctic charr in their environmental context from eleven lakes within and across two distinct evolutionary and geographic lineages (Atlantic and Siberian) (Fig 1). To do so we contextualised the contemporary population genomic variation by resolving the historical evolution and demography. We propose that parallel evolution of ecotypes will be evident in significant similarity of phenotypes and significant sharing of putatively adaptively relevant genomic regions [3,10,13–15,18]. Finally, to explicitly test the power to predict evolution in this system, we investigated how variation at different intrinsic organisational levels, such as genomic variation or gene expression, and extrinsic factors such as ecosystem size, correlate with phenotypic parallelism. Overall, we show that parallel adaptive phenotypes evolved repeatedly and independently within and across lineages despite differing evolutionary histories. Our findings suggest that the extent of parallel evolution is shaped by the interplay of genomic, transcriptomic and environmental variation.

## Results

### Phenotypic divergence and parallelism

To test for parallelism in ecologically relevant phenotypes, we assessed morphology based on seven linear traits measurements of Arctic charr from the Atlantic lineage (Scotland) and Siberian lineage (Transbaikalia) (Fig 1F; total N = 1,329 individuals). This included eleven replicate ecotype pairs: six benthivorous-planktivorous and five piscivorous-planktivorous combinations (Fig 1A–1E; S1 Table). Additionally, three populations were included as outgroup comparisons in morphological analyses (piscivorous ecotype from Kudushkit, planktivorous ecotypes from Maloe and Bol’shoe Leprindo) due to the absence of phenotypic information for sympatric sister ecotypes, and one population (Tokko) with a benthivorous and mainly insectivorous ecotype that has no population replicate. These populations were excluded from the statistical parallelism analysis but included in genetic analyses.

**Fig 2:**
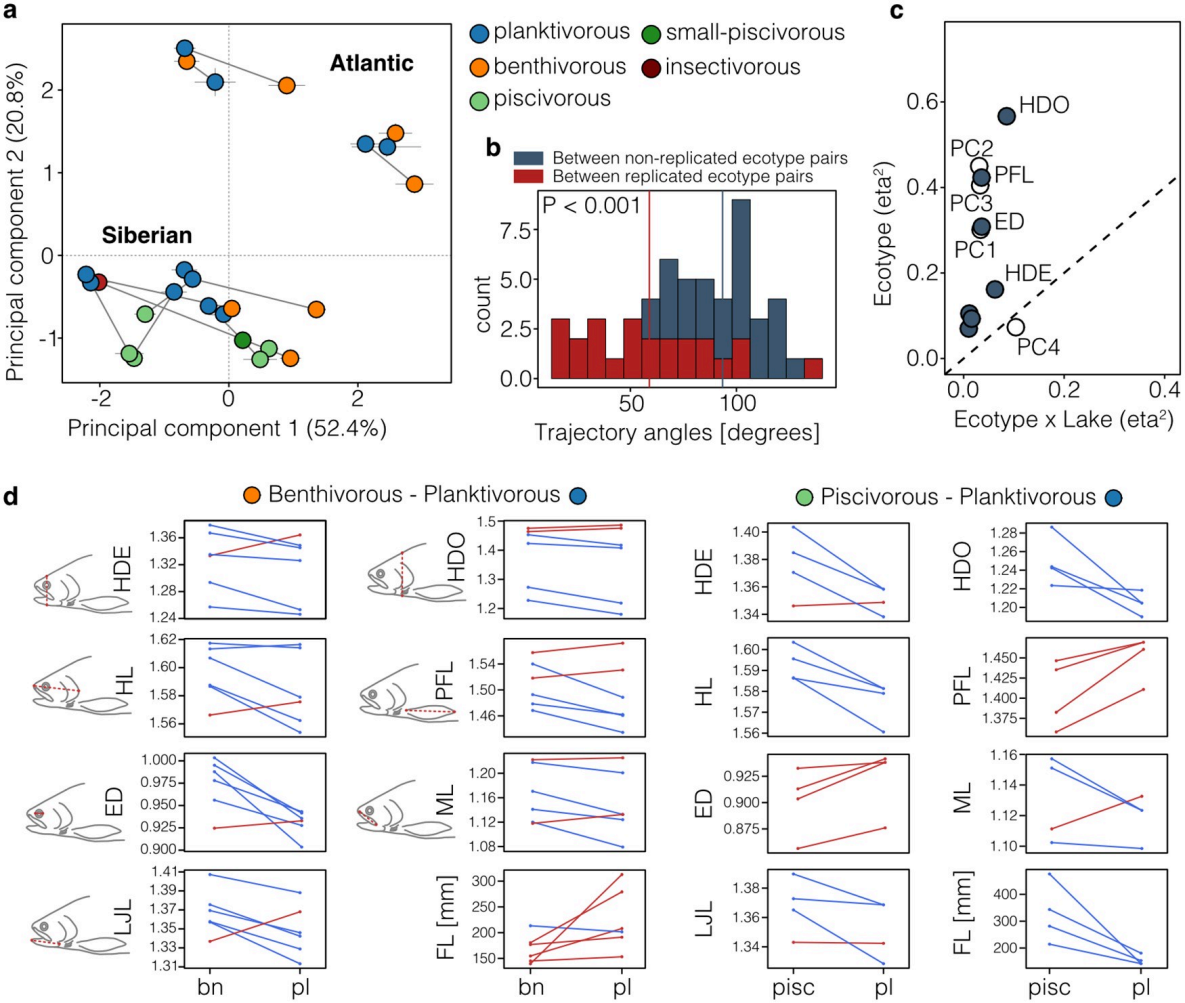
Continuum of phenotypic parallelism. (**A**) Principal components plot based on all seven linear traits showing the centroid ± s.e. for each ecotype (N = 1,329 individuals), with centroids of sympatric ecotypes connected by trajectories. Points are coloured by ecotype: bn–benthivorous, pl–planktivorous, pisc–piscivorous, pisc-s–small-piscivorous, insct–insectivorous. (**B**) Phenotypic trajectory angles between replicated ecotype pairs and between non-replicated ecotype pairs, with mean angles highlighted by dashed lines. Angles between replicated ecotype pairs were significantly smaller compared to non-replicated ecotype pairs (Wilcoxon test: P < 0.001) (**C**) Effect sizes (partial η^2^) of the ecotype and ‘ecotype x lake’ interaction terms for all seven linear traits (dark dots) and PC1 to PC4 (white dots). Traits above the dashed diagonal line show stronger parallel than non-parallel divergence across ecotype pairs. (**D**) Mean size-adjusted trait-values and mean fork length (in mm) are plotted for each benthivorous-planktivorous and piscivorous-planktivorous ecotype pair, with means for sympatric pair being connected by a line. These reaction norms are colour coded blue and red highlighting the decrease or increase of trait values, respectively, between benthivorous (bn) or piscivorous (pisc) and planktivorous (pl) ecotypes. The measured traits are illustrated next to each plot for the benthivorous-planktivorous pairs. See text, panel 1C and S1 Fig for details on statistical results.

Principal component analyses separated sympatric ecotypes along PC1 (52.4%) and PC3 (10.1%), while the two evolutionary lineages separated along PC2 (20.8%) (Fig 2A, S1B Fig). This suggests strong phenotypic parallelism in ecotype divergence within and across evolutionary lineages.

To quantify the direction and magnitude (length and direction of trajectories) of the phenotypic divergences across replicate sympatric ecotypes, we used a phenotypic trajectory approach on all traits combined [7,37,38]. The length of these trajectories (L) describes the magnitude of divergence and the angle (*θ*) between trajectories describes their direction through multi-trait space (see [37] for details; Fig 1F). Thus, the difference in phenotypic trajectory length (ΔL_P_) and the direction of phenotypic trajectories (*θ*_P_) define the extent of multivariate phenotypic parallelism; completely parallel ecotype pairs are those diverged to the same extent (ΔL_P_ not different from zero) and in the same direction (*θ*_P_ angle not different from zero) [12,37].

Using this approach, we found that the direction of phenotypic change between ecotype pairs was highly variable across replicates (Fig 2B, S1B Fig; mean *θ*_P_ = 58.95° ± 31.54 s.d. (standard deviations)) though several ecotype pairs were significantly parallel (P > 0.05, S2 Table). Angles ranged from highly parallel (e.g. *θ*_P_ = 14.2°, P = 0.27) to antiparallel (e.g. *θ*_P_ = 133.5°, P = 0.001) (S2 Table). In general, angles were 34.5° smaller (i.e. trajectories more similar) between replicated ecotype pairs than across the different ecotype pairs (i.e. benthivorous-planktivorous pairs vs. piscivorous-planktivorous pairs; mean *θ*_P_ = 93.48° ± 18.94 s.d.) (Wilcoxon rank sum test: P<0.001) (Fig 2B).

Compared to the direction of change, the magnitude of divergence between sympatric ecotype pairs was more similar across replicate pairs (mean ΔL_P_ = 0.033 ± 0.021 s.d.). In general, Siberian ecotype-pairs, particularly the piscivorous-planktivorous ecotypes, were more parallel (mean *θ*_P_ = 46.6° ± 21.3 s.d., mean ΔL_P_ = 0.035 ± 0.020 s.d.) than ecotype-pairs from the Atlantic lineage (*θ*_P_ = 69.4° ± 35.6 s.d., mean ΔL_P_ = 0.036 ± 0.021 s.d.; S2 Table). Pronounced parallelism was also found across the lineages; for example, the benthivorous-planktivorous ecotype pairs from Dughaill and Kamkanda were highly parallel in both direction and magnitude of divergence (*θ*_P_ = 24.4°, P = 0.07; ΔL _P_ = 0.0152, P = 0.244).

Second, to deconstruct the extent of parallelism in specific traits related to trophic morphology, habitat use, and swimming ability (Fig 1F), we used a trait-by-trait linear modelling approach across all ecotype pairs. We found that the parallel divergence elements of the model (the ecotype term) explained more phenotypic variance (partial-eta-squared: η^2^) than the non-parallel elements of divergence (‘ecotype x lake’ and ‘ecotype x lineage’ interaction terms) for all traits, ranging from η^2^_eco_ = 0.07 for HL to η^2^_eco_ = 0.57 for HDO (Fig 2C,. S1A Fig). However, as indicated by the significant non-parallel interaction terms, trait differences between ecotypes varied across populations, ranging from highly parallel to antiparallel in some populations and traits (Fig 2D, S3 Table). In combination, these results suggest that although the absolute trait values differ in each population or lineage the divergence between ecotypes is largely predictable across lakes and lineages.

Three traits in particular were significantly parallel: eye diameter, pectoral fin length, and head depth. For eye diameter (ED; η^2^_eco_ = 0.31) and pectoral fin length (PFL; η^2^_eco_ = 0.42), ecotype explained the highest proportion of phenotypic variation (Fig 2C and 2D, S1 Fig, S3 Table). This indicates that across lakes and also across evolutionary lineages, ecotypes are consistently diverged in those traits, which are closely associated with habitat use (ED) and swimming performance (PFL) [39]. Furthermore, head depth at the operculum (HDO) had a larger amount of variation explained by ecotype (η^2^_eco_ = 0.57) compared to the lake effect (η^2^_lake_ = 0.09), suggesting that this trait is under strong natural selection. The consistent variation in head depth across lineages (η^2^_lineage_ = 0.92) could potentially be explained by the presence of deep-headed piscivorous ecotypes in the Siberian lineages.

Overall, these results indicate that ecologically replicated Arctic charr ecotypes show phenotypic parallelism, in both direction and magnitude of divergence, although at variable levels between populations and morphological traits.

### Contemporary population genetic structure

**Fig 3:**
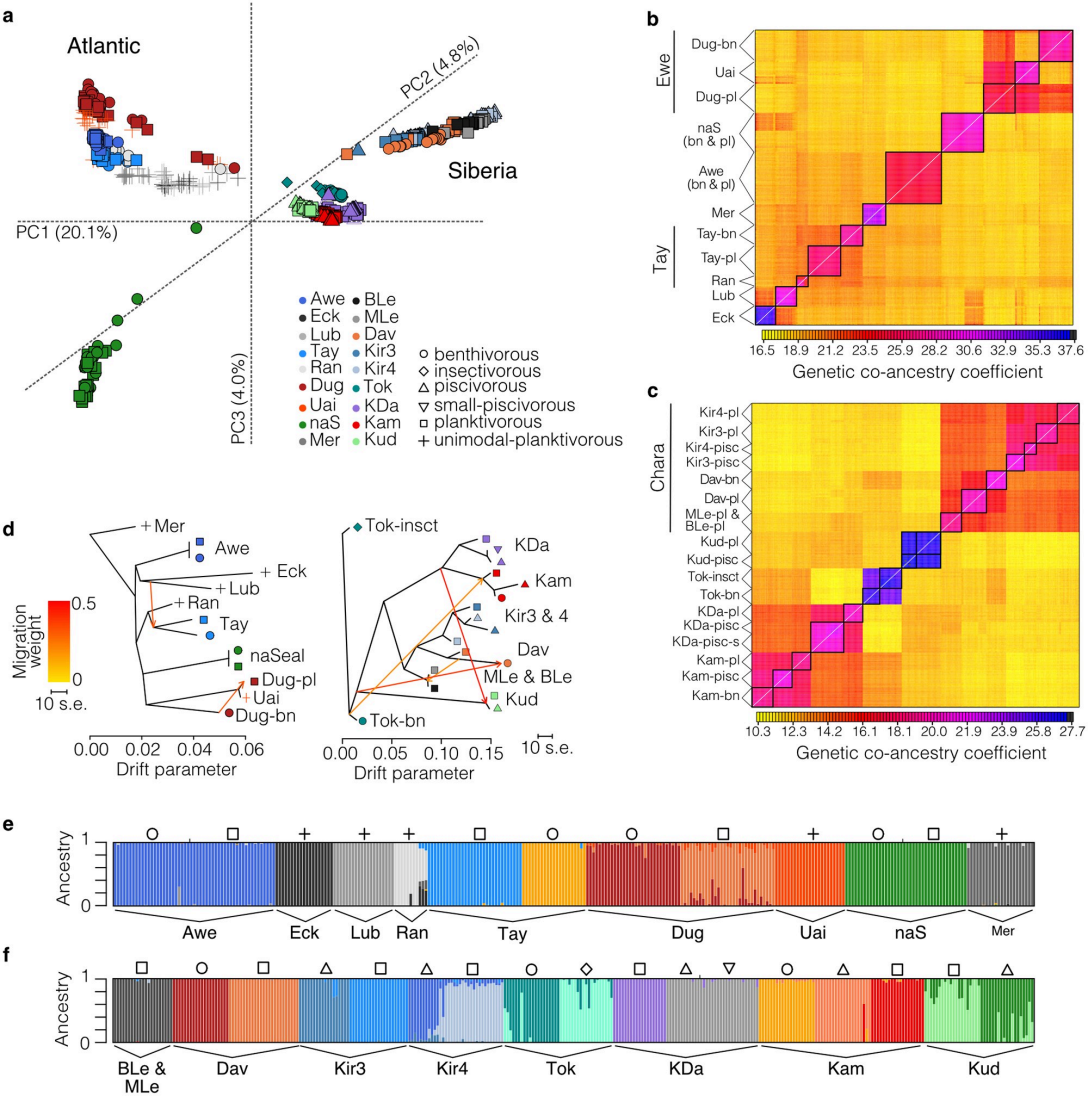
Hierarchical population genetic structure and genomic background divergence. (**A**) 3-dimensional principal components plot for all individuals (N = 630) based on 12,215 SNPs. Lake abbreviations are explained in S1 Table. PC1 mostly separates the Atlantic and Siberian lineage, PC2 mostly separates catchments within the Siberian lineage and PC3 separates na Sealga from all other populations within the Atlantic lineage (**B,C**) fineRADstructure results showing co-ancestry coefficients between individuals from the (**B**) Atlantic and (**C**) Siberian lineage. Ecotypes or lake populations that form discrete genetic clusters are enclosed in black boxes. Note the high genetic co-ancestry across lakes within the same catchment, such as (**B**) Dughaill (Dug) and Uaine (Uai) in the Ewe catchment or (**C**) Kiryalta-3 (Kir3), Kiryalta-4 (Kir4), Davatchan (Dav), and Maloe and Bol’shoe Leprindo (MLe, BLe) in the Chara catchment. Ecotype descriptions (abbreviations in S1 Table) are shown unless populations were unimodal or ecotype-associations unknown. Populations are ordered in the same way along the x-axis of the co-ancestry matrices. (**D**) Allele frequency-based maximum-likelihood trees showing the most likely phylogenetic relationships across lakes and ecotype pairs by lineage, including two and four fitted migration events in the Atlantic and Siberian lineage, respectively. Migration events are shown as arrows coloured by migration weight. (**E,F**) *Admixture* plots showing the genetic ancestry for all individuals from the (**E**) Atlantic and (**F**) Siberian lineage for K = 11 and K = 16, respectively. Ecotypes are marked by symbols above each cluster.

To test if replicated ecotypes most likely evolved in parallel independently, we analysed population genetic co-ancestry, introgression, and genetic differentiation within and across evolutionary lineages. Based on a global SNP dataset of all individuals (N = 12,215 SNPs from ddRADseq; N = 630 individuals), we found that principal components clearly distinguished the two evolutionary lineages, clustered individuals by lake of origin, and further clustered lakes by broader river catchment [40,41] (Fig 3A; S2 Fig). This structure is also supported by haplotype-based genetic co-ancestry analyses (Fig 3B and 3C, S3 Fig, S1 Table). Histories of independent colonization were generally also supported by near complete mitochondrial haplotype sharing of individuals within lakes, and in some cases across nearby lakes (S4 Fig) [42].

However, the hierarchical population genetic clustering deviated on some occasions. We found elevated co-ancestry between geographically distinct populations (e.g. Kamkanda and Tokko in Fig 3C). In two cases, sympatric ecotype pairs formed polyphyletic genetic clusters (Fig 3D; S5 Fig), with one ecotype being genetically more similar to the ecotype from a neighbouring lake than to its sympatric pair (piscivorous ecotypes in Kiryalta-3 and Kiryalta-4, planktivores from Dughaill and Uaine; Fig 3B–3E; S2–S5 Figs). These instances suggest introgression and non-independent divergences within and across lakes.

Therefore, we used *f-statistics* and *D-statistics* to test the role of introgression in repeated ecotype divergence in more detail (see S1 Text for detailed explanation; S4 Table and S5 Table; S5 and S6 Figs). These analyses showed that although signals of introgression were detected across many populations (S6 Fig), introgression was mostly not specific to replicated ecotypes from different lakes but rather detected across different ecotypes within and across lakes.

Admixture analyses further suggested that sympatric ecotype pairs differed widely in their degree of genetic admixture (Fig 3D–3F). The distribution of genome-wide genetic differentiation was also highly variable across populations (mean Fst ranging from 0.011 to 0.329, S6 Table), with differentiation between sympatric ecotypes in some cases being higher than between allopatric comparisons (S7 Fig). In two populations in the Atlantic lineage, Awe and na Sealga, no significant genome-wide genetic differentiation could be detected between sympatric ecotypes (Fig 3E). Overall these results suggest that most ecotype pairs have diverged independently and that the contemporary population genetic structure is highly variable across replicates.

### Evolutionary histories of ecotype divergence

**Fig 4:**
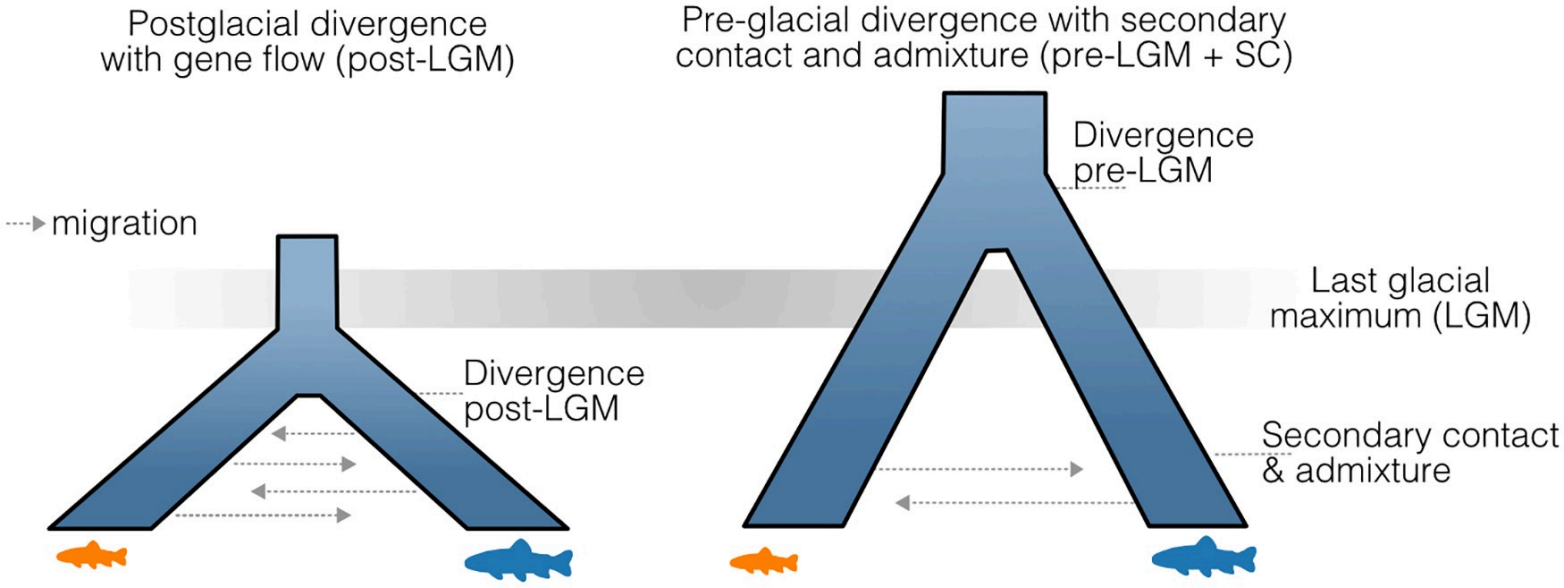
Evolutionary histories of sympatric ecotype pairs. Illustrations of the two categorical classifications of evolutionary histories based on cumulative evidence from demographic models and genetic ancestry analyses.

To test the association of evolutionary history and demographic parameters with ecotype parallel evolution [6,7,13], we used coalescence simulations implemented in *fastsimcoal2* [43] to model the history and demography for each sympatric ecotype pair (two-population and three-population models, S8 Fig). We found that 11 out of 13 ecotype pairs likely evolved following postglacial secondary contact and admixture of ancestral populations that had diverged prior to the last glacial maximum (LGM) (Fig 4; S9 Fig, S7 Table). Variations of the secondary contact (SC) model had the best fit in most Siberian populations. Isolation-with-migration and introgression (IMint) models had the strongest support in two Atlantic lineage populations, Dughaill and Tay. Combined evidence from inferred divergence times (T_DIV,Dug_ = 16690 generations/50070 years, T_DIV,Tay_ = 11461 generations/34383 years), timing of introgression (T_Int,Dug_ = 67 generations/201 years, T_Int,Tay_ = 1266 generations/3798 years), and changes in the rate of gene flow over time (S9 Fig) suggest a pre-glacial divergence with postglacial admixture for Dughaill and Tay, effectively equivalent to a secondary contact model with weak gene flow between glacial lineages during the last glaciation. In two ecotype pairs from Scotland, Awe and na Sealga, we found evidence for divergence-with-gene-flow of ecotypes after the last glaciation (isolation-with-migration model; T_DIV,Awe_ = 1245 generations/3735 years, T_DIV,naS_ = 4795 generations/14385 years) (Fig 4A, S9 Fig), consistent with sympatric speciation [44]. It should be noted that competing models could not be excluded in all populations, such as in Kamkanda (S7 Table), so higher density genomic data will be needed to better resolve these complex evolutionary histories.

The demographic parameters inferred from the most likely model for each population varied considerably in the initial divergence times between ancestral populations (910–16,690 generations ago) and the timing of secondary contact (56–3,783 generations ago) (S9 Fig). The extent of admixture between ancestral populations upon secondary contact also varied widely across lakes, with genome-wide proportions from 0.003–1.0 (S9 Fig). Overall these findings suggest considerable variation in evolutionary history across the different population replicates.

### Shared and unique signatures of genetic differentiation between sympatric ecotypes

To determine if parallel ecotypes evolved with similar genomic bases, we examined the extent of Fst outlier sharing, jointly examined parallel signals of selection across replicated ecotype pairs, and identified putatively adaptive loci within and across lineages.

**Fig 5:**
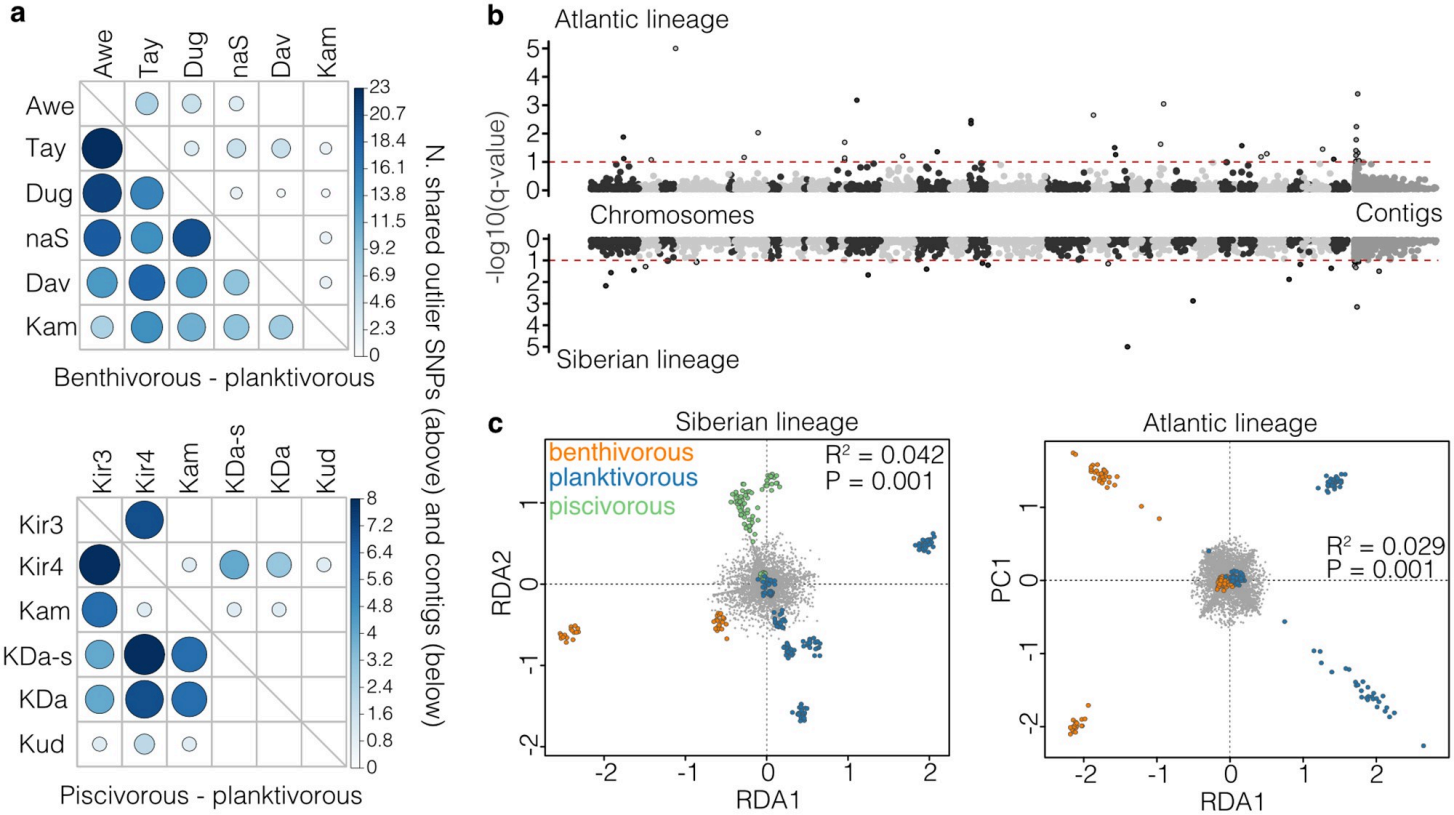
Non-parallelism in genetic differentiation and ecotype-association. (**A**) Sharing of outlier SNPs and contigs containing outlier SNPs across replicated benthivorous-planktivorous and piscivorous-planktivorous ecotype pairs. The colour and size of the dots illustrates the number of shared SNPs or contigs. None of the pairwise comparisons are significant based on permutation results (see Methods). (**B**) Manhattan plots showing the results of the hierarchical bayescan analysis. SNPs with -log10(q-values) above 1 (FDR < 0.1) show significant signs of parallel selection across ecotype pairs by lineage. The upper plot shows the results for benthivorous-planktivorous ecotype pairs in the Atlantic lineage, and the lower plot shows the results for piscivorous-planktivorous ecotype pairs in the Siberian lineage. Results for the benthivorous-planktivorous ecotype pairs from the Siberian lineage are shown in S10 Fig. Unplaced contigs are placed at the right end of the Manhattan plot. (**C**) Results of the redundancy analysis (RDA) for the Atlantic and Siberian lineages, showing varying levels of separation between ecotypes, coded by colour. The RDA significantly separates ecotypes after correcting for the effect of lake (results of ANOVA shown in plot). Grey dots show the loadings for individual SNPs. RDA1 is plotted against PC1 in the Atlantic lineage, as no second RDA is present based on the bimodal nature of the comparison. RDA1 also separated benthivorous and planktivorous ecotypes across lakes in the Siberian lineage, and RDA2 separated the piscivorous ecotype from both other ecotypes.

We found that ecotype pairs did not share more outlier loci (top 5%-Fst outlier loci; S10A–S10C Fig, S6 Table), or contigs containing outlier loci, than expected by chance in pairwise comparisons (Fig 5A). Sharing was higher when outlier SNPs were inferred based on a permutated null distribution rather than the empirical Fst distribution (S11 Fig). This is a more liberal test, but the outcome suggests that outlier SNPs in one ecotype pair often show increased differentiation in another pair, potentially through hitchhiking with a shared causal SNP [45]. However the genome-wide density of ddRADseq-based SNPs is 2.9 ± 2.7 SNPs/Mb (mean ± s.d.) across populations and the decay of linkage disequilibrium (LD) between Fst outlier SNPs and non-outlier SNPs to background levels is less than <500 kb in most populations (S12 Fig). This decay is less than the average distance between Fst outlier SNPs and neighbouring SNPs (678,248 ± 165,075 kb) but larger than the average contig length (89kb ± 361kb), so these Fst outlier approaches likely underestimate genomic parallelism.

To maximise our power to detect shared outlier loci with this population genomic dataset, we used two analytical approaches that jointly test for convergent signals of divergent selection and shared patterns of allele frequency differentiation. First, using a hierarchical implementation of bayescan, we detected signatures of parallel selection across the benthivorous-planktivorous ecotype pairs in the Atlantic lineage (33 SNPs, FDR < 0.1) and piscivorous-planktivorous populations in the Siberian lineage (26 SNPs, FDR < 0.1), but not in benthivorous-planktivorous from the Siberian lineage (Fig 5B, S10D Fig). None of the SNPs showed signs of parallel divergent selection in both lineages.

Second, using redundancy analyses, we identified 217 and 303 SNPs showing ecotype-associated allele frequency differences (z-score > 2) across lakes within the Atlantic and Siberian lineages, respectively (Fig 5C; S13 Fig). These ecotype-associated SNPs explained 2.9% (benthivorous-planktivorous in Atlantic lineage) and 4.2% (benthivorous-planktivorous-piscivorous in the Siberian lineage) of the overall genetic variation between ecotypes within each lineage. Of these, eight SNPs from independent genomic regions on seven different chromosomes (S8 Table) were shared across lineages, which is more than expected by chance (χ^2^-square; P<0.001) and suggests some genomic parallelism across lineages. In the Siberian lineage, two SNPs were detected to be under both parallel divergent selection (bayescan) and also associated with ecotype divergence (RDA).

### Parallel divergence in gene expression

To test if regulatory variation would show functional parallelism across ecotype pairs due to integrated effects of plasticity and genetically-mediated expression, we analysed genome-wide gene expression in white muscle between ecotypes from a subset of five lakes (N = 44 individuals, 30,849 genes; S1 Table). Across these, we compared differential expression, co-expression modules, and biological pathways.

**Fig 6:**
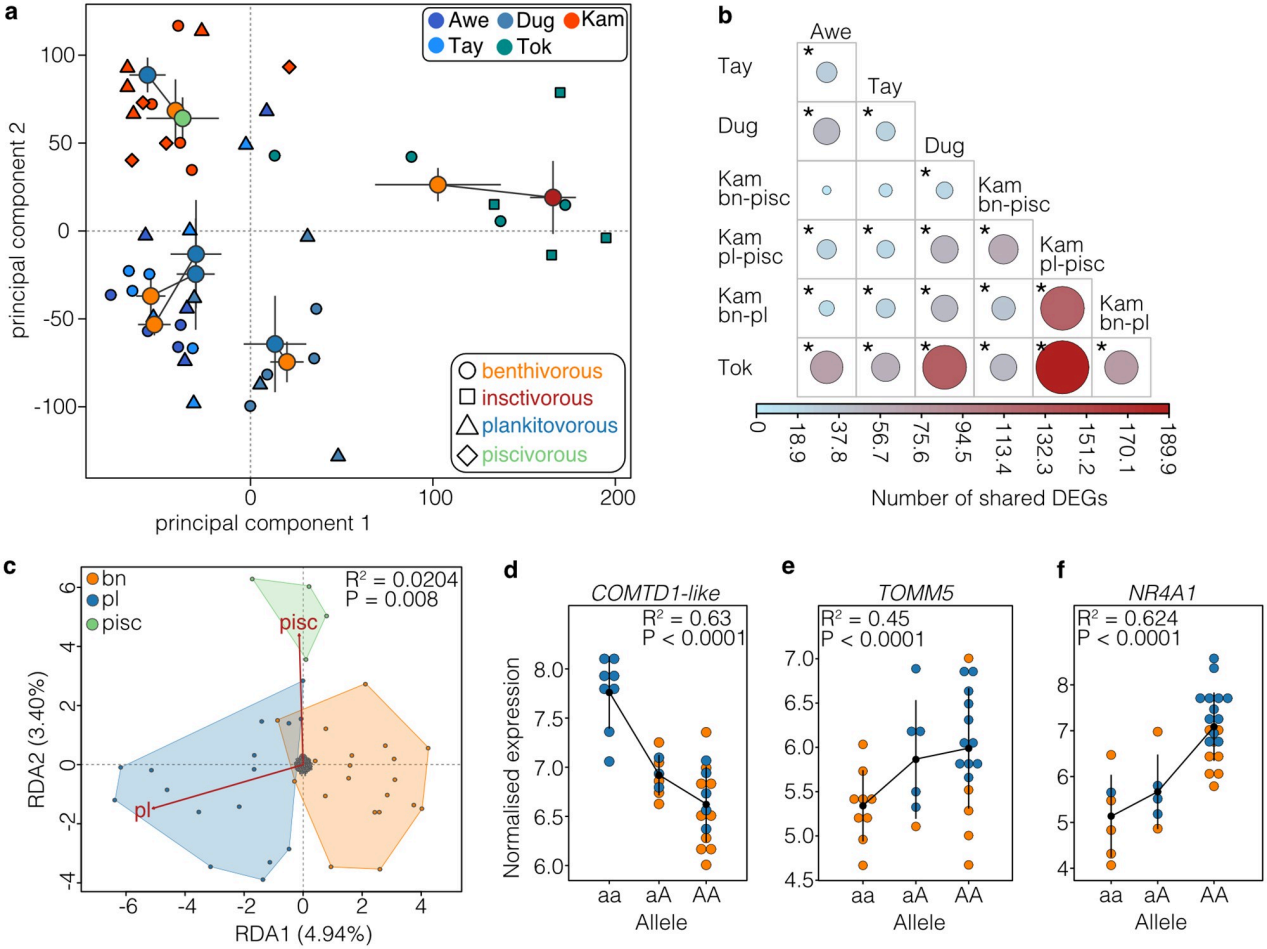
Parallelism and divergence in gene expression. (**A**) Principal components plot based on r-log transformed gene expression data (N = 30,849 transcripts). Individuals (N = 44) are shown by individual points shaped by ecotype and coloured by lake of origin. Centroids ± 1 standard error are shown for each ecotype and coloured by ecotype (blue–planktivorous, orange–benthivorous, green–piscivorous, red–insectivorous). Centroids of sympatric ecotypes are connected by a line. (**B**) Sharing of differentially expressed genes with the extent of sharing weighted by colour and circle size. Significant comparisons are highlighted with an asterisk. Ecotype abbreviations: bn–benthivorous, pl–planktivorous, pisc–piscivorous. (**C**) Biplot of the gene expression-based redundancy analysis showing significant separation of ecotypes after correction for lake and lineage, with benthivorous (orange area) and planktivorous (blue area) separating along RDA1 (4.94%) and the piscivorous (green area) splitting off along RDA2 (3.40%). (**D-F**) Examples of *cis*-eQTL in (**D**) COMTD1-like, (**E**) *TOMM5* and (**F**) *NR4A1*, showing how the expression of these ecotype-associated genes differs with genotype across individuals (points) and ecotypes. The black dot and range show the mean expression per allele ± 1 standard deviation. The R^2^ value shows the strength of correlation between allele and normalised expression.

Similar to population genetic patterns, we found a continuum of divergence in gene expression across ecotype pairs (Fig 6A; S14A Fig). Contrary to the genomic analysis, gene expression patterns were highly similar across lakes and ecotype pairs, with ecotype explaining most of the expression variation along PC1 (Fig 6A; η^2^_Eco,PC1_ = 0.80, P < 0.001) and more than the non-parallel interaction terms (non-significant except for PC4) for PC2 to PC4 (S14B Fig). Differentially expressed genes (DEGs) were shared between replicated ecotype pairs significantly more often than expected by chance (Fig 6B), indicating highly parallel divergences in the expression of specific genes (S1 Text, S14C and S14D Fig).

Using a redundancy analysis, we identified 2,921 genes that showed ecotype-associated expression patterns (z-score > 2), and which explained 2.04% of the variation in gene expression (P = 0.008), after correcting for the effect of lake and lineage (Fig 6C). These genes were involved in a range of biological processes, including cell cycle regulation (GO:0007049, Fold-enriched = 32; FDR < 0.001), chromosome organization (GO:0051276, Fold-enriched = 11; FDR < 0.001), chromatin organization (GO:0006325, Fold-enriched = 19; FDR = 0.059) or microtubule-based processes (GO:0007017, Fold-enriched = 19; FDR = 0.013) (S9 Table), which are processes functionally associated with growth, cell differentiation and gene regulation. Furthermore, 162 of these ecotype-associated, differentially expressed genes were located in 13 identified co-expression modules (Total number of genes in modules: N = 806 genes) that were correlated with ecotype divergence for benthivorous-planktivorous ecotype pairs across lakes and lineages (S14E Fig, S10 Table). This further strengthens the importance of expression changes in gene networks and suggests parallel changes in regulatory networks across lakes and lineages.

Although it is known that plasticity plays an important role in the divergence of Arctic charr [46], we found that ecotype-associated expression in white muscle of wild-caught individuals was in part genetically determined. Using a linear modelling approach, we identified a total of 475 *cis*-eQTL (FDR < 0.1), and found that the expression of 25 ecotype-associated genes (0.85%) was significantly associated with *cis-*regulatory variation (*cis*-eQTL; FDR < 0.1; 9 genes at FDR < 0.05; S11 Table), suggesting a parallel regulatory basis. The most significant ecotype-associated *cis*-eQTL included the *COMTD1-*like gene, an enzyme associated with muscle mass in humans [47], *TOMM5*, a crucial protein involved in mitochondrial protein import, and *NR4A1* which is involved in gene expression regulation (Fig 6D–6F; S11 Table).

### Predictability of phenotypic and molecular parallelism

To explore the predictability of evolution by the direction and magnitude of phenotypic parallelism, we examined correlations with intrinsic and extrinsic context, such as genomic differentiation, gene expression and ecosystem size.

**Fig 7:**
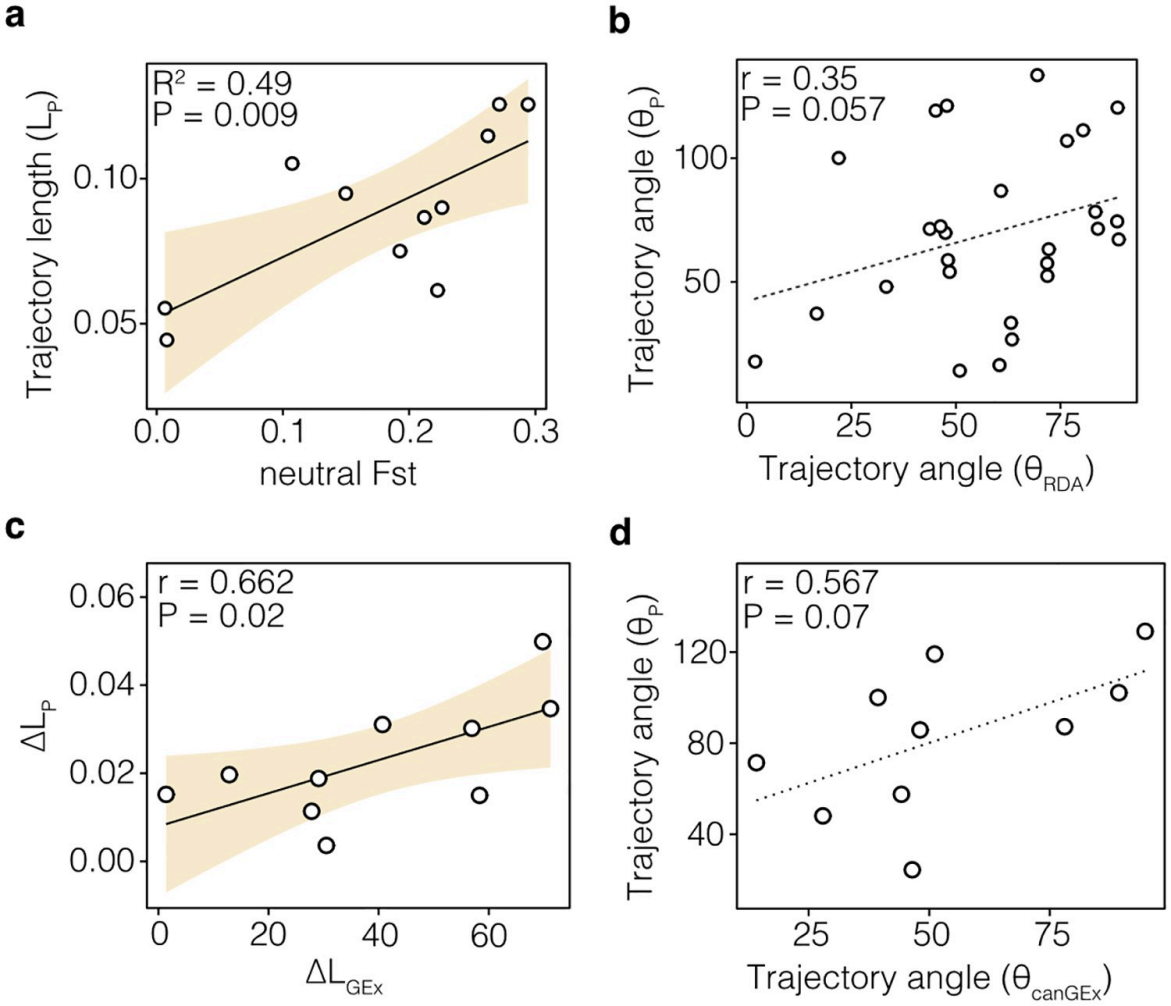
Correlation of gene expression and genetic variation with phenotypic divergence. (**A**) Significant correlation between the degree of neutral genetic differentiation (neutral Fst) and phenotypic divergence (L_P_), with the shaded area highlighting the 95^th^ confidence interval (CI) (N = 11). (**B**) The direction of allele frequency change of ecotype-associated loci (*θ*_RDA_) showed a weak correlation with the direction of phenotypic change (*θ*_P_) across ecotype pairs (N = 27). The dotted regression line shows the weak but non-significant correlation. (**C**) Differences in the magnitude of phenotypic divergence (ΔL_P_) are significantly correlated with differences in the magnitude of gene expression divergence (ΔL_GEx_) between sympatric Arctic charr ecotypes (N = 10). (**D**) The direction of expression trajectories of ecotype-associated genes (*θ*_canGEx_) tends explain the direction of the phenotypic trajectories (*θ*_P_) (N = 10). Each panel shows the linear model or Mantel test result.

The magnitude of phenotypic divergence between sympatric ecotypes was positively correlated with genetic differentiation (Fst_neut_ ~ L_P_: R^2^ = 0.49, P = 0.009; Fig 7A). To remove the effect of population-specific selection and outliers, this Fst_neut_ is based on the 7,179 non-outlier, putatively neutral, loci only (excluding loci with Fst > 95^th^ percentile from the full dataset of 12,215, see *Shared and unique signals of selection*). *Post hoc* exploration of this correlation suggests that genetic differentiation could be partially explained by evolutionary history, because Fst_neut_ was higher in ecotype pairs that diverged under a secondary contact (‘pre-LGM+SC’) scenario (S15A Fig; Fst_neut_ = 0.186, P = 0.034). However, it is difficult to make more detailed inferences because of the imbalance in the number of populations diverged under the two main scenarios (11 ‘pre-LGM+SC’ vs. 2 ‘post-LGM’).

In order to estimate the impact of genetic variation on the direction of phenotypic change, we compared the similarity of allele frequency and phenotypic trajectories across all population comparisons. While the direction of those ‘neutral’ allele frequency trajectories between sympatric ecotypes (N_Total_ = 7,179; S15B Fig) did not correlate with the direction of phenotypic change (*θ*_P_ ~ *θ*_Gn_: mantel r = -0.030, P = 0.544), allele-frequency trajectories of putatively adaptive SNPs (520 SNP identified with RDA) tended to be weakly correlated with the direction of phenotypic trajectories (Fig 7B; *θ*_RDA_~*θ*_P_: mantel r = 0.35, P = 0.057, S15C Fig).

In contrast to genomic changes, magnitude and direction of gene expression divergences between sympatric ecotypes were correlated with phenotypic divergence. Specifically, differences in the magnitude of gene expression divergence (ΔL_GEx_) positively correlated with differences in the magnitude of phenotypic divergence (ΔL_P_ ~ ΔL_GEx_: mantel r = 0.662, P = 0.02; Fig 7C; S15D Fig, S1 Text). The direction of expression divergence of ecotype-associated genes (*θ*_canGEx_) tended to be positively correlated with the direction of phenotypic change (*θ*_P_) (*θ*_P_~*θ*_canGEx_: mantel r = 0.567, P = 0.07; Fig 7D).

**Fig 8:**
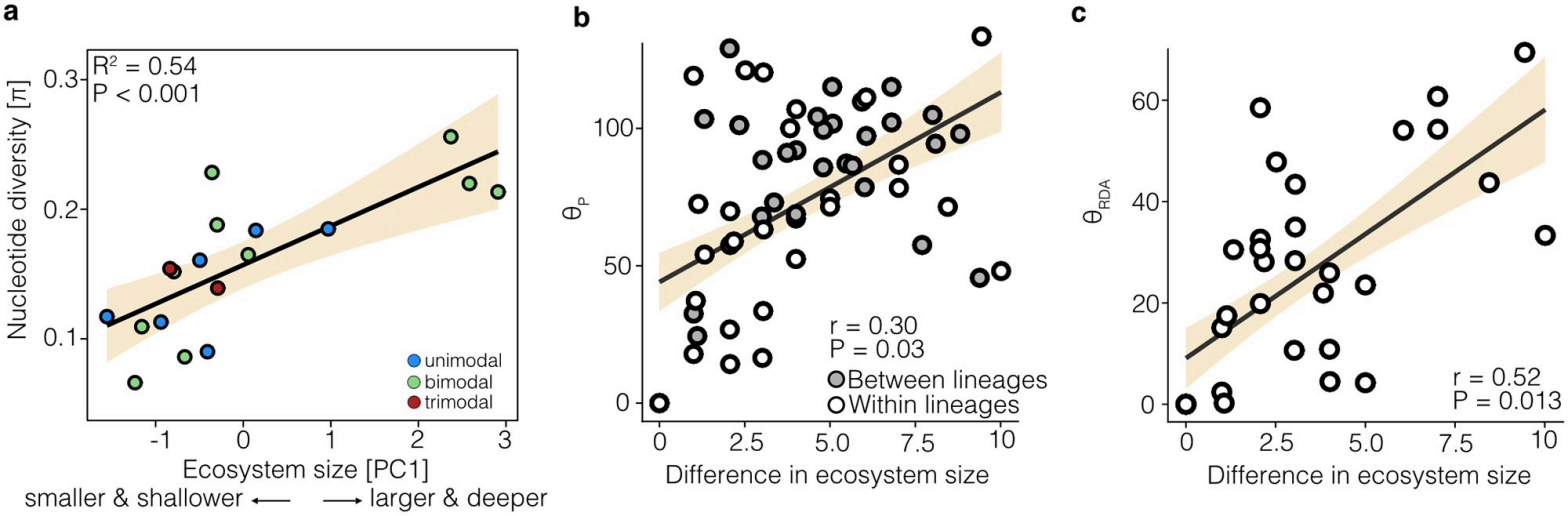
The role of ecological opportunity on divergence and diversity. (**A**) The within-population nucleotide diversity (ecotypes combined by lake) positively correlates with ecosystem size. Points are colored by the number of sympatric ecotypes within each lake. (**B**) The difference in ecosystem size, a proxy for ecological opportunity, explains part of the variation in the direction of phenotypic change (*θ*_P_) between ecotype pairs, within lineages (white points) and across lineages (grey points). (**C**) Similarly, variation in ecosystem size is also correlated with the direction of allele frequency differentiation in putatively adaptive loci (*θ*_RDA_). Each panel shows the linear model or Mantel test result.

Finally, we investigated the explanatory power of ecosystem size, as a proxy for ecological opportunity [48], for the magnitude and direction of phenotypic, genetic and gene expression divergence. We found that the magnitude of phenotypic divergence was not correlated with ecosystem size, meaning that larger, and putatively more diverse lakes did not lead to stronger phenotypic divergence (ΔL_P_ ~ difference in ecosystem size: mantel r = 0.017, P = 0.442). However, in agreement with earlier studies in Arctic charr [49] and Midas cichlids [48,50], we found that the phenotypic variance (mean trait variance ~ Ecosystem size: R^2^ = 0.73, P = 0.001; S15D Fig) and genetic diversity (Ecosystem size ~ π: R^2^ = 0.54, P<0.001; Fig 8A) scaled positively with ecosystem size, suggesting that populations in larger and deeper lakes were more variable. The pattern was particularly influenced by highly genetically diverse populations inhabiting large lakes in Scotland, e.g. Awe and Tay.

We found that variation in ecosystem size was positively correlated with the direction of phenotypic change (*θ*_P_ ~ difference in ecosystem size: mantel r = 0.30, P = 0.031; Fig 8B) and also the direction of genetic change, for neutral SNPs (*θ*_Gn_ ~ difference in ecosystem size: mantel r = 0.36, P = 0.011) and ecotype-associated SNPs (*θ*_RDA_ ~ difference in ecosystem size: mantel r = 0.52, P = 0.013; Fig 8C). Neither differences in the magnitude nor the direction of gene expression divergence were impacted by differences in ecosystem size (ΔL_GEx_ ~ difference in ecosystem size: mantel r = 0.195, P = 0.2; *θ*_GEx_ ~ difference in ecosystem size: mantel r = -0.21, P = 0.72), suggesting that variation in ecosystem size might not affect this gene expression divergence.

## Discussion

With the aim of quantifying the extent of parallel evolution and make inferences about predictability, we integrated phenotypic, genotypic, and molecular data in environmental context for this Holarctic species, Arctic charr. We demonstrated parallel phenotypic changes in several eco-morphological traits and suggest putative drivers and constraints of parallel phenotypic divergence within and across evolutionary lineages. We showed that, while the interaction of genetic differentiation and gene expression divergence determined the magnitude and direction of parallelism, phenotypic divergence is mostly determined by the interaction of environmental variation, putatively adaptive genetic variation, and expression of ecotype-associated genes (Figs 7 and 8). Taken together, our results suggest that repeated selection, particularly on foraging-related traits, led to the parallel evolution of similar eco-morphologies in Arctic charr across geographic and evolutionary scales, and that this occurred in part via similar genetic and molecular pathways.

### Phenotypic parallelism within and across evolutionary lineages

We detected substantial variation in the extent of parallelism for overall eco-morphology (based on trajectory analyses, Fig 2B) and for specific traits across independently replicated Arctic charr ecotype pairs (Fig 2C and 2D). The extent of phenotypic parallelism for specific traits, as described by the variation explained by the ecotype term (η^2^_eco_), ranged from weak parallelism in head length (η^2^_eco_ < 0.1) to medium-strong parallelism in eye diameter (η^2^_eco_ = 0.31–0.57) (Fig 2B and 2C), as classified by Oke *et al* [10]. Foraging and habitat related traits such as eye diameter and head depth had been suggested to be highly divergent [34,35,51], parallel [27,34], and partially heritable [33,39] in Arctic charr, further suggesting that these traits are under strong repeated natural selection and crucial for the adaptation to replicated trophic niches. Our finding is in agreement with several previous studies on parallel evolution that described a continuum of phenotypic divergence across populations (e.g. for fishes [10,12,38]).

However, most previous studies of phenotypic parallelism have focused on evolutionarily and/or geographically limited comparisons, such as lake-stream or benthic-pelagic sticklebacks from British Columbia [12,25], Trinidad guppies [10], or Midas cichlids in Nicaraguan crater lakes [7,50], potentially leading to generous estimates of parallelism because the effects of evolutionary contingency or environmental stochasticity will be likely minimised in those cases. In our study we detect substantial parallelism in specific traits but also in overall phenotypic divergence across these evolutionarily and geographically distinct lineages (Fig 2). For example, we found benthivorous-planktivorous ecotype pairs from Dughaill in Scotland and Kamkanda in Transbaikalia were highly parallel (*θ*_P_ = 24.4°; ΔL _P_ = 0.0152). In fact, phenotypic trajectories for replicated Arctic charr ecotype pairs (Fig 2B) were overall more parallel than those reported for global lake-stream sticklebacks [12,52]. Together, these results suggest that repeated natural selection can lead to phenotypic parallelism in both single phenotypic traits and overall eco-morphology, even across evolutionary and geographically distinct lineages that have been separated for more than 60,000 years. Yet, deviations from parallelism provide insights into the effects of environmental stochasticity and evolutionary contingency on repeated phenotypic divergence.

### Potential drivers and constraints of phenotypic parallelism

Phenotypic parallelism between independently evolved populations has been thought to support a deterministic role of natural selection (reviewed in [10]) with deviations suggesting influences of stochasticity and contingency [67], though this has rarely been quantified. Here, we explored deviations from phenotypic parallelism to identify the potential drivers and constraints of parallel evolution in this broad context. We found that the magnitude of phenotypic divergence across ecotype pairs could be predicted by their neutral genetic differentiation (Fig 7A). A similar pattern shown for lake-stream stickleback pairs on Vancouver Island [12] was speculated to be due to the reduction of adaptive phenotypic divergence in pairs with more gene flow. Using coalescent models we showed this historical effect on contemporary phenotypic and genotypic differentiation. Specifically, we found that Arctic charr ecotype pairs that likely diverged under gene flow (isolation with migration), rather than having a period of historical isolation, had less genetic differentiation and less phenotypic divergence (S15A Fig, S6 Table). In addition, ecotype pairs that showed lower gene expression divergence were also less diverged in overall eco-morphology (Fig 7C), though with these data we cannot determine how gene expression in this tissue contributes to phenotypic divergence. These results suggest that variation in genetically and environmentally mediated molecular responses facilitate stronger adaptive divergence.

In contrast to magnitude, the direction of phenotypic change was most strongly associated with environmental differences across populations, namely ecosystem size (Fig 8A). Ecosystem size has been suggested to be a good proxy for ecological opportunity in lakes, where larger ecosystems (by lake depth and surface area) harbour more ecological niches [48]. Ecosystem size has been shown to correlated with phenotypic variance in Arctic charr [49] and Midas cichlids [48,50] as well as the extent of planktivorous specialisation in Arctic charr from Greenland [53], supporting our findings that larger lakes support more diverse population (Fig 8). Variation in ecosystem size, and thus ecological opportunity and environmental variation, is potentially associated with context-dependent natural selection, therefore leading to different adaptive optima [68]. This is consistent with the correlation between ecosystem size and the direction of allele-frequency change in ecotype-associated SNPs (Fig 8C), which suggests that environmental differences partly determine adaptive genetic responses and thereby lead to parallel phenotypic responses (Fig 7B). Similar findings have been described in lake-stream and marine-freshwater stickleback, with fine-scale environmental variation determining the direction of genetic and phenotypic change [12,54]. This suggests that ecosystem size is potentially a good estimator of ecological opportunity and environmental variation in postglacial lake systems, and that environmental context strongly impacts parallel evolution.

We found that the direction of phenotypic change was additionally correlated with variation in gene expression (Fig 7D), suggesting that similar molecular pathways translate parallel environmental pressures and genetic responses into parallel phenotypic outcomes. Ecotype pairs that were more similar in the expression of ecotype-associated genes, but not overall expression, tended to be more similar in their direction of phenotypic divergence. Patterns of gene expression variation were not correlated with differences in ecosystem size, suggesting that environmental variation related to ecosystem size, i.e. size of the littoral or pelagic zone, does not affect gene expression variation. We speculate that gene expression could compensate for variable genomic underpinnings and facilitating phenotypic parallelism. More research in this area is needed for natural populations.

Our findings suggest that the interplay of environmental variations, stochasticities, and molecular contingencies—for example as evidenced by differences in the direction of adaptive genetic responses—likely shape the extent of phenotypic parallelism in Arctic charr and potentially other postglacial fishes.

### Molecular parallelism within and across evolutionary lineages

Contrary to the phenotypic and gene expression patterns, few regions of the genome were shared outliers between ecotype pairs across replicates (Fig 5A) and genetic parallelism within and across evolutionary lineages was variable, as expected given the continuum of phenotypic parallelism and the relatively recent divergence of populations [13,55]. We used a range of approaches to infer genomic patterns. While the sharing of Fst outlier SNPs (i.e. the top 5% Fst distribution) was low and non-significant (Fig 5A), we found that many SNPs showing elevated differentiation were shared across ecotype pairs. This can be explained by the fact that our reduced-representation sequencing approach did not detect causal SNPs but instead linked SNPs showing elevated differentiation due to hitchhiking [45,56]. As the rate of LD decay differs around outlier loci and demographic processes will vary across ecotype pairs, the level of genetic differentiation in linked SNPs is expected to differ across populations. Consequently, not all SNPs linked to the same causal locus will be statistical outliers (i.e. above the 95^th^ percentile) but will tend to show elevated differentiation compared to the genomic background [45]. This suggests that genomic responses to selection in these replicate populations are at least partially parallel.

The significant but low number of SNPs that showed repeated ecotype-associated allele-frequency divergence in both evolutionary lineages suggests that inter-lineage genetic parallelism is relatively low. However the same SNPs or genomic regions are associated with repeated ecotype divergences in at least some populations even across lineages, evident by more permissive analytical approaches that did not require a SNP to be divergent between ecotypes in all pairs [57]. These identified significant signals of parallel selection across the genome (Fig 5B) and significant allele-frequency associations with ecotype across lakes within lineages (Fig 5C). The correlation we detected between ecotype-associated loci and the direction of phenotypic change within and across lineages (Fig 7B) suggests the adaptive role of shared genetic variation across lineages. Partial reuse of the same genomic regions across evolutionary lineages may suggest certain constraints on the genetic architecture of adaptive trait divergence and a limited amount of ecotype-associated standing genetic variation. High genetic redundancy in genetic architectures and large amounts of genetic variation would likely lead to lower genetic parallelism [58,59]. More high-density genomic data will be needed to determine the level of genomic parallelism in this system robustly.

Evidence from theory and empirical studies suggests that the extent of genomic parallelism is impacted by factors such as genetic co-ancestry and shared genetic variation [60], demographic history [13], and similarity in selection pressures [52]. Indeed, the higher parallelism of ecotype-associated SNPs within lineages compared to across lineages in these Arctic charr replicates likely reflects the role of genetic co-ancestry and shared adaptive genetic variation in genetic parallelism, in line with observations in other systems [60,61]. The influence of different pre- and postglacial demographic histories is also evident in Arctic charr. For example, whether the mode of speciation was inferred to be secondary contact with admixture or isolation-with-migration is reflected in the contemporary genetic differentiation and phenotypic divergence in Arctic charr; ecotype pairs that likely diverged from a common gene pool following secondary contact and admixture of refugial lineages, e.g. the Siberian populations, are more differentiated (S15A Fig) [34,40]. Finally, similar selection pressures found in similar environments are expected to lead to a component of parallel genetic responses, for example through the use of shared standing genetic variation even across lineages [13,30]. Here we suggest this is reflected in genetic parallelism of ecotype-associated loci correlated with ecosystem size (Fig 8B). Partial genomic parallelism has been detected in other postglacial fishes, such as sticklebacks and lake whitefish [55,62], suggesting that a mixture of parallel and non-parallel genomic responses may be common. Large-scale comparative or experimental studies across species will be needed though to tease apart the effects of environment and genetic background on genomic parallelism.

In contrast to the genomic patterns, we found gene expression divergence to be highly parallel between replicated ecotype pairs both within and across evolutionary lineages (Fig 6A and 6B) We speculate that differential gene expression might facilitate parallel phenotypic evolution despite variation in genetic parallelism in these replicate divergences of Arctic charr, as has been suggested in *Littorina* snails [63] and Australian groundsel [64]. Co-expression networks of ecotype-associated genes were associated with a range of biological processes such as metabolism, growth, cell differentiation and gene regulation, potentially explaining the observed differences between sympatric ecotypes in morphology and body size, as well as swimming and foraging behaviour [27,35,39]. These are prime candidates for future research on functional genomics of parallel evolution in freshwater fishes.

Like morphology, gene expression is affected by both environment and genotype. Therefore, the component of parallelism in gene expression could be influenced by similar short-term plastic responses to these similar environments [46]. However, we found the expression of several ecotype-associated genes in benthivorous-planktivorous ecotypes pairs to be regulated by shared *cis*-eQTL, even across evolutionary lineages (Fig 6D–6F, S11 Table). This suggests that parallel gene expression divergence is in part controlled by a parallel genetic basis, which has also been shown for parallel intercontinental benthic-pelagic divergences in whitefish [65]. Due to the relatively low sample size we likely only detected the strongest *cis*-eQTLs and underestimated the true number of genes with genetically mediated differential expression. In-depth functional analyses and common garden experiments will be needed to tease apart the effects of environment and genotype on parallel gene expression regulation in Arctic charr and other salmonids.

Overall, we showed that putatively adaptive genetic and molecular parallelism exists within and even across lineages but is variable. We suggest this reflects contingencies of environment and history that influence contemporary phenotypic parallelism. Nonetheless replicated contexts of natural selection facilitating divergence is suggested by parallel genomic responses to ecosystem size. We propose that gene expression is a bridge that facilitates parallel evolution of ecotypes, potentially buffering environmental stochasticities and evolutionary contingencies such as variation in environment, genomic divergence, and evolutionary history.

### Conclusion

The evolution of replicated ecotypes has long fascinated naturalists and evolutionary biologists, as it indicates the predictable action of natural selection [66]. Our study demonstrates components of phenotypic parallelism in these replicate divergences of Arctic charr ecotypes within and across evolutionary lineages. We identified components of the genome that are shared and associated with ecotype. We suggest that the extent of parallelism in phenotype and genotype is influenced by stochasticity and contingency, such as environmental variation, demography, and evolutionary history. Some of these influences can be quantified using integrated studies of parallel evolution, allowing better prediction of evolutionary trajectories and response to environmental change. These repeated divergences of Arctic charr provide an example of how replaying the tape of life can lead to repeated and predictable outcomes, contrary to Gould’s predictions [67], but also illuminates the variable routes and mechanisms leading to parallel adaptations.

## Materials and methods

### Ethics statement

Fish collection was undertaken under licence from Marine Scotland (CEA) and with local permissions (CEA, SSA).

### Arctic charr sampling

Fish were sampled from nine Scottish lakes (Atlantic lineage) and nine Transbaikalian lakes (Siberian lineage) [36], between 1995 and 2015 using standard gill nets (Fig 1A). The sampled lake populations (we refer to all individuals within a lake as a population) contained different numbers and combinations of ecotypes (we refer to trophic specialists as ecotypes). We classified individuals into four ecotypes based on their primary diet (see [26,27,34,35,40] for details): 1) Planktivorous–which feeds mainly on plankton throughout the year, 2) Benthivorous–which consumes a substantial proportion of benthic invertebrates, particularly during autumn and winter, 3) Piscivorous–which feeds mainly on other fish, 4) Insectivorous—which feeds largely on postlarval stages of insects and 5) unimodal-planktivorous—which represent non-diverged mainly plankton-feeding populations used as outgroups. After collection, we photographed the left side of each fish (Atlantic samples), or individual fish were preserved in formaldehyde for subsequent phenotypic analysis (Siberian samples). White muscle tissue (from underneath the dorsal fin and above the lateral line) and/or fin clips were taken for subsequent genetic and transcriptomic analysis and stored in absolute ethanol or RNAlater at -20°C. Fish collection was undertaken under license from Marine Scotland and with local permissions.

### Analysis of linear morphological traits

Eco-morphological analysis was performed based on seven linear traits, on 345 individuals from the Atlantic lineage and 984 individuals from the Siberian lineage (S1 Table). Seven linear measurements and fork length were taken from photographs using ImageJ v. 1.50i [68] for Atlantic samples or directly from formaldehyde fixed fish for Siberian samples (Fig 2B) [27]: FL–fork length, HDO–head depth at operculum, HDE–head depth at eye, HL–head length, ED–eye diameter, ML–maxilla length, LJL–lower jaw length, PFL–pectoral fin length. Linear traits were chosen based on previous studies on eco-morphological divergence in salmonid fishes and their potential functional importance [27,31,39,69]. Linear traits were correlated with body length, and therefore scaled to mean fork length, using the allometric formula as described in [70]: log^10^ Y_i_ = log^10^ M_i_ + *b* * (log^10^ Lm—log^10^ L_i_); where Y_i_ is the corrected trait value, M_i_ is the measured trait value, *b* is the slope (regression coefficient) of the regression of the trait value against fork length (L_i_) within each lake and ecotype, and L_m_ is the mean fork length of all fish within a lineage. The slope was calculated using population and ecotype as covariates. Size-adjusted measurements were used for all subsequent analyses. Principal component analyses (PCA) were used to uncover the major axes of phenotypic variation in the Atlantic and Siberian lineages, using the *ppca* approach in *pcaMethods* (R package) to account for missing data.

### Analysis of phenotypic parallelism based on linear traits

To determine the contribution of parallel and non-parallel aspects to the overall morphological divergence of ecotypes, within and across populations, we used the ANOVA (multivariate analysis of variance) approach outlined by Langerhans and DeWitt [17]. ANOVAs (trait/PC ~ ecotype + lake + lineage + ecotype x lake + ecotype x lineage) were performed for both lineages combined using individual principal component (PC) scores (PC1 to PC4) and individual linear traits to test for the extent of parallel (ecotype effect) and non-parallel (ecotype x lake interaction (E x L); ecotype x lineage (E x Lin) interaction) phenotypic divergence of sympatric ecotypes across lakes and lineages, and the effect of the unique evolutionary history of each ecotype pair (lake effect and lineage effect) on phenotypic variation across lakes and lineages. We used the *EtaSq* function in *BaylorEdPsych* (R package) to estimate the effect size (Wilk’s partial η^2^) of each model term for linear traits and principal component scores. Traits and PCs for which the ecotype term has the largest effect size are highly parallel between ecotypes across lakes and lineages. Those traits and PCs for which the ecotype term explains more variation than the interaction terms (which indicate non-parallel patterns of divergence in magnitude and/or direction), but not more than the lineage and lake terms, are to some degree parallel but are strongly influenced by differences in the evolutionary history between lake populations or lineages.

Furthermore, we performed a complementary phenotypic trajectory analysis (PTA) [16] to quantify the level of parallelism and deviations from parallelism based on all linear traits combined. The PTA was conducted using the *trajectory*.*analysis* function in *geomorph* (R package). The significance of differences in trajectory directions (*θ*_P_: differences in the direction of phenotypic change) and trajectory lengths (ΔL_P_: differences in the magnitude of phenotypic change) was assessed using 1,000 permutations. Ecotype pairs were considered parallel if the angle between trajectories and the differences in trajectory lengths were not significantly different from zero (see [11,12] for details).

### Chromosome assembly and annotation of Arctic charr draft genome

We created a draft chromosome-level assembly of the Arctic charr genome based on an Arctic charr linkage map [71] and synteny with the Atlantic salmon reference genome [72] based on *Chromosomer* analysis [73]. The assembly was created using *All-Maps* [74]. We created a draft annotation using *GeMoMa* 1.4.2 [75] and the quality of gene predictions was evaluated using *BUSCO* v.1.22 [76]. Arctic charr and zebrafish (*Danio rerio*) orthologues were identified using *Orthofinder* [77] (see S1 Text for details). The present analyses are based on an early assembly of the Arctic charr genome and an updated chromosome-scale assembly of the Arctic charr genome was recently published [78].

### DNA extraction and ddRADseq

DNA was extracted from fin clips and muscle tissue using the NucleoSpin Tissue kit (Macherey-Nagel), following the manufacturers recommendations. DNA quality and quantity were assessed using agarose gel electrophoresis and the Qubit Fluorometer with the dsDNA BR Assay (Life Technologies). ddRADseq libraries were prepared using a modified version of the Recknagel *et al*. (2015) [79] ddRADseq protocol for Illumina sequencing platforms. Paired-end 75-bp sequencing was performed on the Illumina NextSeq500 platform at Glasgow Polyomics (University of Glasgow) at 3-4M read coverage per individual.

### Amplification, sequencing and analysis of the mitochondrial ND1 gene

The mitochondrial ND1 gene was amplified for 107 individuals (between 2 and 11 individuals per population) using the primer-pair B1NDF/B1NDF and PCR conditions as described in Schenekar et al. (2014) [80]. The PCR product was cleaned, and Sanger-sequenced in both directions at *DNA Sequencing and Services* (MRC I PPU). Contigs were assembled from forward and reverse reads using *Sequencher* v.5.4 (http://www.genecodes.com/) after removing low quality reads and trimming read ends. Reads for all individuals were aligned using *Muscle* in *MEGA* v.7 [81] and trimmed to a common length. A TCS haplotype network was built in *POPART* [82].

### Processing of ddRADseq data

Raw sequence data quality was assessed using *FastQC v*.*0*.*11*.*3* (http://www.bioinformatics.babraham.ac.uk/projects/fastqc). The *process_radtags* pipeline in *Stacks* version 1.46 [83] was used for demultiplexing raw sequence data based on unique barcodes, quality filtering and read-trimming to 70bp of all libraries. Processed reads were aligned to the Arctic charr draft genome with *bwa mem* v.0.7.15 using a seed length of 25bp and the–M option. Reads with mapping quality <20 were removed using *samtools* v.1.6. We used *Stacks* v.1.46 and the *ref_map*.*pl* pipeline for building RAD-loci and SNP calling. The *populations* module was used to export genotype calls in VCF format for further filtering in *vcftools* v.0.1.15. We created three different datasets; a global dataset for the Atlantic and Siberian lineages combined, and separate datasets for each lineage. SNPs were retained when the following criteria were fulfilled: (i) present in at least 66% of all individuals within a population and 2/3 of all populations, (ii) global minor allele frequency (MAF) ≥ 0.05 or ≥ 0.01 for the global dataset, (iii) heterozygosity ≤ 0.5 (iv) in Hardy-Weinberg equilibrium (P > 0.05) in at least 2/3 of all populations and (v) with a minimum coverage of 6x. Filtering and conversion of data into the different formats was performed using *Stacks*, *vcftools*, *PLINK* v.1.90 and *PGDspider* v.2.11.2. Datasets for creating site frequency spectra were filtered in a similar way, except no MAF cut-off was used in order to retain informative low frequency sites and a maximum of 10% missing data within each dataset (e.g. each ecotype pair) was allowed. For all analyses, only one SNP per locus was retained to reduce the effect of linkage.

### Summary statistics and analysis of population structure

To assess population structuring across and within lineages, and within lakes, we applied multiple approaches using the global and lineage-specific SNP datasets. First, we used PCA in *adegenet* (R package) to assess major axes of genetic variation. Second, *Admixture* v.1.3 [84] was used with a ten-fold cross-validation to detect the most likely numbers of clusters and genetic ancestry proportions within lineages. *Genodive* v.2.0b27 [85] was used to estimate pairwise genetic differentiation (Weir-Cockerham Fst) between ecotypes within and across lakes using the global dataset, with 10,000 permutations. We calculated genome-wide nucleotide diversity for each ecotype for all populations based on all SNPs using *vcftools*. We assessed the relationship among populations within and across lineages using a neighbour-joining splits network using *SplitsTree4 v*.*4*.*14*.*4* [86]. To assess genetic co-ancestry among individuals within and across lakes, we used haplotype-based population inference approach implemented in *fineRADstructure* v.0.1 [87], using the same filtering criteria described for the SNP dataset. Analyses were performed using default settings.

### Introgression and differential admixture

We used *Treemix* v.1.13 [88] to explore and visualize secondary gene flow within each lineage. We built maximum-likelihood trees for non-admixed individuals (admixture threshold of 0.25 as inferred with *Admixture*) to reduce the effect of contemporary admixture. We fitted up to six and ten migration edges for the Atlantic and Siberian populations, respectively, and chose the most likely migration events based on the maximized variance explained, maximum-likelihood and the significance of each migration edge. To formally test for introgression in a four-population tree (‘deviation from tree-ness’) we used *f4-statistics* implemented in the *fourpop* function of *Treemix* and D-statisitcs (Abba-Baba test) implemented in Dsuite [89]. We used maximum likelihood trees for each lineage for the Abba-Baba test rooted with a single population from the other lineage (Davatchan and Dughaill). We focused on the Tree-based estimate of the D-statistic but report the Dmin-based and BBAA-based estimates for completeness. Furthermore, to test for significant admixture within a population in a three-taxon comparison (Target taxon C; Reference taxon A, Reference taxon B) we used *f3-statistics* as implemented in the *threepop* function in *Treemix*.

### Inference of evolutionary histories

To distinguish between alternative evolutionary scenarios leading to ecotype diversity within lakes, we used coalescence simulations implemented in *fastsimcoal2* v.2.5.2.3 [43] and information contained in the multidimensional site frequency spectrum (SFS). 2-population and 3-population SFSs were created using *∂a∂i* v.1.6.3 [90] for each population and parapatric outgroups if appropriate. Populations were downsampled by around 30% to reduce the effect of missing data. The minor folded site frequency spectrum was used due to the lack of a trinucleotide substitution matrix for salmonids and sequencing data for outgroup species. To determine absolute values for divergence times and other inferred parameters, we corrected the number of monomorphic sites in the SFS [91,92]. In brief, since only one SNP per RAD tag is retained, the ratio of SNPs to invariant sites in the spectrum is skewed. Thus, one has to calculate the actual ratio of SNPs to invariant sites in the initial dataset and use that to adjust the number of monomorphic sites [92]. A mutation rate of 1x10^-8^ was used as no known mutation rate for Arctic charr or salmonids was available [93].

For all sympatric ecotype pairs, seven pairwise demographic models describing different historical divergence scenarios were tested (S6 Fig): Strict isolation (SI), Ancient migration (AM), Isolation-with-migration (IM), Secondary contact (SC), Secondary Contact with introgression (AdmSC), Isolation-with-migration with a historical change in migration rate (IMchange) and an IM-model with a historical introgression event between sympatric ecotypes (IMint) were tested. In lakes with three sympatric ecotypes (Kamkanda and Kalarskii Davatchan), or strong admixture across lakes (Loch Dughaill and Loch Uaine), models describing different combinations of strict isolation, isolation-with-migration, secondary contact, introgression and hybrid speciation were tested for all three ecotypes/populations together. These models tested in general two different evolutionary histories. The isolation-with-migration models test a history of divergence under constant gene flow (with differing rates), whereas secondary contact models mainly test the occurrence of historical secondary contact between different distinct lineages or populations (e.g. distinct gene pools or glacial refugial populations) prior to ecotype divergence.

We ran a total of 30 iterations for each model and lake, and selected the most likely model based on the AIC [43]. Each run consisted of 40 rounds of parameter estimation with 100,000 coalescent simulations. We only used variants with a minimum count of 2 in the SFS to filter out low frequency variants. Point estimates of inferred parameters were taken from the most likely model and averaged over the top five runs.

### Patterns of selection and differentiation

To determine outlier loci between sympatric ecotypes, we screened the genome for loci showing genetic differentiation (Weir-Cockerham Fst calculated using *vcftools*) above the 95^th^ quantile of the Fst distribution (outlier loci). We estimated the number and proportion of shared outlier SNPs and contigs containing outlier SNPs (but not the same SNPs across contigs) across benthivorous-planktivorous and piscivorous-planktivorous ecotype pairs. To determine if more outlier SNPs or contigs are shared among two ecotype pairs than expected by chance, we used *resample* (R package) to resample n number of SNPs (n = number of outlier SNPs for each ecotype pair) 10,000 times with replacement from the full dataset and determined the mean number of shared SNPs from that distribution. We calculated proportion-based p-values based on the number of observed and expected shared outlier SNPs/contigs and the total number of SNPs for each pairwise comparison using R function *prop*.*test*. We also plotted the standardised Fst (ZFst) against delta nucleotide diversity (Δπ = π_ecotype2_ − π_ecotype1_) between sympatric ecotypes, to determine if outlier loci show reduced genetic diversity in one or both ecotypes, potentially indicating selective sweeps.

Furthermore, to assess the impact of differences in evolutionary history and demography on our ability to detect Fst outlier loci, we implemented a custom permutation approach. To obtain a Fst null distribution under panmixia, we randomly assigned ecotype labels to individuals within lakes and then calculated Fst using *vcftools*. We repeated this analysis 1,000 times and calculated the null distribution of Fst values and the 95^th^ percentile for each ecotype pair. Subsequently, we identified outlier loci as those with empirical Fst values above the permutated 95^th^ percentile and repeated the outlier sharing analyses (above) for this ‘permutated outlier SNP set’. We only repeated the analysis for individual SNPs and not shared contigs.

Additionally, we used a hierarchical implementation of bayescan to jointly test for signals of parallel selection across replicated ecotype pairs within lineages using default settings [57].

### Genome-wide association analysis

To detect loci significantly associated with ecotype within each lineage we used a redundancy analysis (RDA), controlling for the effect of lake (condition) using *vegan* (R package). Ecotypes were coded numerically, with planktivorous as 0, benthivorous as 1 and piscivorous as 2. We excluded unimodal populations and ecotypes from Tokko from this analysis. SNPs were selected as significantly associated with ecotype if the z-transformed loading for RDA1 (and RDA2 in the Siberian population) was above 2 or below -2 (equivalent to a two-tailed p-value < 0.05). To test if more ecotype-associated SNPs were shared across the lineages, we used the same resampling approach as for the outlier SNPs.

We imputed missing data in each SNP dataset using the *LD-kNNi* method implemented in *Tassel5* [94], based on the 10 closest genotypes using the default settings. To test the imputation accuracy, we calculated Pearson’s correlations between allele frequencies before and after imputation for the full dataset and the subset with the highest proportion of missing data.

### RNAseq and processing

Total RNA was extracted from white muscle tissue from 44 individuals (N = 4 per ecotype per lake) from five lakes (Awe, Tay, Dug, Kam, Tok), representing all possible ecotypes (benthivorous, planktivorous, insectivorous, piscivorous), using PureLink RNA Mini kits (Life Technologies, Carlsbad, CA). Both sexes were sampled and used in roughly equal ratios. Extractions were carried out following the manufacturer’s instructions, with the exception of an additional homogenization step using a FastPrep-24 (MP Biomedicals) prior to isolation. RNA quantity and quality were assessed using the Qubit 2.0 fluorometer (Life Technologies, Carlsbad, CA) with HS Assay kits and a 2200 Tapestation (Agilent, Santa Clara, CA), respectively. High quality RNA was achieved, with A260/280 ratios between 1.9 and 2.1 and RNA Integrity Numbers above 8.3.

RNA-seq libraries were prepared and sequenced at Glasgow Polyomics (University of Glasgow) for Awe, Tay, Dug and Kam and at BGI (Shenzhen, China) for Tok. Individual cDNA libraries were prepared for each individual using the TruSeq Stranded mRNA Sample Preparation kit (Illumina, San Diego, CA) in combination with a Poly-A selection step. Libraries were sequenced on an Illumina NextSeq 500 (Illumina, San Diego, CA) using 75-bp paired-end sequencing, at a depth of 25-30M reads per library. Raw reads were processed using *Scythe v0*.*9944 BETA* (https://github.com/vsbuffalo/scythe/) and *Trimmomatic v0*.*36* [95]. Leading and trailing bases with a Phred quality score <20 were removed and a sliding window approach (4 bp window size) was used to trim reads at positions with Phred scores below 20. A minimum read length of 50 bp was allowed. We used *FastQC v0*.*11*.*2* to assess read quality before and after processing. Processing removed ~2% of reads, resulting in 1.81 billion cleaned reads. The resulting reads were aligned against the Arctic charr draft genome using *STAR v*2.5.2b [96], with default parameters. Raw reads were counted for each gene based on the longest isoform annotation using the HTSEQ-count python package [97] with the unstranded (—stranded = no), CDS–based (—type = CDS,—idattr = Parent) settings. Only genes with at least 20 read counts per lake were used for further downstream analyses.

### Gene expression analysis

To identify the major axes of expression variation across lakes, we performed a principal components analysis using the *svd* PCA approach in *pcaMethods* (R package) based on rld-transformed gene expression data (transformed using the *DEseq2* R package) [98]. The raw read count table was used for the differential gene expression analysis between sympatric ecotypes using *DESeq2* on a per lake basis. Furthermore, to identify genes with ecotype-associated expression patterns, we performed a RDA on rld-transformed count data using the *vegan* R package controlling for lineage and lake (conditions). We again used z-transformed loadings above 2 or below -2 as the significance threshold.

To further examine the functional bases of trophic divergence Arctic charr, we used a Weighted Gene Co-Expression Network Analysis (WGCNA) to identify co-expressed gene modules [99]. Network analyses were only performed on benthivorous–planktivorous ecotype pairs (from Awe, Tay, Dug and Kam; N = 32, four per ecotype, per lake). To reduce stochastic background noise from lake-specific effects in our expression data, we used a linear mixed model in *variancePartition* (R package) to identify a subset of genes with expression variation attributed to ecotype (see SI for detail). All genes for which ‘ecotype’ explained more than 10% of the total expression variation across individuals were used for network construction. A single network was constructed for all 32 samples and 1,512 ecotype-associated genes, from the log_2_ scaled count data (DESeq2: *rlog*), using *WGCNA* (R package), following the standard procedure. Network modules were defined using the dynamic treecut algorithm, with a minimum module size of 25 genes and a cut height of 0.992. The module eigengene distance threshold was set to 0.25 to merge similar modules. To determine the significance of module-trait relationships, Pearson’s correlations were calculated between module eigengenes (the first principal component of the expression profile for a given module) and lake and ecotype. P-values were Benjamini-Hochberg corrected (FDR<0.05).

### *Cis-*eQTL mapping

To determine if the expression of candidate genes is genetically determined we performed *cis*-eQTL mapping using all benthivorous-planktivorous ecotype pairs (N = 32). First, we called SNPs from reference-aligned RNAseq data using *freebayes* (https://github.com/ekg/freebayes), after marking duplicates using *picard*, with a coverage threshold of three. We only retained biallelic SNPs with a phred quality score above 30, a genotype quality above 20 and an allele-depth balance between 0.25 and 0.75. Furthermore, we filtered for Hardy-Weinberg disequilibrium (p-value threshold < 0.01), and only kept sites that were present in at least 90% of all individuals across populations. The filtering was performed using the *vcffilter* command implemented in *vcflib* and *vcftools*. Using these filtering steps, we retained 12,393 SNPs.

To associate gene expression with sequence polymorphisms and identify *cis*-eQTL, we used *MatrixEQTL* v.2.2 (R package) [100]. We used a linear model with lake and lineage as covariates, and a maximum distance of 1 Mbp between SNP and differentially expressed gene (cis-acting polymorphism only). *Cis*-eQTL were identified with a false-discovery rate (FDR) below 0.1 after correcting for multiple testing. Due to the low sample size and low statistical power, we focused our interpretation only on *cis*-eQTL for ecotype-associated genes (identified using RDA based on gene expression). However, we also report results for an FDR of 0.05.

### Characterisation of differentially expressed genes

To detect genetic pathways associated with ecotype-associated differentially expressed genes (identified using RDA) and co-expressed gene modules, we performed overrepresentation analyses using the *WebGestalt* tool [101]. We only used Arctic charr genes which had 1:1 or 2:1 orthologs in the zebrafish genome, with the following settings in Orthofinder: minimum number of genes for a category = 5, maximum number of genes for a category = 500, number of permutations = 1000, number of categories with leading-edge genes = 20, KEGG pathways, organism = *Danio rerio*.

### Gene sharing between comparisons

To calculate the expected number of shared differentially expressed genes (DEGs) between comparisons we used a permutation-based approach, similar to the outlier comparison, with 10,000 permutations. We randomly sampled N genes (N = the number of DEGs in a comparison) 10,000 times from each dataset with replacement and calculated the expected number of shared DEGs as the mean number of shared resampled genes in each comparison. We calculated proportion-based p-values based on the number of observed and expected shared DEGs, and the total number of genes for each pairwise comparison using R function *prop*.*test*.

### Trajectory and regression analysis

Similar to the phenotypic trajectory analysis, we performed trajectory analyses (TA) based on different genetic datasets and gene expression data. The TA was performed using the *geomorph trajectory*.*analysis* function based on PC scores derived for each dataset. For the genetic data, we calculated trajectory lengths and angles for all neutral SNPs (N = 7,179 SNPs, PC1-6; *θ*_Gn_, and ΔL_Gn_) and ecotype-associated SNPs (N = 217 SNPs in the Atlantic lineage, PC1-4; N = 582 SNPs in the Siberian lineage, PC1-6;*θ*_RDA_, ΔL_RDA_). Except for the ecotype-associated SNPs, the TA was performed for both lineages combined. We also performed TA based on PC1-6 for all expressed genes (*θ*_GEx_, ΔL_GEx_) and for PC1-5 based on all genes associated with ecotype in the RDA (*θ*_canGEx_, ΔL_canGEx_). In all cases, we selected all PCs that cumulatively explain more than 50% of variation.

To identify how the different factors (phenotype, genotype, evolutionary history and gene expression) are correlated, we performed linear regression analyses using the *lm* function (for independent datasets) and Mantel tests (for non-independent datasets; Pearson correlation) in *vegan* using the different input datasets. First, we compared how differences in the angles between trajectories and lengths of trajectories, calculated based on different datasets (linear traits (*θ*_P_, ΔL_P_), neutral SNPs, ecotype-associated SNPs, overall gene expression and ecotype-associated expression), are correlated using Mantel tests. Furthermore, we determined how absolute magnitudes of divergence (absolute length of trajectories for the same datasets (L), and neutral and outlier-based Fst) are correlated using linear regressions.

Furthermore, we estimated the correlation between ecosystem size and trajectory lengths and directions. We used the first PC from a PCA performed using *prcomp* based on maximum lake depth and surface area, estimated using Google Earth Pro, as a proxy for ecosystem size [49]. We then calculated the Euclidean distance between PC1 scores for each pairwise comparison as a proxy for environmental distance, and tested its correlation with differences in trajectory lengths (ΔL) and trajectory directions (*θ*) using Mantel tests for phenotypes, neutral SNPs, ecotype-associated SNPs, gene expression and ecotype-associated gene expression. We also tested the effect of ecosystem size (PC1) on population nucleotide diversity and divergence patterns using linear regression analyses.

## Supporting information

S1 Text Description of additional results. (DOCX)

S1 Fig Phenotypic parallelism. (**A**) Effect sizes (partial η^2^) of linear model terms for each phenotypic trait and PC1 to PC4 of the linear trait principal component analysis. (**B**) Principal component plots for PC2 vs PC3 and PC3 vs PC4, with points showing the centroids and standard error for each ecotype and sympatric ecotype pairs are connected by lines. Points are coloured by ecotype: blue–planktivorous, orange–benthivorous, green–piscivorous, and red–insectivorous. (**C**) Distribution of phenotypic trajectory angles and differences in phenotypic trajectory lengths for comparisons between replicated ecotype-pairs (N = 24) and between non-replicated ecotype pairs (N = 30). The mean for each dataset is shown by the solid lines and the p-value shows the result of a Wilcoxon rank sum test testing the difference in the mean between both datasets.
(TIFF)

S2 Fig Principal component plots by lineage. (**A**) Principal component plots based on 6,039 SNPs showing PC1 to PC14 for individuals from the Atlantic lineage (N = 300 ind.). (**B**) Principal component plots for all individuals from the Siberian lineage (N = 328 ind.) based on 4,475 SNPs. Individual points are shaped based on ecotype and coloured by lake.
(TIFF)

S3 Fig Splitstree network. Phylogenetic Splitstree network for all individuals (N = 630) from the Atlantic and Siberian lineage.
(TIFF)

S4 Fig Haplotype network for the mitochondrial *ND1* gene (N = 107). The size of each circle corresponds to the number of individuals sharing a haplotype. When sympatric ecotypes share one or several haplotypes than the circles or pies are only coloured by lake of origin. However, when sympatric ecotypes have distinct haplotypes, then each circle or pie is coloured by ecotype.
(TIFF)

S5 Fig Treemix maximum-likelihood trees. (**A**) Likelihood of trees with different numbers of fitted migration events for the Atlantic and Siberian lineage. (**B**) ML-trees from *Treemix* with zero and four migration edges respectively for all populations from the Atlantic lineage. Migration edges are shown as arrows coloured by migration weight. (**C**) *Treemix* ML-trees for all populations from the Siberian lineage with zero and six migration events fitted. Population codes are described in S1 Table.
(TIFF)

S6 Fig Abba-baba results. (**A**) Heatmaps showing the D-statistic for each comparison in the Atlantic lineage using either the maximum-likelihood tree to define sister pairs (left), the BBAA pattern based on the derived allele determined by the outgroup (Davatchan) (middle), or the lowest possible D-statistics (Dmin). D-statistic scores and p-values are colour-coded and shown in the legend, with dark red squares representing the strongest signal of introgression. (**B**) The same is shown for the Siberian lineage, using Dughaill as an outgroup. Numerical results are shown in S5 Table.
(TIFF)

S7 Fig Continuum of pairwise genetic differentiation. Mean genome wide Fst plotted against its rank, ordered by increasing Fst. Points are coded based on comparison; grey = allopatric comparison between ecotypes from different lakes, blue = parapatric comparison between ecotypes from adjacent connected lakes from the same catchment, sympatric comparison between ecotypes from the same lake. Note that some sympatric ecotype pairs show higher degrees of genetic differentiation than ecotypes in allopatric comparisons.
(TIFF)

S8 Fig Overview of all tested demographic models in *fastsimcoal2*. (**A**) Illustrations of two-population models tested. (**B**) Three-population models tested for the evolutionary history of Dughaill and Uaine. (**C**) All three-population models tested for Kalarskii-Davatchan and (**D**) Kamkanda. We inferred parameters, such as divergence times, timing of secondary contact and introgression, strength of introgression and gene flow and effective population sizes.
(TIFF)

S9 Fig Illustrations of the most likely evolutionary history for each population, and inferred parameters using *fastsimcoal2*. All parameters are point estimates that were averaged across the five runs with the highest likelihood. AIC values for the best competing models per comparison are given in S7 Table.
(TIFF)

S10 Fig Genetic differentiation and selection. (**A**) Genome scan plot with top 5%-quantile Fst outlier loci highlighted in red. Chromosomes are alternatingly highlighted by blue boxes, and unplaced scaffolds are placed on the right side. (**B,C**) Genome-wide patterns of differentiation between sympatric ecotypes in the (**B**) Atlantic and (**C**) Siberian lineage. The z-transformed Fst (ZFst) is plotted against the delta nucleotide-diversity (Δπ; benthivorous–planktivorous and piscivorous–planktivorous). If the Δπ deviates from zero, it shows genetic diversity at this locus is reduced in one of the ecotypes. Loci with ZFst values above 4 were inferred to be significantly differentiated (red dots) and loci with ZFst above 3 (blue dots) are also reported. (**D**) Manhattan plots showing the hierarchical bayescan results (-log10[q-value]) for the Siberian benthivorous-planktivorous ecotype pairs across the Arctic charr genome. None of the SNPs show significant signatures of parallel selection across ecotype pairs (FDR < 0.1, red dashed line).
(TIFF)

S11 Fig Outlier analyses based on permutated Fst null-distributions. (**A**) Examples of comparisons between empirical Fst distributions between sympatric ecotypes and permutated Fst null-distributions. Examples are shown for four ecotype pairs with different evolutionary histories and differences in effective population sizes between ecotypes (S7 Table, S9 Fig). Mode of speciation (e.g. divergence time), rather than differences in effective population size, seem to affect the Fst null distribution and sensitivity for detecting Fst outlier loci. Dotted lines show the 95^th^ percentile of the empirical (red) and permutated null-distribution (grey). (**B**) Number of shared Fst outlier loci (loci with empiricial Fst above the permutated 95^th^ percentile) for benthivorous-plantivorous (left) and planktivorous-piscivorous (right) ecotype pairs. Significant comparisons are highlighted by asterisks.
(TIFF)

S12 Fig LD decay. (**A**) Left: LD decay plots showing the rate of decay in LD [r^2^] in all pairwise SNP comparisons for the four bimodal Scottish lakes. Right: LD decay with increasing distance from Fst outlier SNPs in the Atlantic lineage. (**B**) Left: LD decay in all pairwise SNP comparisons in the Siberian lineaege. The dataset is split into four plots to make the plots legible. Right: LD decay with increasing distance from Fst outlier SNPs in the Siberian lineage. LD decay could not be estimated for outlier SNPs in Tokko and Kudushkit due to the low number of SNPs.
(TIFF)

S13 Fig Significance of ecotype-association in redundancy analyses. (**A**) Distribution of z-scores along RDA1 across all SNPs estimated using a redundancy analysis for the Atlantic lineage. SNPs with z-scores above and below 2 or -2 (dashed lines) are considered significantly associated with ecotype across lakes. (**B**) Distribution of z-scores along RDA1 (benthivorous-planktivorous divergence; orange) and RDA2 (piscivorous divergence, green) across all SNPs.
(TIFF)

S14 Fig Gene expression results. (**A**) Principal component (PCA) plots based on gene expression data for PC3 vs PC4 and PC5 vs PC6. Individuals are shown by individual points shaped by ecotype and coloured by lake of origin. Centroids for each ecotype are shown including standard error and coloured by ecotype (blue–planktivorous, orange–benthivorous, green–piscivorous, red–insectivorous). Centroids of sympatric ecotypes are connected by a line. (**B**) Linear model term effect sizes (partial η^2^) for PC1 to PC4 from the gene expression PCA. (**C**) Boxplots showing the normalised expression of different haemoglobin paralogs across ecotypes and lakes. (**D**) Expression of two genes (*ABCC8* and *ALDOA*) that are significantly differentially expressed in 5 out of 7 ecotype pair comparisons. The boxplots show the normalized expression for each ecotype by lake (lakes highlighted by alternating shaded areas) and ecotypes are colour coded. (**E**) Distribution of explained variances for each transcript by model term for the linear mixed-effects model of gene expression. (**F**) Correlation between expression of WGCNA module eigengenes and lake (population of origin) and ecotype. Each row represents a module of co-expressed genes (identified by colour). Spearman’s correlation coefficients for the module expression-variable correlations are given in each cell. The corresponding corrected p-values are given in parenthesis. Significant correlations are highlighted in bold. Cells are coloured based on their correlation, with orange cells being correlated with up-regulation of gene expression in benthivorous ecotypes and blue being associated with up-regulation in planktivorous ecotypes. (**G**) Distribution of gene expression trajectory angles and differences in trajectory lengths for within and between ecotype comparisons. Distributions are coloured by comparisons between replicated ecotype-pairs (red; N = 6) and between non-replicated ecotype pairs (blue; N = 9). The mean for each dataset is shown by the solid lines. Means do not differ between the comparisons (P > 0.05).
(TIFF)

S15 Fig Correlation analyses. (**A**) Comparison of neutral genetic differentiation between sympatric ecotypes diverged post-LGM under ongoing gene flow or pre-LGM with secondary contact. P-value for Wilcoxon-test is shown in the plot. (**B,C**) Distribution of (**B**) neutral allele frequency trajectory angles and differences in trajectory lengths and (**C**) adaptive allele frequency trajectory angles and lengths for within and between ecotype comparisons. Comparisons are coloured by comparisons between replicated ecotype-pairs (red) and between non-replicated ecotype pairs (blue). The mean for each dataset is shown by the solid lines and the P-value for Wilcoxon-tests is shown in the plot. (**D**) Correlation between mean trait variance across all sympatric ecotypes and ecosystem size (PC1) across all populations. Larger ecosystems harbor populations with a larger mean trait variance (results of linear model shown in plot).
(TIFF)

S1 Table Sampling sites and sample sizes for populations used for phenotypic and genomic analysis of Arctic charr from the Atlantic and Siberian lineage. (DOCX)

S2 Table Results of phenotypic trajectory analysis based on all seven linear traits. Differences in trajectory lengths (ΔL, upper part) or angles (*θ*, lower part) below the diagonal and p-values are above the diagonal.
(DOCX)

S3 Table Effect sizes (partial η^2^) for each model term from trait-by-trait linear models. (DOCX)

S4 Table Selected results for the *f4*- and *f3*-statistics for both lineages. (DOCX)

S5 Table Results for D-statistics (all comparisons) in the Atlantic and Siberian lineage. (XLSX)

S6 Table Results of Fst genome scans and associated analyses between sympatric ecotypes. (DOCX)

S7 Table The most likely demographic models for each lake population. Shown are the four best fitting models for each population/comparison and the respective ΔAIC between them.
(DOCX)

S8 Table Genes containing ecotype-associated SNPs identified in the cRDA analysis in both lineages. (DOCX)

S9 Table Gene ontology overrepresentation results for ecotype-associated expressed genes (RDA). (DOCX)

S10 Table WGCNA networks. Gene co-expression modules in network generated from 1,512 ecotype associated genes.
(DOCX)

S11 Table Cis-regulated ecotype-associated gene expression. Ecotype-associated expressed genes that are associated with *cis*-eQTL.
(DOCX)

## References

[1] Conway Morris S. Life’s solution: inevitable humans in a lonely universe. Cambridge University Press; 2003.

[2] Gould S. Wonderful life: the Burgess Shale and the nature of history. Wonderful life Burgess Shale Nat Hist. 1990.

[3] Elmer KR, Meyer A. Adaptation in the age of ecological genomics: Insights from parallelism and convergence. Trends Ecol Evol. 2011;26: 298–306. 10.1016/j.tree.2011.02.008 21459472

[4] Kaeuffer R, Peichel CL, Bolnick DI, Hendry AP. Parallel and nonparallel aspects of ecological, phenotypic, and genetic divergence across replicate population pairs of lake and stream stickleback. Evolution. 2012;66: 402–418. 10.1111/j.1558-5646.2011.01440.x 22276537 PMC4499166

[5] Mahler DL, Ingram T, Revell LJ, Losos JB. Exceptional Convergence on the Macroevolutionary Landscape in Island Lizard Radiations. Science (80-). 2013;341: 292–295. 10.1126/science.1232392 23869019

[6] Kowalko JE, Rohner N, Linden TA, Rompani SB, Warren WC, Borowsky R, et al Convergence in feeding posture occurs through different genetic loci in independently evolved cave populations of Astyanax mexicanus. Proc Natl Acad Sci. 2013;110: 16933–16938. 10.1073/pnas.1317192110 24085851 PMC3801050

[7] Elmer KR, Fan S, Kusche H, Luise Spreitzer M, Kautt AF, Franchini P, et al Parallel evolution of Nicaraguan crater lake cichlid fishes via non-parallel routes. Nat Commun. 2014;5: 5168 10.1038/ncomms6168 25346277

[8] Schluter D. Ecology and the origin of species. Trends Ecol Evol. 2001;16: 372–380. 10.1016/s0169-5347(01)02198-x 11403870

[9] Endler JA. Natural selection in the wild. Princeton University Press 1986.

[10] Oke KB, Rolshausen G, LeBlond C, Hendry AP. How Parallel Is Parallel Evolution? A Comparative Analysis in Fishes. Am Nat. 2017;190: 1–16. 10.1086/691989 28617637

[11] Bolnick DI, Barrett RDH, Oke KB, Rennison DJ, Stuart YE. (Non)Parallel Evolution. Annu Rev Ecol Evol Syst. 2018;12: 303–330.

[12] Stuart YE, Veen T, Weber JN, Hanson D, Ravinet M, Lohman BK, et al Contrasting effects of environment and genetics generate a continuum of parallel evolution. Nat Ecol Evol. 2017;1: 158 10.1038/s41559-017-0158 28812631

[13] Conte GL, Arnegard ME, Peichel CL, Schluter D. The probability of genetic parallelism and convergence in natural populations. Proc Biol Sci. 2012;279: 5039–47. 10.1098/rspb.2012.2146 23075840 PMC3497250

[14] Nosil P, Villoutreix R, de Carvalho CF, Farkas TE, Soria-Carrasco V, Feder JL, et al Natural selection and the predictability of evolution inTimemastick insects. Science. 2018;359: 765–770. 10.1126/science.aap9125 29449486

[15] Langerhans RB. Predictability and Parallelism of Multitrait Adaptation. J Hered. 2017;109: 59–70. 10.1093/jhered/esx043 28482006

[16] Collyer ML, Adams DC. Phenotypic trajectory analysis: comparison of shape change patterns in evolution and ecology. Hysterix, Ital J Mammal. 2013;24: 75–83.

[17] Langerhans BR, DeWitt TJ. Shared and unique features of evolutionary diversification. Am Nat. 2004;164: 335–349. 10.1086/422857 15478089

[18] Soria-Carrasco V, Gompert Z, Comeault A a, Farkas TE, Parchman TL, Johnston JS, et al Stick insect genomes reveal natural selection’s role in parallel speciation. Science. 2014;344: 738–42. 10.1126/science.1252136 24833390

[19] Linnen CR, Kingsley EP, Jensen JD, Hoekstra HE. On the Origin and Spread of an Adaptive Allele in Deer Mice. Science (80-). 2009;325: 1095–1098. 10.1126/science.1175826 19713521 PMC2736094

[20] Filteau M, Pavey S a, St-Cyr J, Bernatchez L. Gene coexpression networks reveal key drivers of phenotypic divergence in lake whitefish. Mol Biol Evol. 2013;30: 1384–96. 10.1093/molbev/mst053 23519315

[21] Arendt J, Reznick D. Convergence and parallelism reconsidered: what have we learned about the genetics of adaptation?. Trends Ecol Evol. 2008;23: 26–32. 10.1016/j.tree.2007.09.011 18022278

[22] Zhao L, Wit J, Svetec N, Begun DJ. Parallel Gene Expression Differences between Low and High Latitude Populations of Drosophila melanogaster and D. simulans. 2015; 1–25. 10.1371/journal.pgen.1005184 25950438 PMC4423912

[23] McGirr JA, Martin CH. Parallel evolution of gene expression between trophic specialists despite divergent genotypes and morphologies. Evol Lett. 2018; 62–75. 10.1002/evl3.41 30283665 PMC6089502

[24] Elmer KR, Meyer A. Adaptation in the age of ecological genomics: insights from parallelism and convergence. Trends Ecol Evol. 2011;26: 298–306. 10.1016/j.tree.2011.02.008 21459472

[25] Schluter D. Ecological Speciation in Postglacial Fishes. Philos Trans R Soc B Biol Sci. 1996;351: 807–814. 10.1098/rstb.1996.0075

[26] Jonsson B, Jonsson N. Polymorphism and speciation in Arctic charr. J Fish Biol. 2001;58: 605–638. 10.1006/jfbi.2000.1515

[27] Alekseyev SS, Samusenok VP, Matveev AN, Yu M. Diversification, sympatric speciation, and trophic polymorphism of Arctic charr, Salvelinus alpinus complex, in Transbaikalia. Environ Biol Fishes. 2002;64: 97–114.

[28] Elmer KR, Lehtonen TK, Kautt AF, Harrod C, Meyer A. Rapid sympatric ecological differentiation of crater lake cichlid fishes within historic times. BMC Biol. 2010;8: 60 10.1186/1741-7007-8-60 20459869 PMC2880021

[29] Elmer KR, Fan S, Kusche H, Spreitzer ML, Kautt AF, Franchini P, et al Parallel evolution of Nicaraguan crater lake cichlid fishes via non-parallel routes. Nat Commun. 2014;5: 1–8. 10.1038/ncomms6168 25346277

[30] Bernatchez L, Renaut S, Whiteley AR, Derome N, Jeukens J, Landry L, et al On the origin of species: insights from the ecological genomics of lake whitefish. Philos Trans R Soc Lond B Biol Sci. 2010;365: 1783–1800. 10.1098/rstb.2009.0274 20439281 PMC2871888

[31] Siwertsson A, Knudsen R, Adams CE, Præbel K, Amundsen PA. Parallel and non-parallel morphological divergence among foraging specialists in European whitefish (Coregonus lavaretus). Ecol Evol. 2013;3: 1590–1602. 10.1002/ece3.562 23789070 PMC3686194

[32] Saltykova E, Siwertsson A, Knudsen R. Parallel phenotypic evolution of skull-bone structures and head measurements of Arctic charr morphs in two subarctic lakes. Environ Biol Fishes. 2017;100: 137–148. 10.1007/s10641-016-0564-z

[33] Adams CE, Huntingford FA. Inherited differences in head allometry in polymorphic Arctic charr from Loch Rannoch, Scotland. J Fish Biol. 2002;60: 515–520. 10.1006/jfbi.2002.1867

[34] Garduño-Paz M V., Adams CE, Verspoor E, Knox D, Harrod C. Convergent evolutionary processes driven by foraging opportunity in two sympatric morph pairs of Arctic charr with contrasting post-glacial origins. Biol J Linn Soc. 2012;106: 794–806. 10.1111/j.1095-8312.2012.01906.x

[35] Hooker OE, Barry J, Van Leeuwen TE, Lyle A, Newton J, Cunningham P, et al Morphological, ecological and behavioural differentiation of sympatric profundal and pelagic Arctic charr (Salvelinus alpinus) in Loch Dughaill Scotland. Hydrobiologia. 2016;783: 209–221. 10.1007/s10750-015-2599-0

[36] Lecaudey LA, Schliewen UK, Osinov AG, Taylor EB, Bernatchez L, Weiss SJ. Inferring phylogenetic structure, hybridization and divergence times within Salmoninae (Teleostei: Salmonidae) using RAD-sequencing. Mol Phylogenet Evol. 2018;124: 82–99. 10.1016/j.ympev.2018.02.022 29477383

[37] Adams DC, Collyer ML. A general framework for the analysis of phenotypic trajectories in evolutionary studies. Evolution (N Y). 2009;63: 1143–1154. 10.1111/j.1558-5646.2009.00649.x 19210539

[38] Kusche H, Elmer KR, Meyer A. Sympatric ecological divergence associated with a color polymorphism. BMC Biol. 2015;13: 82 10.1186/s12915-015-0192-7 26437665 PMC4594650

[39] Klemetsen A, Elliott J, Knudsen R, Sørensen P. Evidence for genetic dfferences in the offspring of two sympatric morphs of Arctic charr. J Fish Biol. 2002;60: 933–950. 10.1006/jfbi.2002.1905

[40] Gordeeva N V, Alekseyev SS, Matveev AN, Samusenok VP. Parallel evolutionary divergence in Arctic charr Salvelinus alpinus (L.) complex from Transbaikalia: variation in differentiation degree and segregation of genetic diversity between sympatric forms. Can J Fish Aquat Sci. 2015;72: 96–115. 10.1139/cjfas-2014-0014

[41] Wilson a J, Gíslason D, Skúlason S, Snorrason SS, Adams CE, Alexander G, et al Population genetic structure of Arctic charr, Salvelinus alpinus, from northwest Europe on large and small spatial scales. Mol Ecol. 2004;13: 1129–42. 10.1111/j.1365-294X.2004.02149.x 15078451

[42] Alekseyev SS, Bajno R, Gordeeva N V., Reist JD, Power M, Kirillov a. F, et al Phylogeography and sympatric differentiation of the Arctic charr Salvelinus alpinus (L.) complex in Siberia as revealed by mtDNA sequence analysis. J Fish Biol. 2009;75: 368–392. 10.1111/j.1095-8649.2009.02331.x 20738544

[43] Excoffier L, Dupanloup I, Huerta-Sánchez E, Sousa VC, Foll M. Robust Demographic Inference from Genomic and SNP Data. PLoS Genet. 2013;9 10.1371/journal.pgen.1003905 24204310 PMC3812088

[44] Coyne JA, Orr HA. Speciation. Sinauer Associates Sunderland, MA; 2004.

[45] Roesti M, Gavrilets S, Hendry AP, Salzburger W, Berner D. The genomic signature of parallel adaptation from shared genetic variation. Mol Ecol. 2014; 3944–3956. 10.1111/mec.12720 24635356 PMC4122612

[46] Adams CE, Huntingford FA. Incipient speciation driven by phenotypic plasticity? Evidence from sympatric populations of Arctic charr. Biol J Linn Soc. 2004;81: 611–618.

[47] Ronkainen PHA, Pöllänen E, Törmäkangas T, Tiainen K, Koskenvuo M, Kaprio J, et al Catechol-O-methyltransferase gene polymorphism is associated with skeletal muscle properties in older women alone and together with physical activity. PLoS One. 2008;3 10.1371/journal.pone.0001819 18350156 PMC2265555

[48] Recknagel H, Elmer KR, Meyer A. Crater lake habitat predicts morphological diversity in adaptive radiations of cichlid fishes. Evolution (N Y). 2014;68: 2145–2155. 10.1111/evo.12412 24660780

[49] Recknagel H, Hooker OE, Adams CE, Elmer KR. Ecosystem size predicts eco-morphological variability in a postglacial diversification. Ecol Evol. 2017;7: 5560–5570. 10.1002/ece3.3013 28811875 PMC5552947

[50] Kautt AF, Machado-schiaffino G, Meyer A. Lessons from a natural experiment: Allopatric morphological divergence and sympatric diversification in the Midas cichlid species complex are largely influenced by ecology in a deterministic way. Evol Lett. 2018;2: 1–18. 10.1002/evl3.64 30283685 PMC6121794

[51] Skoglund S, Siwertsson A, Amundsen PA, Knudsen R. Morphological divergence between three Arctic charr morphs—the significance of the deep-water environment. Ecol Evol. 2015;5: 3114–3129. 10.1002/ece3.1573 26357540 PMC4559054

[52] Magalhaes IS, Whiting JR, D’Agostino D, Hohenlohe PA, Mahmud M, Bell MA, et al. Intercontinental genomic parallelism in multiple adaptive radiations. bioRxiv. 2019. 10.1101/856344. PMC7858233 33257817

[53] Doenz CJ, Krähenbühl AK, Walker J, Seehausen O, Brodersen J. Ecological opportunity shapes a large Arctic charr species radiation. Proc R Soc B Biol Sci. 2019;286 10.1098/rspb.2019.1992 31640512 PMC6834057

[54] Paccard A, Hanson D, Stuart YE, n Hippel FA, Kalbe M, Klepaker T, et al Repeatability of adaptive radiation depends on spatial scale: regional versus global replicates of stickleback in lake versus stream habitats. J Hered. 2019; 1–14. 10.1093/jhered/esz056 31690947

[55] Rennison DJ, Stuart YE, Bolnick DI, Peichel CL, Rennison DJ, Peichel CL. Ecological factors and morphological traits are associated with repeated genomic differentiation between lake and stream stickleback. Philos. Trans. R. Soc. B 2019;374(17777):20180241 10.1098/rstb.2018.0241 31154970 PMC6560272

[56] Yeaman S, Aeschbacher S, Bürger R. The evolution of genomic islands by increased establishment probability of linked alleles. Mol Ecol. 2016; 2542–2558. 10.1111/mec.13611 27206531

[57] Foll M, Gaggiotti OE, Daub JT, Vatsiou A, Excoffier L. Widespread signals of convergent adaptation to high altitude in Asia and America. Am J Hum Genet. 2014;95: 394–407. 10.1016/j.ajhg.2014.09.002 25262650 PMC4185124

[58] Zheng J, Payne JL, Wagner A. Cryptic genetic variation accelerates evolution by opening access to diverse adaptive peaks. Science (80-). 2019;365: 347–353. 10.1126/science.aax1837 31346060

[59] Barghi N, Tobler R, Nolte V, Jakšić AM, Mallard F, Otte KA, et al Genetic redundancy fuels polygenic adaptation in Drosophila. PLoS Biology. 2019 10.1371/journal.pbio.3000128 30716062 PMC6375663

[60] Morales HE, Faria R, Johannesson K, Larsson T, Panova M, Westram AM, et al Genomic architecture of parallel ecological divergence: beyond a single environmental contrast. Sci Adv. 2019;5: eaav996: 447854. 10.1101/447854 PMC6892616 31840052

[61] Rennison DJ, Delmore KE, Samuk K, Owens GL, Miller SE. Shared patterns of genome-wide differentiation are more strongly predicted by geography than by ecology. Am Nat. 2019 10.1086/706476 32017617

[62] Gagnaire P-A, Pavey S a, Normandeau E, Bernatchez L. The genetic architecture of reproductive isolation during speciation-with-gene-flow in lake whitefish species pairs assessed by RAD sequencing. Evolution. 2013;67: 2483–97. 10.1111/evo.12075 24033162

[63] Ravinet M, Westram A, Johannesson K, Butlin R, André C, Panova M. Shared and nonshared genomic divergence in parallel ecotypes of *Littorina saxatilis* at a local scale. Mol Ecol. 2015; 10.1111/mec.13332 26222268

[64] Roda F, Liu H, Wilkinson MJ, Walter GM, James ME, Bernal DM, et al Convergence and Divergence During the Adaptation To Similar Environments By an Australian Groundsel. Evolution (N Y). 2013;67: 2515–2529. 10.1111/evo.12136 24033164

[65] Rougeux C, Gagnaire PA, Praebel K, Seehausen O, Bernatchez L. Polygenic selection drives the evolution of convergent transcriptomic landscapes across continents within a Nearctic sister species complex. Mol Ecol. 2019;28: 4388–4403. 10.1111/mec.15226 31482603

[66] Losos JB. Improbable destinies: Fate, chance, and the future of evolution. New York, NY: Riverhead Books; 2017.

[67] Blount ZD, Lenski RE, Losos JB. Contingency and determinism in evolution: Replaying life’s tape. Science (80-). 2018;362 10.1126/science.aam5979 30409860

[68] Schneider CA, Rasband WS, Eliceiri KW. NIH Image to ImageJ: 25 years of image analysis. Nat Methods. 2012;9 10.1038/nmeth.2089 22930834 PMC5554542

[69] Adams CE, Huntingford FA. The functional significance of inherited differences in feeding morphology in a sympatric polymorphic population of Arctic charr. Evol Ecol. 2002;16: 15–25.

[70] Præbel K, Knudsen R, Siwertsson A, Karhunen M, Kahilainen KK, Ovaskainen O, et al Ecological speciation in postglacial European whitefish: Rapid adaptive radiations into the littoral, pelagic, and profundal lake habitats. Ecol Evol. 2013;3: 4970–4986. 10.1002/ece3.867 24455129 PMC3892361

[71] Nugent CM, Easton AA, Norman JD, Ferguson MM, Danzmann RG. A SNP Based Linkage Map of the Arctic Charr (Salvelinus alpinus) Genome Provides Insights into the Diploidization Process After Whole Genome Duplication. G3 Genes, Genomes, Genet. 2016;7: 543–556. 10.1534/g3.116.038026 27986793 PMC5295600

[72] Lien S, Koop BF, Sandve SR, Miller JR, Kent MP, Nome T, et al The Atlantic salmon genome provides insights into rediploidization. Nature. 2016;533: 200–205. 10.1038/nature17164 27088604 PMC8127823

[73] Tamazian G, Dobrynin P, Krasheninnikova K, Komissarov A, Koepfli K-P, O’Brien SJ. Chromosomer: a reference-based genome arrangement tool for producing draft chromosome sequences. Gigascience. 2016;5: 1–11. 10.1186/s13742-016-0141-6 27549770 PMC4994284

[74] Tang H, Zhang X, Miao C, Zhang J, Ming R, Schnable JC, et al ALLMAPS: robust scaffold ordering based on multiple maps. Genome Biol. 2015;16: 3 10.1186/s13059-014-0573-1 25583564 PMC4305236

[75] Keilwagen J, Wenk M, Erickson JL, Schattat MH, Grau J, Hartung F. Using intron position conservation for homology-based gene prediction. Nucleic Acids Res. 2016;44 10.1093/nar/gkw092 26893356 PMC4872089

[76] Simão FA, Waterhouse RM, Ioannidis P, Kriventseva EV, Zdobnov EM. BUSCO: assessing genome assembly and annotation completeness with single-copy orthologs. Bioinformatics. 2015;31: 3210–3212. 10.1093/bioinformatics/btv351 26059717

[77] Emms DM, Kelly S. OrthoFinder: solving fundamental biases in whole genome comparisons dramatically improves orthogroup inference accuracy. Genome Biol. 2015;16: 157 10.1186/s13059-015-0721-2 26243257 PMC4531804

[78] Christensen KA, Rondeau EB, Minkley DR, Leong JS, Nugent CM, Danzmann RG, et al The Arctic charr (salvelinus alpinus) genome and transcriptome assembly. PLoS One. 2018;13: 1–30. 10.1371/journal.pone.0204076 30212580 PMC6136826

[79] Recknagel H, Jacobs A, Herzyk P, Elmer KR. Double-digest RAD sequencing using Ion Proton semiconductor platform (ddRADseq-ion) with nonmodel organisms. Mol Ecol Resour. 2015;15: 1316–1329. 10.1111/1755-0998.12406 25808755

[80] Schenekar T, Lerceteau-Köhler E, Weiss S. Fine-scale phylogeographic contact zone in Austrian brown trout Salmo trutta reveals multiple waves of post-glacial colonization and a pre-dominance of natural versus anthropogenic admixture. Conserv Genet. 2014;15: 561–572. 10.1007/s10592-013-0561-0

[81] Kumar S, Stecher G, Tamura K. MEGA7: Molecular Evolutionary Genetics Analysis Version 7.0 for Bigger Datasets. Mol Biol Evol. 2016;33: 1870–1874. 10.1093/molbev/msw054 27004904 PMC8210823

[82] Leigh JW, Bryant D. popart: full‐feature software for haplotype network construction. Methods Ecol Evol. 2015;6: 1110–1116. 10.1111/2041-210x.12410

[83] Catchen JM, Amores A, Hohenlohe P, Cresko W, Postlethwait JH, De Koning D-J. Stacks: Building and Genotyping Loci De Novo From Short-Read Sequences. G3 Genes, Genomes, Genet. 2011;1: 171–182. 10.1534/g3.111.000240 22384329 PMC3276136

[84] Alexander DH, Novembre J. Fast Model-Based Estimation of Ancestry in Unrelated Individuals. Genome Res. 2009;19: 1655–1664. 10.1101/gr.094052.109 19648217 PMC2752134

[85] Meirmans PG, Tienderen PH. genotype and genodive: two programs for the analysis of genetic diversity of asexual organisms. Mol Ecol Notes. 2004;4: 792–794. 10.1111/j.1471-8286.2004.00770.x

[86] Huson DH, Bryant D. Estimating phylogenetic trees and networks using SplitsTree 4. Manuscr Prep Softw www.Split.org. 2005.

[87] Malinsky M, Trucchi E, Lawson DJ, Falush D. RADpainter and fineRADstructure: Population Inference from RADseq Data. Mol Biol Evol. 2018 10.1093/molbev/msy023 29474601 PMC5913677

[88] Pickrell JK, Pritchard JK. Inference of Population Splits and Mixtures from Genome-Wide Allele Frequency Data. PLoS Genet. 2012;8 10.1371/journal.pgen.1002967 23166502 PMC3499260

[89] Malinsky M. Dsuite—fast D-statistics and related ad- mixture evidence from VCF files. bioRxiv. 2019. 10.1101/634477. PMC7116594 33012121

[90] Gutenkunst RN, Hernandez RD, Williamson SH, Bustamante CD. Inferring the Joint Demographic History of Multiple Populations from Multidimensional SNP Frequency Data. PLoS Genet. 2009;5 10.1371/journal.pgen.1000695 19851460 PMC2760211

[91] Jacobs A, Hughes MR, Robinson PC, Adams CE, Elmer KR. The Genetic Architecture Underlying the Evolution of a Rare Piscivorous Life History Form in Brown Trout after Secondary Contact and Strong Introgression. Genes (Basel). 2018;9 10.3390/genes9060280 29857499 PMC6026935

[92] Kautt AF, Machado-Schiaffino G, Meyer A. Multispecies Outcomes of Sympatric Speciation after Admixture with the Source Population in Two Radiations of Nicaraguan Crater Lake Cichlids. PLoS Genet. 2016;12 10.1371/journal.pgen.1006157 27362536 PMC4928843

[93] Rougeux C, Bernatchez L, Gagnaire P-AA. Modeling the Multiple Facets of Speciation-with-Gene-Flow toward Inferring the Divergence History of Lake Whitefish Species Pairs (Coregonus clupeaformis). Genome Biol Evol. 2017;9: 2057–2074. 10.1093/gbe/evx150 28903535 PMC5737413

[94] Bradbury PJ, Zhang Z, Kroon DE, Casstevens TM, Ramdoss Y, Buckler ES. TASSEL: Software for association mapping of complex traits in diverse samples. Bioinformatics. 2007;23: 2633–2635. 10.1093/bioinformatics/btm308 17586829

[95] Bolger AM, Lohse M, Usadel B. Trimmomatic: a flexible trimmer for Illumina sequence data. Bioinformatics. 2014;30: 2114–2120. 10.1093/bioinformatics/btu170 24695404 PMC4103590

[96] Dobin A, Davis CA, Schlesinger F, Drenkow J, Zaleski C, Jha S, et al STAR: ultrafast universal RNA-seq aligner. Bioinformatics. 2013;29: 15–21. 10.1093/bioinformatics/bts635 23104886 PMC3530905

[97] Anders S, Pyl P, Huber W. HTSeq—a Python framework to work with high-throughput sequencing data. Bioinformatics. 2015;31: 166–169. 10.1093/bioinformatics/btu638 25260700 PMC4287950

[98] Love MI, Huber W, Anders S. Moderated estimation of fold change and dispersion for RNA-seq data with DESeq2. Genome Biol. 2014;15: 550 10.1186/s13059-014-0550-8 25516281 PMC4302049

[99] Langfelder P, Horvath S. WGCNA: an R package for weighted correlation network analysis. BMC Bioinformatics. 2008;9: 1–13. 19114008 10.1186/1471-2105-9-559 PMC2631488

[100] Shabalin AA. Matrix eQTL: ultra fast eQTL analysis via large matrix operations. Bioinformatics. 2012;28: 1353–1358. 10.1093/bioinformatics/bts163 22492648 PMC3348564

[101] Wang J, Vasaikar S, Shi Z, Greer M, Zhang B. WebGestalt 2017: a more comprehensive, powerful, flexible and interactive gene set enrichment analysis toolkit. Nucleic Acids Res. 2017;45 10.1093/nar/gkx356 28472511 PMC5570149

