# Supplementary material for: Parallelism in eco-morphology and gene expression despite variable evolutionary and genomic backgrounds in a Holarctic fish
Source: PLoS Genet. 2020 Apr 17;16(4):e1008658. doi: 10.1371/journal.pgen.1008658 (PMC7164584; doi:10.1371/journal.pgen.1008658)
Supplement: S1 Text — (DOCX) [file pgen.1008658.s001.docx]

**S1 Appendix: Expanded results.**

**Genome annotation, orthology inference and chromosome assembly.** We were able to assemble 62.6% of the draft genome (1.346 Gb) into 39 Arctic charr chromosomes with a total of 26.7% (0.573 Gb) being oriented. 37.4 % (0.804 Gb) of contigs could not be anchored to the linkage map. We annotated 44102 genes with 55443 alternative isoforms using homology-based *GeMoMa* gene prediction software. The BUSCO analysis estimated that the annotation contained 88% of completely assembled BUSCOs and 3.4% of fragmented BUSCOs, while 7.6% of BUSCOs were missing. Among the 88% completely assembled BUSCOs, 62% were duplicated, which is consistent with the estimation for the salmon genome. *Orthofinder* returned 15485 orthogroups between Arctic charr and zebrafish with a mean and median orthogroup size equal to 2.7 and 2, respectively, which can be explained by the relatively recent whole genome duplication in salmonids. We detected a total of 8428 single-copy orthogroups between Arctic charr and zebrafish.

**Analyses of secondary gene flow and introgression.**

To test for historical gene flow and introgression events across lakes that potentially led to non-independent patterns of eco-morphological divergence, we used *f-statistics* and *D-statistics* to test the role of introgression in repeated ecotype divergence in more detail (Table S4 and S5; Fig. S5 and S6).

*f-statistic:* Overall, we inferred 22 significant gene flow events that indicate complex histories of gene flow across catchments and lakes in both lineages (Fig. 4a, Fig. S5, Table S4) and that might have contributed to the complex patterns of shared ancestry. However, our results also suggest that these historical migration events do not explain the repeated divergences of ecotypes within lakes e.g. through the introgression of adaptive genetic material (43), as migration events appear random across ecotypes and lakes, and are in most comparisons not specific between replicated ecotypes across lakes (e.g. Tay-bn, Tay-pl; Awe-bn, Awe-pl, *f4*=-0.00044 ; z=-1.0814; Table S4). We did detect, though, potential signals of adaptive introgression in the piscivorous and planktivorous ecotypes of Kudushkit from e.g. Kalarskii Davatchan and Kiryalta-4, or an ancestral population (Table S4). *f3-statistics* further suggest strong introgression between ecotypes within Kalarskii Davatchan (*f3* = -0.0025, Z = -5.95; Table S5). However, signals of introgression were mostly not specific to replicated ecotypes from different lakes but rather detected across different ecotypes within and across lakes, which could either suggest more ancient introgression before ecotype divergence or artefacts due to shared co-ancestry (Fig. S6).

*Treemix:* Fitting migration events to *Treemix* maximum likelihood trees for both lineages improved the fit of the tree (Fig. S5), further suggesting that historical secondary gene flow and introgression are common across Arctic charr populations.

*D-statistic:* To test more directly for introgression we performed Abba-Baba tests (D-statistic) for all pairwise comparisons. Significant *D-statistics* indicated cases of introgression within and across several populations (Fig. S6), with strong signals of introgression between Kamkanda and Tokko (D = 0.288, P = 4.44e-16; Fig. S6b) or within the Dughaill-Uaine catchment (D = 0.292, P = <2.2e-16; Fig. S6a) (Table S5). The introgression within the Dughaill system indicated a potential secondary contact and admixture event, which is supported by significant *f3-statistics* (*f3* = -0.026, Z = -23.44; Table S5).

Although most genome-wide comparisons were not significant, this does not include the possibility of introgression of individual genes across ecotypes and lakes, though, and more detailed future investigations are needed to fully exclude the possible role of adaptive introgression in parallel ecotype convergences.

**Sharing of differentially expressed genes across ecotype pairs.**

We identified four genes (Fig S10d; *ABCC8*, *NDPK,* *ALDOA, uncharacterized protein LOC100194706*) that were consistently differentially expressed in the same direction in five out of seven ecotype pairs (Fig S10d), potentially suggesting the repeated selection on the expression of these genes. These genes are known to be associated with growth rate, body size, and metabolism, making them strong candidates for underlying ecotype divergence in Arctic charr. Additionally, 19 differentially expressed genes were shared across four ecotype pairs, which included several haemoglobin subunits (Fig. S10c).
