# Supplementary figures and images for: Parallelism in eco-morphology and gene expression despite variable evolutionary and genomic backgrounds in a Holarctic fish
Source: PLoS Genet. 2020 Apr 17;16(4):e1008658. doi: 10.1371/journal.pgen.1008658 (PMC7164584; doi:10.1371/journal.pgen.1008658)

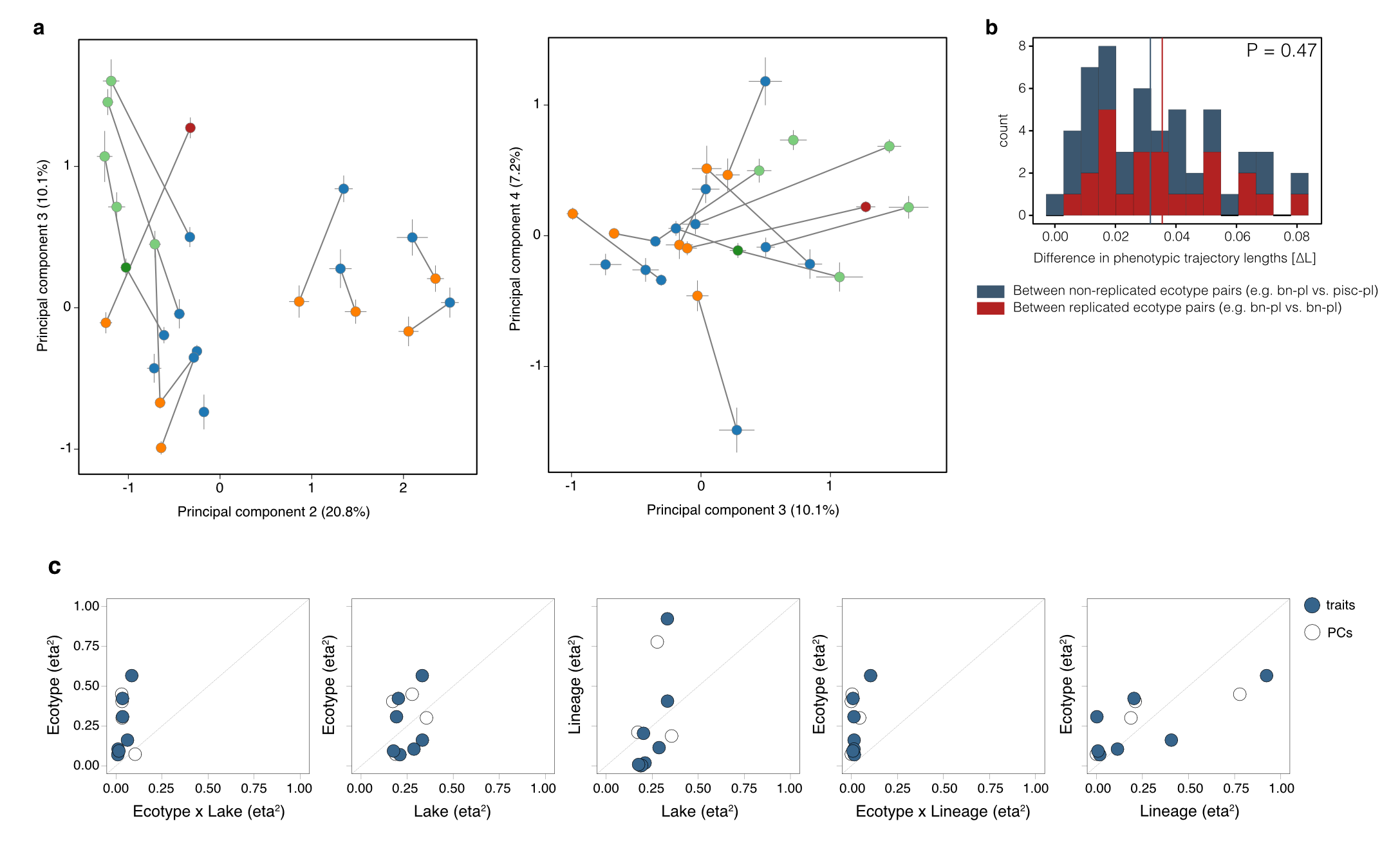

Supplement: S1 Fig — (A) Effect sizes (partial η2) of linear model terms for each phenotypic trait and PC1 to PC4 of the linear trait principal component analysis. (B) Principal component plots for PC2 vs PC3 and PC3 vs PC4, with points showing the centroids and standard error for each ecotype and sympatric ecotype pairs are connected by lines. Points are coloured by ecotype: blue–planktivorous, orange–benthivorous, green–piscivorous, and red–insectivorous. (C) Distribution of phenotypic trajectory angles and differences in phenotypic trajectory lengths for comparisons between replicated ecotype-pairs (N = 24) and between non-replicated ecotype pairs (N = 30). The mean for each dataset is shown by the solid lines and the p-value shows the result of a Wilcoxon rank sum test testing the difference in the mean between both datasets. (TIFF) [file pgen.1008658.s002.tiff]

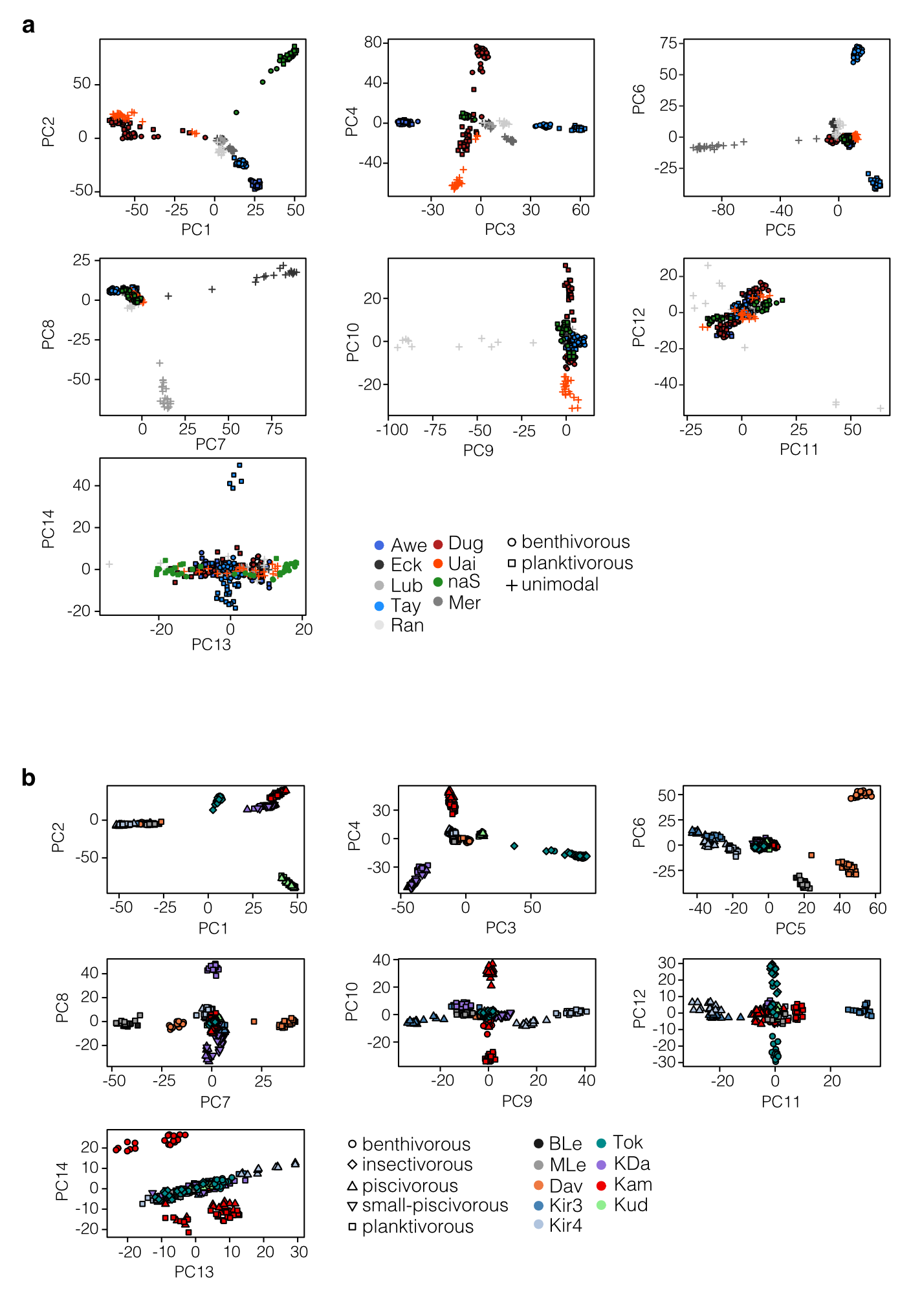

Supplement: S2 Fig — (A) Principal component plots based on 6,039 SNPs showing PC1 to PC14 for individuals from the Atlantic lineage (N = 300 ind.). (B) Principal component plots for all individuals from the Siberian lineage (N = 328 ind.) based on 4,475 SNPs. Individual points are shaped based on ecotype and coloured by lake. (TIFF) [file pgen.1008658.s003.tiff]

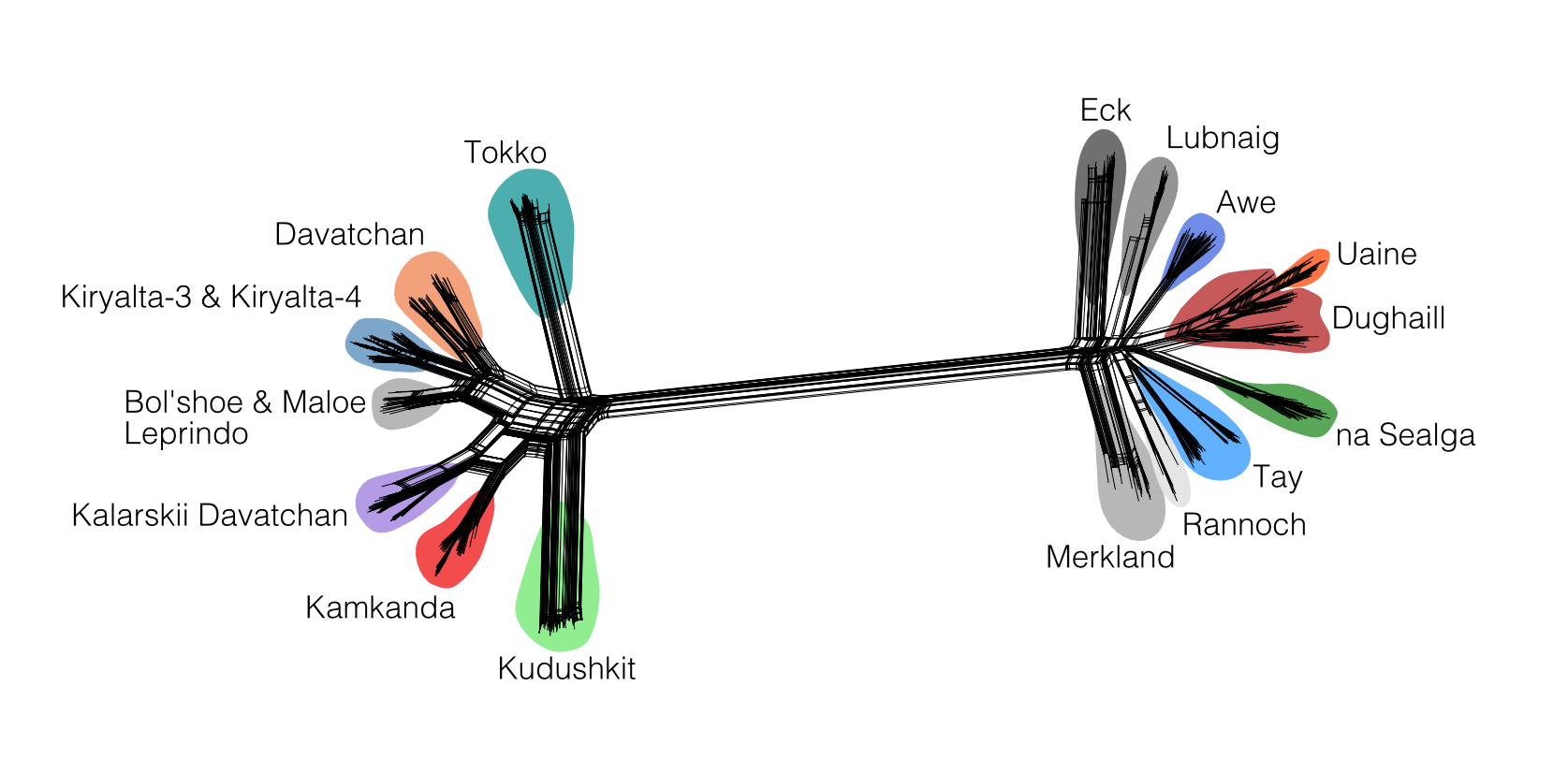

Supplement: S3 Fig — Phylogenetic Splitstree network for all individuals (N = 630) from the Atlantic and Siberian lineage. (TIFF) [file pgen.1008658.s004.tiff]

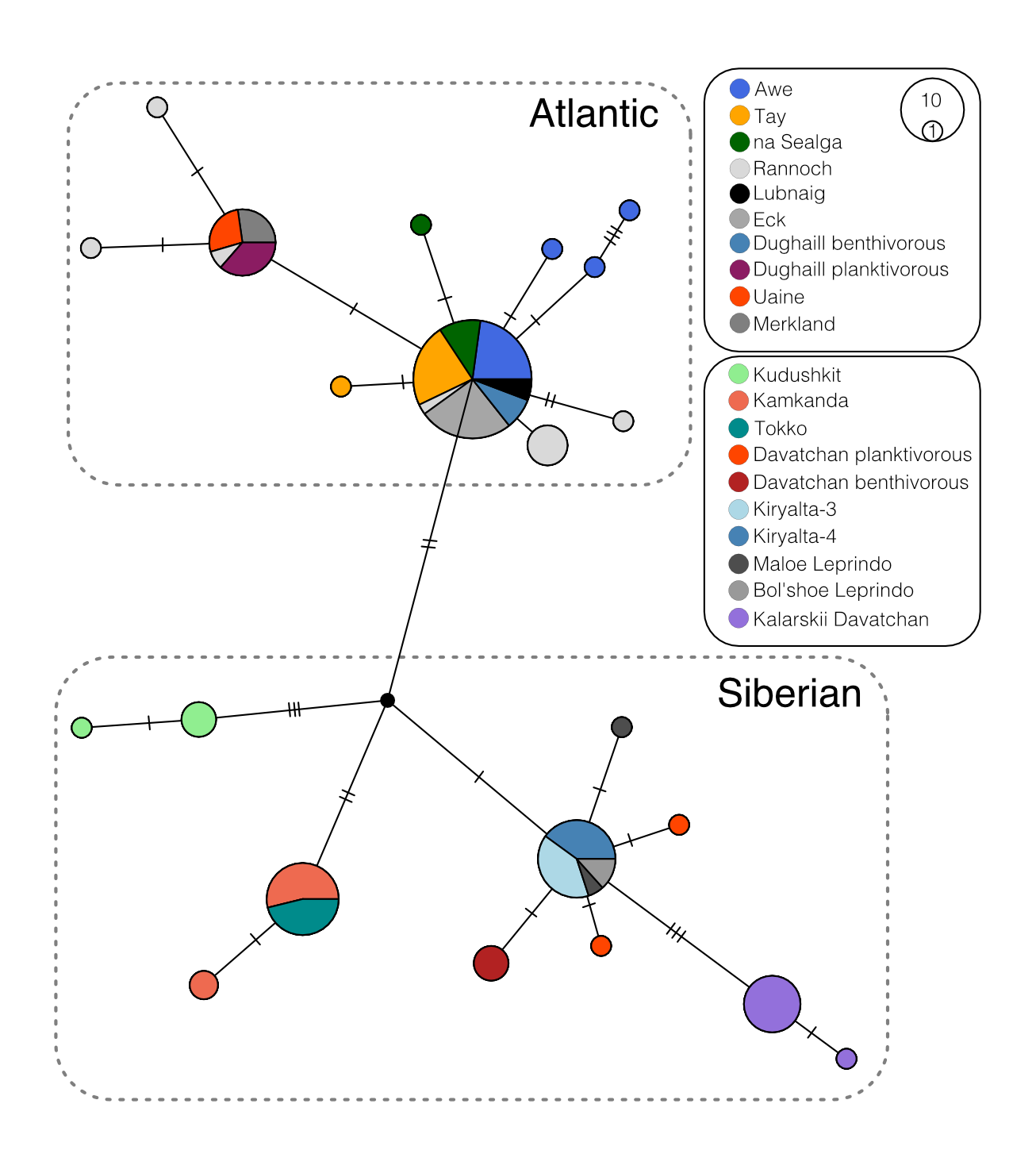

Supplement: S4 Fig — The size of each circle corresponds to the number of individuals sharing a haplotype. When sympatric ecotypes share one or several haplotypes than the circles or pies are only coloured by lake of origin. However, when sympatric ecotypes have distinct haplotypes, then each circle or pie is coloured by ecotype. (TIFF) [file pgen.1008658.s005.tiff]

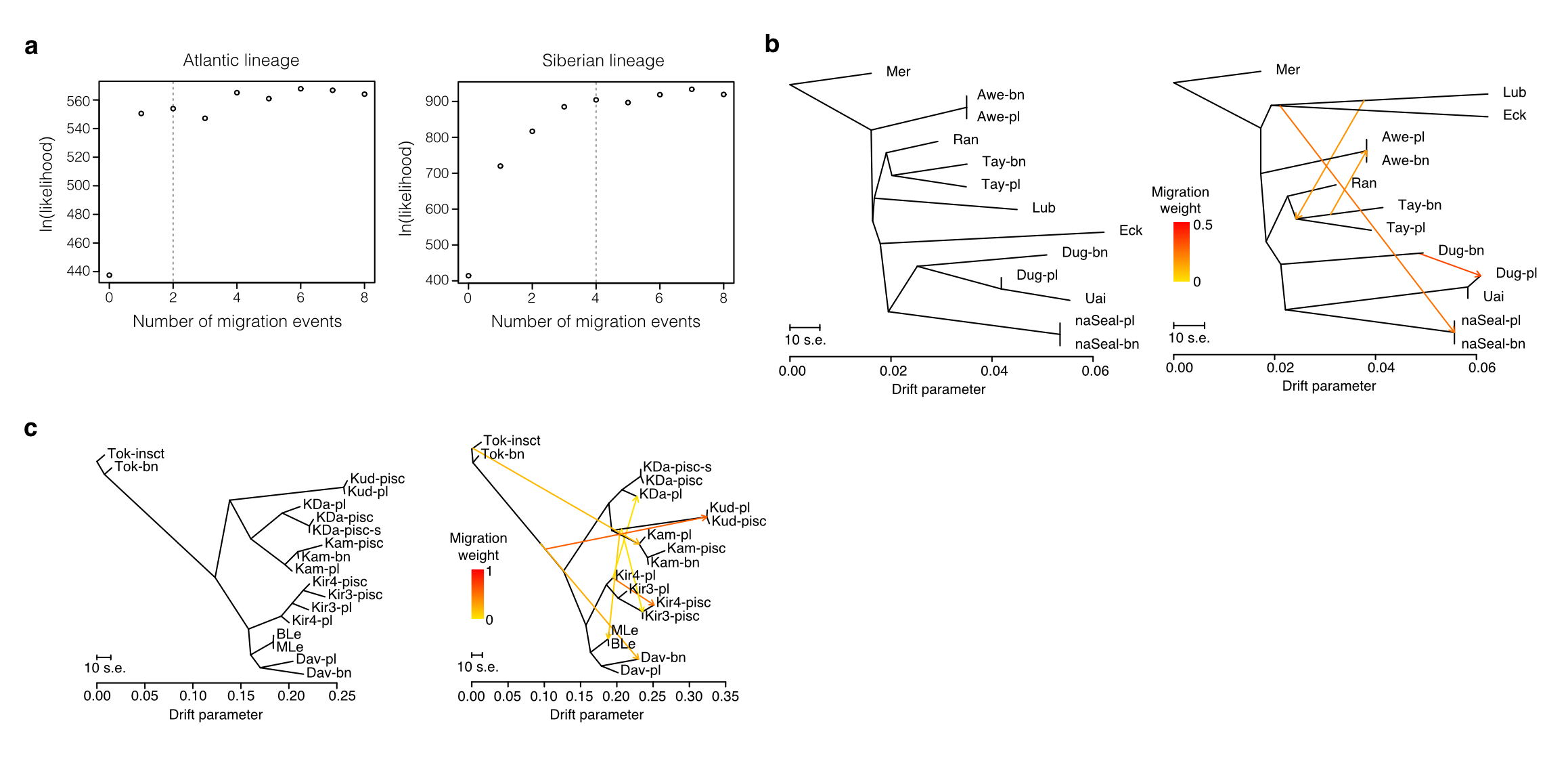

Supplement: S5 Fig — (A) Likelihood of trees with different numbers of fitted migration events for the Atlantic and Siberian lineage. (B) ML-trees from Treemix with zero and four migration edges respectively for all populations from the Atlantic lineage. Migration edges are shown as arrows coloured by migration weight. (C) Treemix ML-trees for all populations from the Siberian lineage with zero and six migration events fitted. Population codes are described in S1 Table. (TIFF) [file pgen.1008658.s006.tiff]

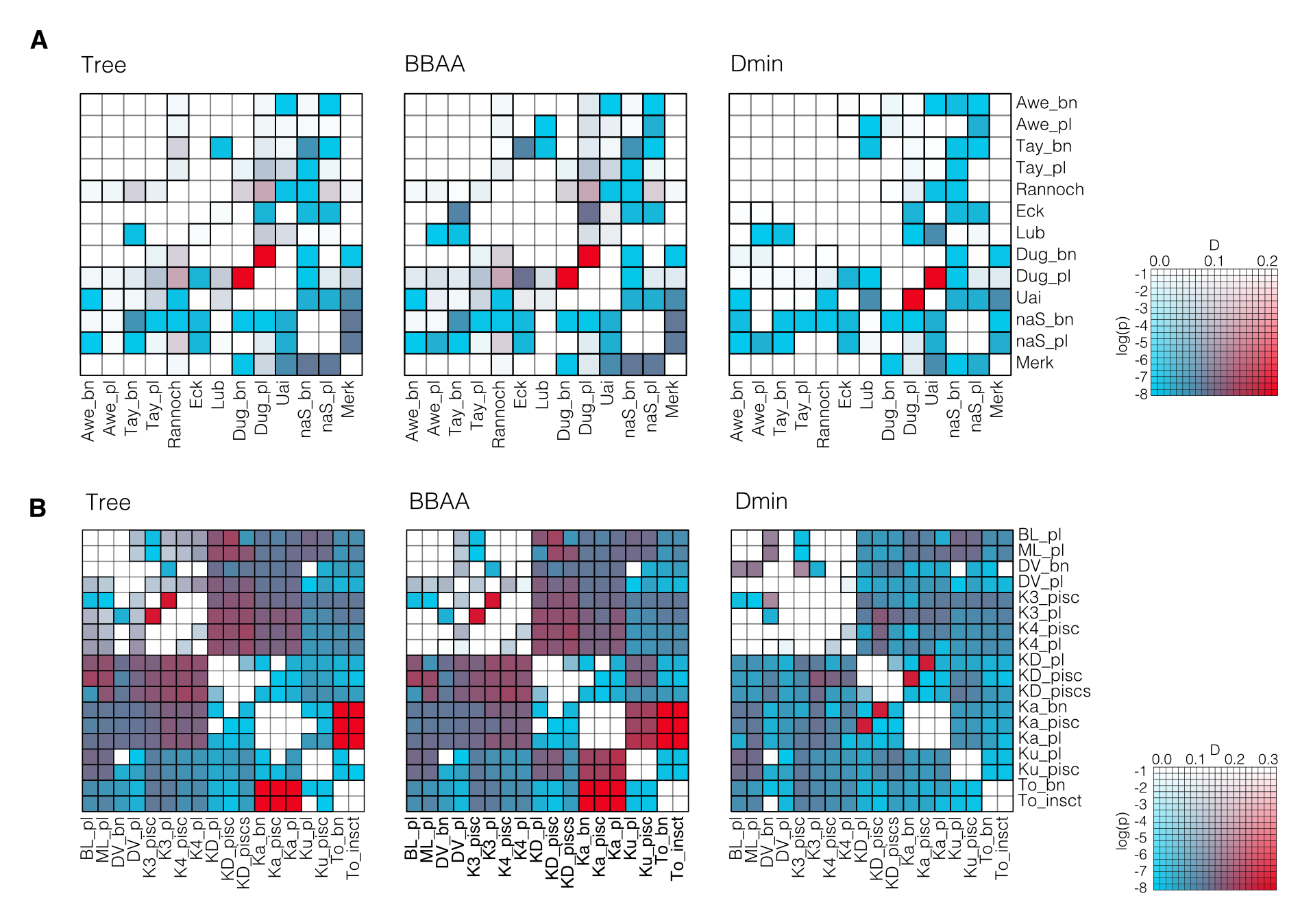

Supplement: S6 Fig — (A) Heatmaps showing the D-statistic for each comparison in the Atlantic lineage using either the maximum-likelihood tree to define sister pairs (left), the BBAA pattern based on the derived allele determined by the outgroup (Davatchan) (middle), or the lowest possible D-statistics (Dmin). D-statistic scores and p-values are colour-coded and shown in the legend, with dark red squares representing the strongest signal of introgression. (B) The same is shown for the Siberian lineage, using Dughaill as an outgroup. Numerical results are shown in S5 Table. (TIFF) [file pgen.1008658.s007.tiff]

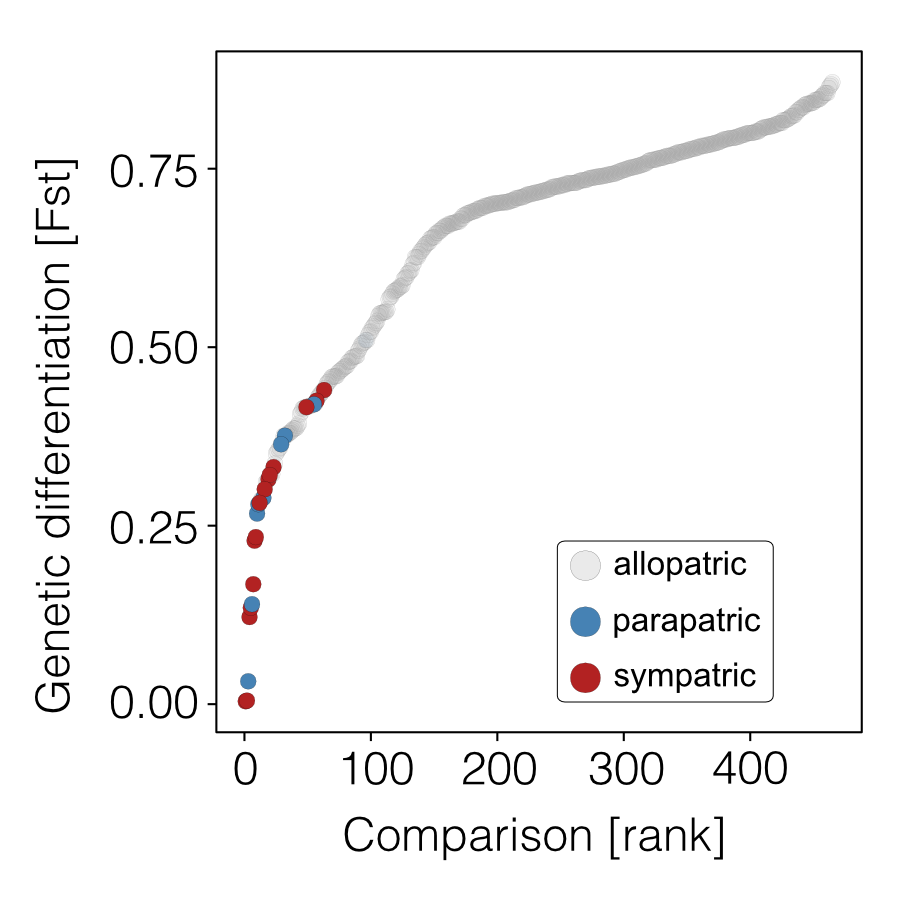

Supplement: S7 Fig — Mean genome wide Fst plotted against its rank, ordered by increasing Fst. Points are coded based on comparison; grey = allopatric comparison between ecotypes from different lakes, blue = parapatric comparison between ecotypes from adjacent connected lakes from the same catchment, sympatric comparison between ecotypes from the same lake. Note that some sympatric ecotype pairs show higher degrees of genetic differentiation than ecotypes in allopatric comparisons. (TIFF) [file pgen.1008658.s008.tiff]

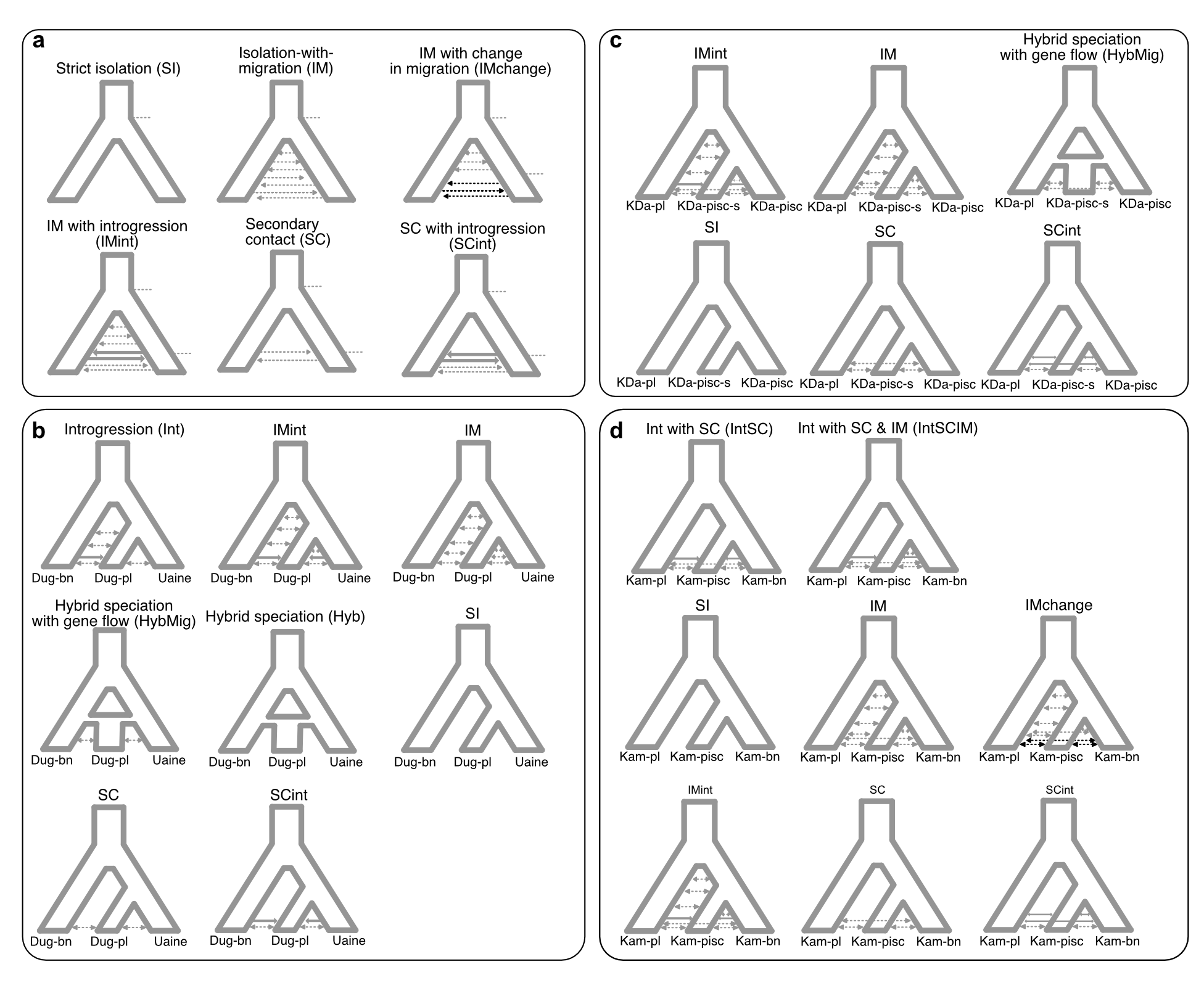

Supplement: S8 Fig — (A) Illustrations of two-population models tested. (B) Three-population models tested for the evolutionary history of Dughaill and Uaine. (C) All three-population models tested for Kalarskii-Davatchan and (D) Kamkanda. We inferred parameters, such as divergence times, timing of secondary contact and introgression, strength of introgression and gene flow and effective population sizes. (TIFF) [file pgen.1008658.s009.tiff]

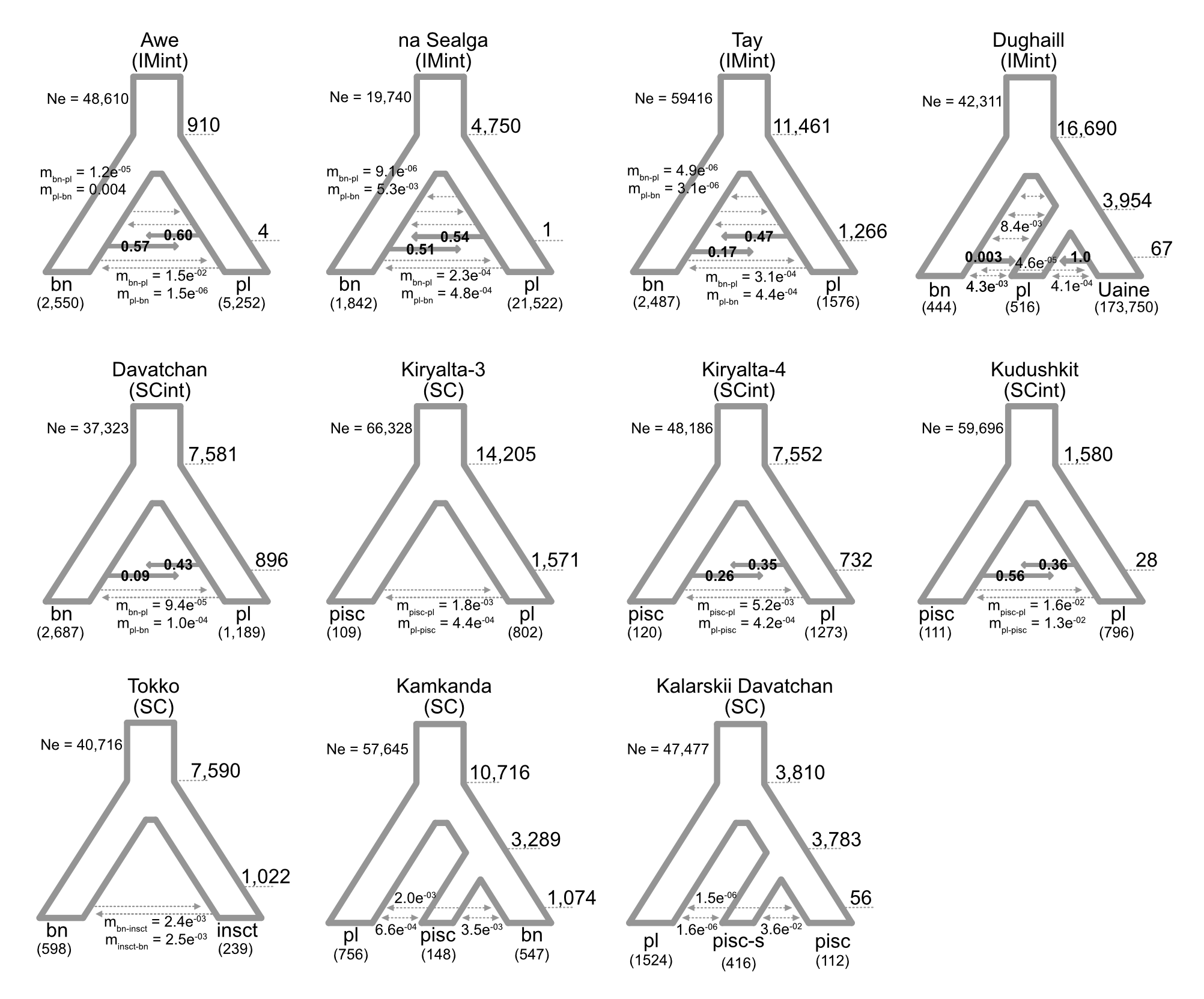

Supplement: S9 Fig — All parameters are point estimates that were averaged across the five runs with the highest likelihood. AIC values for the best competing models per comparison are given in S7 Table. (TIFF) [file pgen.1008658.s010.tiff]

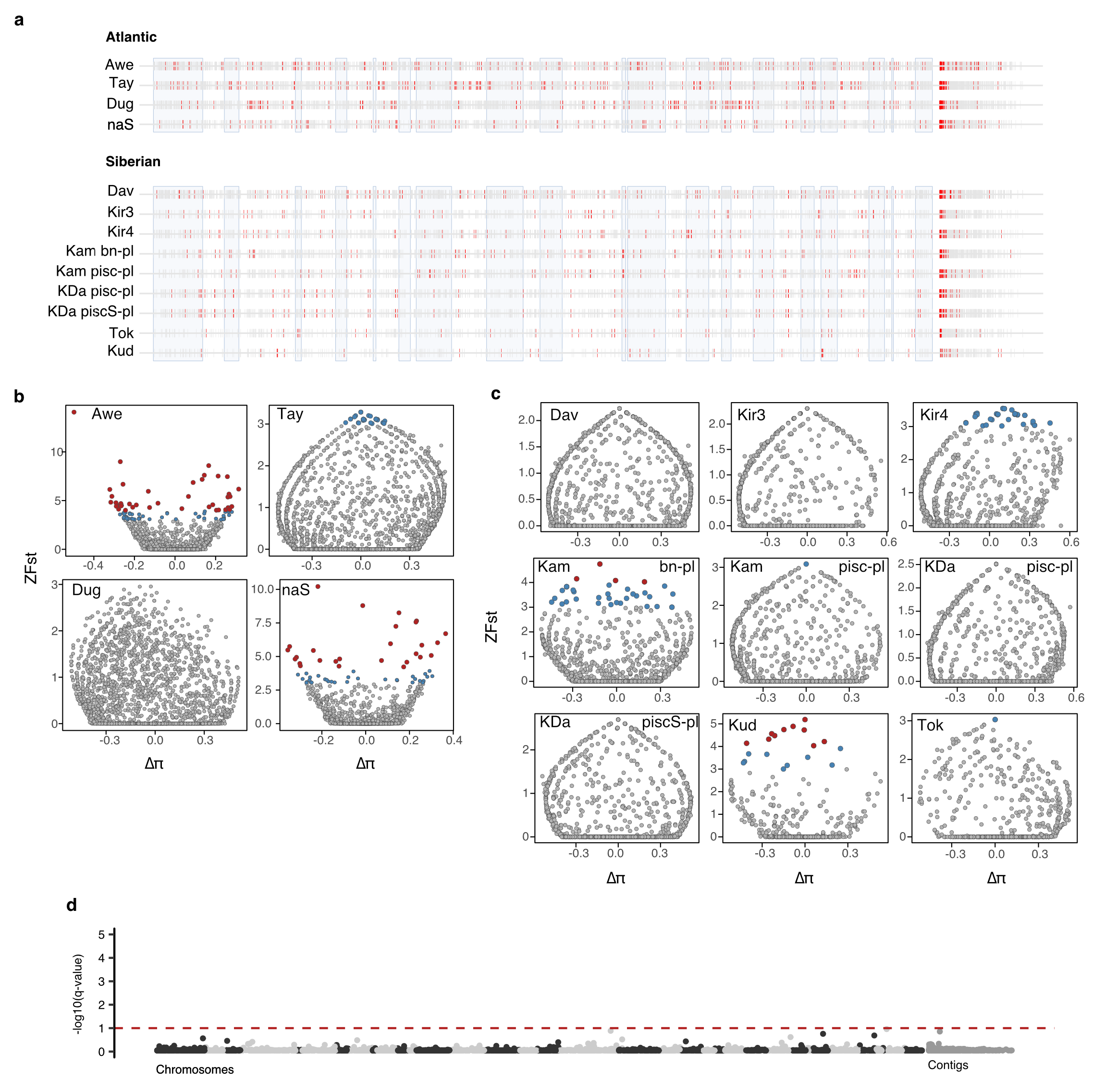

Supplement: S10 Fig — (A) Genome scan plot with top 5%-quantile Fst outlier loci highlighted in red. Chromosomes are alternatingly highlighted by blue boxes, and unplaced scaffolds are placed on the right side. (B,C) Genome-wide patterns of differentiation between sympatric ecotypes in the (B) Atlantic and (C) Siberian lineage. The z-transformed Fst (ZFst) is plotted against the delta nucleotide-diversity (Δπ; benthivorous–planktivorous and piscivorous–planktivorous). If the Δπ deviates from zero, it shows genetic diversity at this locus is reduced in one of the ecotypes. Loci with ZFst values above 4 were inferred to be significantly differentiated (red dots) and loci with ZFst above 3 (blue dots) are also reported. (D) Manhattan plots showing the hierarchical bayescan results (-log10[q-value]) for the Siberian benthivorous-planktivorous ecotype pairs across the Arctic charr genome. None of the SNPs show significant signatures of parallel selection across ecotype pairs (FDR < 0.1, red dashed line). (TIFF) [file pgen.1008658.s011.tiff]

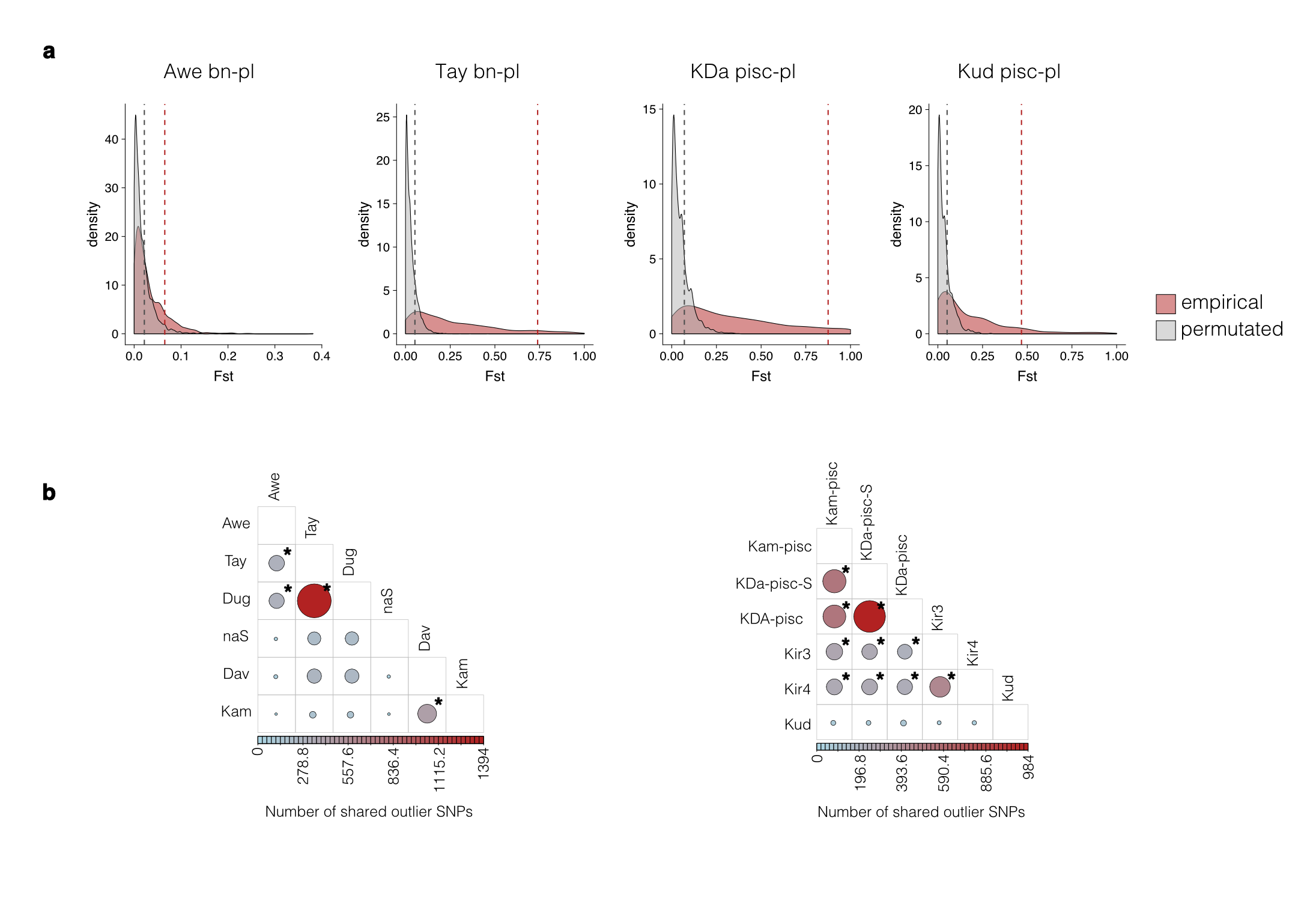

Supplement: S11 Fig — (A) Examples of comparisons between empirical Fst distributions between sympatric ecotypes and permutated Fst null-distributions. Examples are shown for four ecotype pairs with different evolutionary histories and differences in effective population sizes between ecotypes (S7 Table, S9 Fig). Mode of speciation (e.g. divergence time), rather than differences in effective population size, seem to affect the Fst null distribution and sensitivity for detecting Fst outlier loci. Dotted lines show the 95th percentile of the empirical (red) and permutated null-distribution (grey). (B) Number of shared Fst outlier loci (loci with empiricial Fst above the permutated 95th percentile) for benthivorous-plantivorous (left) and planktivorous-piscivorous (right) ecotype pairs. Significant comparisons are highlighted by asterisks. (TIFF) [file pgen.1008658.s012.tiff]

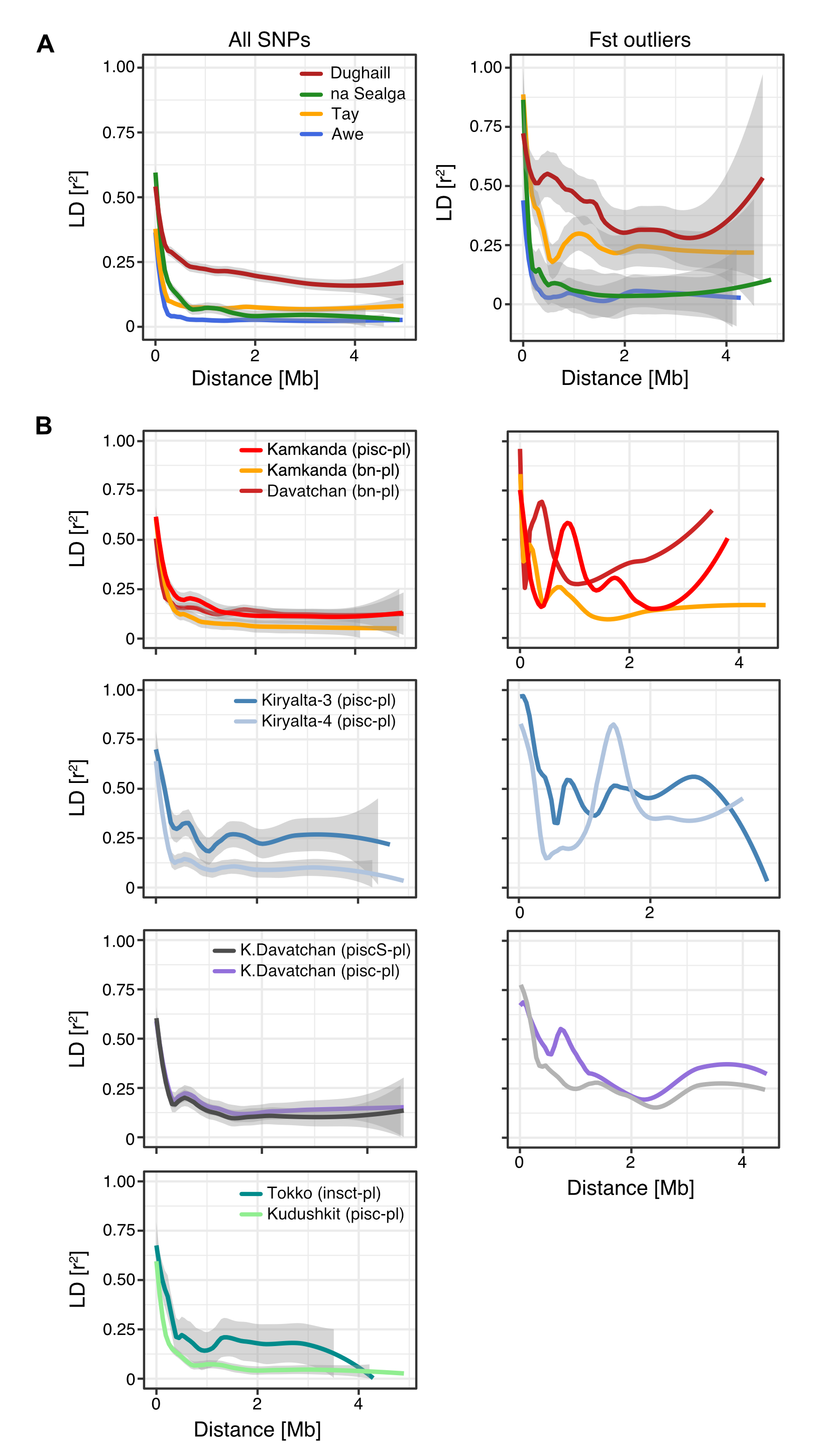

Supplement: S12 Fig — (A) Left: LD decay plots showing the rate of decay in LD [r2] in all pairwise SNP comparisons for the four bimodal Scottish lakes. Right: LD decay with increasing distance from Fst outlier SNPs in the Atlantic lineage. (B) Left: LD decay in all pairwise SNP comparisons in the Siberian lineaege. The dataset is split into four plots to make the plots legible. Right: LD decay with increasing distance from Fst outlier SNPs in the Siberian lineage. LD decay could not be estimated for outlier SNPs in Tokko and Kudushkit due to the low number of SNPs. (TIFF) [file pgen.1008658.s013.tiff]

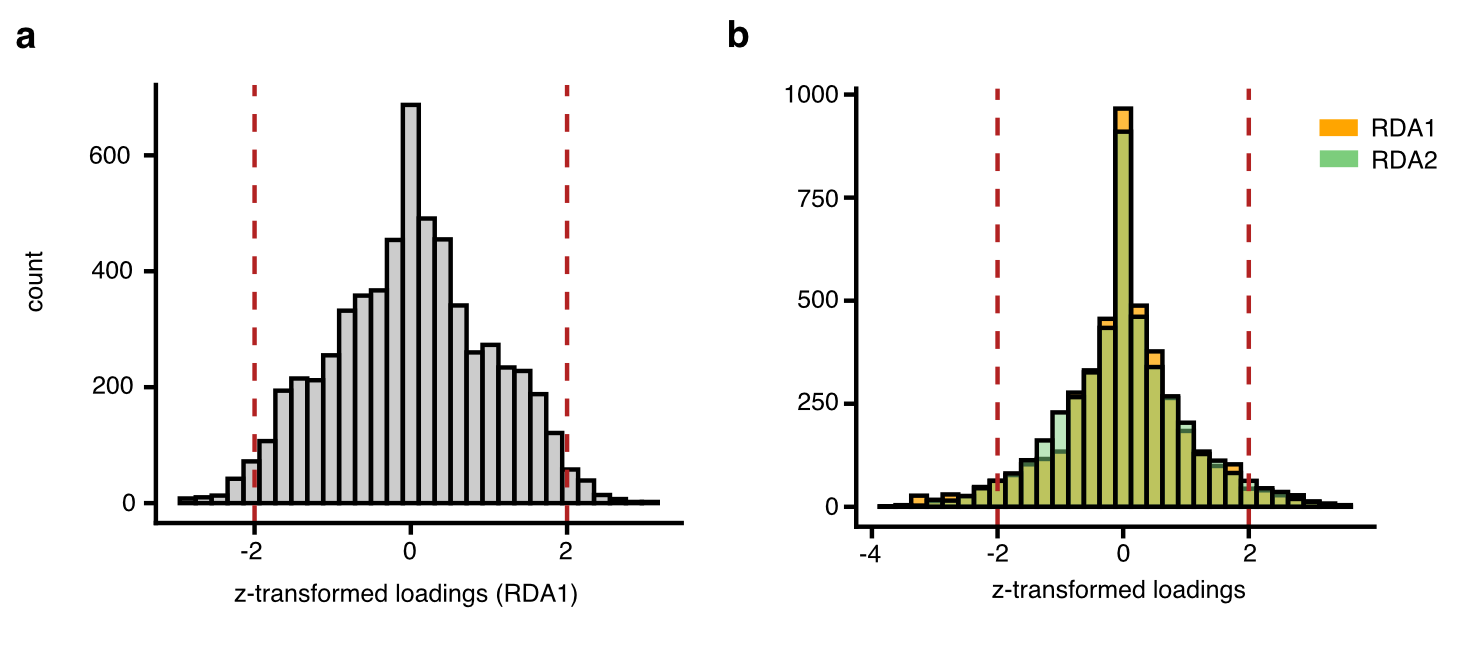

Supplement: S13 Fig — (A) Distribution of z-scores along RDA1 across all SNPs estimated using a redundancy analysis for the Atlantic lineage. SNPs with z-scores above and below 2 or -2 (dashed lines) are considered significantly associated with ecotype across lakes. (B) Distribution of z-scores along RDA1 (benthivorous-planktivorous divergence; orange) and RDA2 (piscivorous divergence, green) across all SNPs. (TIFF) [file pgen.1008658.s014.tiff]

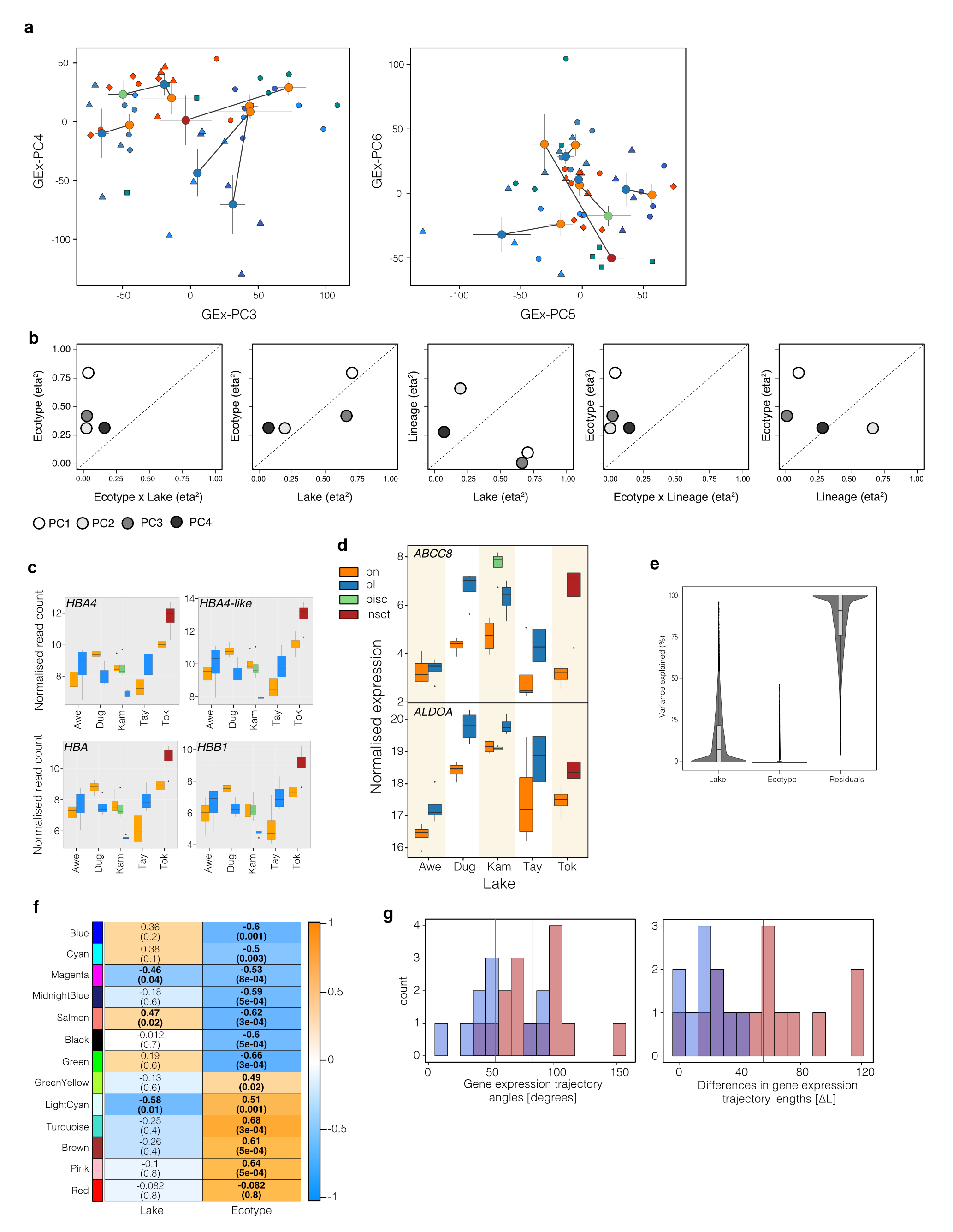

Supplement: S14 Fig — (A) Principal component (PCA) plots based on gene expression data for PC3 vs PC4 and PC5 vs PC6. Individuals are shown by individual points shaped by ecotype and coloured by lake of origin. Centroids for each ecotype are shown including standard error and coloured by ecotype (blue–planktivorous, orange–benthivorous, green–piscivorous, red–insectivorous). Centroids of sympatric ecotypes are connected by a line. (B) Linear model term effect sizes (partial η2) for PC1 to PC4 from the gene expression PCA. (C) Boxplots showing the normalised expression of different haemoglobin paralogs across ecotypes and lakes. (D) Expression of two genes (ABCC8 and ALDOA) that are significantly differentially expressed in 5 out of 7 ecotype pair comparisons. The boxplots show the normalized expression for each ecotype by lake (lakes highlighted by alternating shaded areas) and ecotypes are colour coded. (E) Distribution of explained variances for each transcript by model term for the linear mixed-effects model of gene expression. (F) Correlation between expression of WGCNA module eigengenes and lake (population of origin) and ecotype. Each row represents a module of co-expressed genes (identified by colour). Spearman’s correlation coefficients for the module expression-variable correlations are given in each cell. The corresponding corrected p-values are given in parenthesis. Significant correlations are highlighted in bold. Cells are coloured based on their correlation, with orange cells being correlated with up-regulation of gene expression in benthivorous ecotypes and blue being associated with up-regulation in planktivorous ecotypes. (G) Distribution of gene expression trajectory angles and differences in trajectory lengths for within and between ecotype comparisons. Distributions are coloured by comparisons between replicated ecotype-pairs (red; N = 6) and between non-replicated ecotype pairs (blue; N = 9). The mean for each dataset is shown by the solid lines. Means do not differ [file pgen.1008658.s015.tiff]

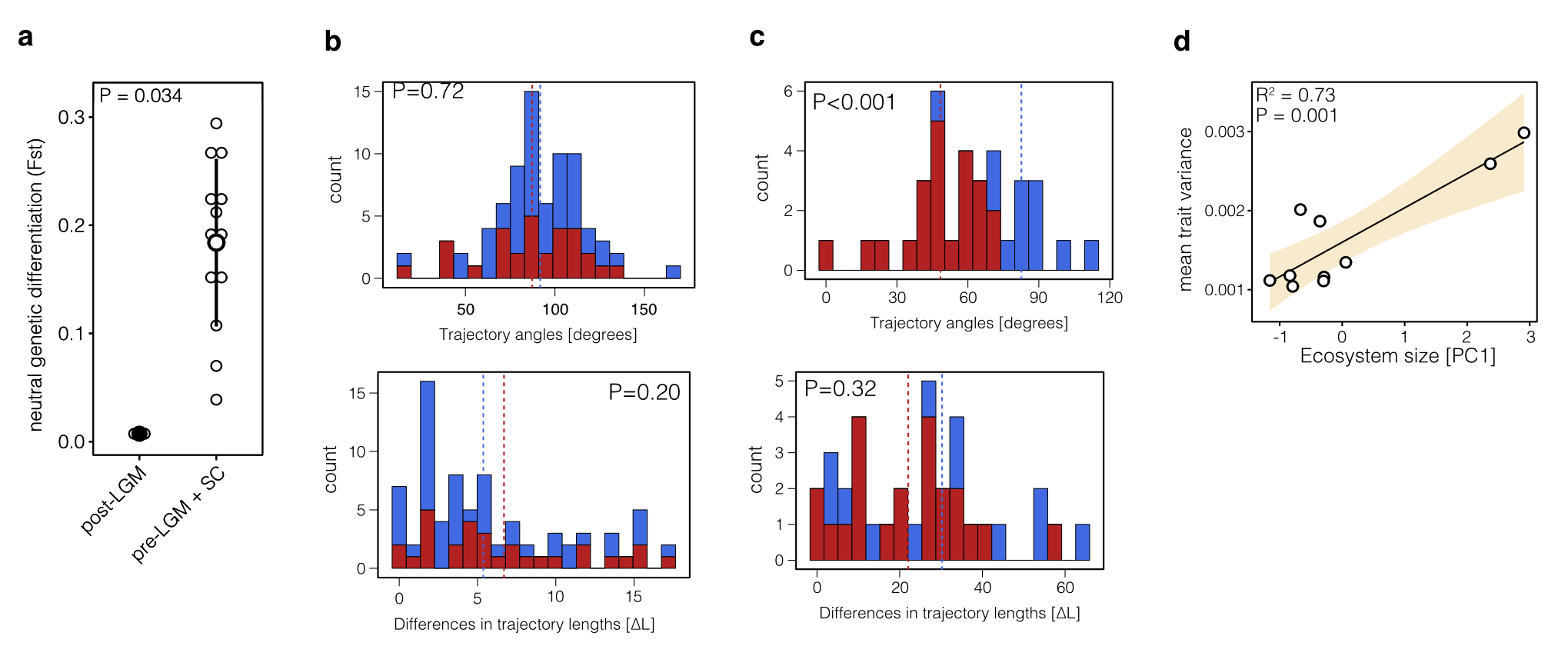

Supplement: S15 Fig — (A) Comparison of neutral genetic differentiation between sympatric ecotypes diverged post-LGM under ongoing gene flow or pre-LGM with secondary contact. P-value for Wilcoxon-test is shown in the plot. (B,C) Distribution of (B) neutral allele frequency trajectory angles and differences in trajectory lengths and (C) adaptive allele frequency trajectory angles and lengths for within and between ecotype comparisons. Comparisons are coloured by comparisons between replicated ecotype-pairs (red) and between non-replicated ecotype pairs (blue). The mean for each dataset is shown by the solid lines and the P-value for Wilcoxon-tests is shown in the plot. (D) Correlation between mean trait variance across all sympatric ecotypes and ecosystem size (PC1) across all populations. Larger ecosystems harbor populations with a larger mean trait variance (results of linear model shown in plot). (TIFF) [file pgen.1008658.s016.tiff]
