# Supplementary material for: Parallelism in eco-morphology and gene expression despite variable evolutionary and genomic backgrounds in a Holarctic fish
Source: PLoS Genet. 2020 Apr 17;16(4):e1008658. doi: 10.1371/journal.pgen.1008658 (PMC7164584; doi:10.1371/journal.pgen.1008658)
Supplement: S1 Table — (DOCX) [file pgen.1008658.s017.docx]

**Table S1**. Sampling sites and sample sizes for populations used for phenotypic and genomic analysis of Arctic charr from the Atlantic and Siberian lineage.

**Atlantic**

| Lake | Abb^1^ | Catchment | Lat. (N) | Lon. (W) | N phenotyped individuals | | | N genotyped individuals | | |
| --- | --- | --- | --- | --- | --- | --- | --- | --- | --- | --- |
|  |  |  |  |  | Bn | Pl | Uni | Bn | Pl | Uni |
| Tay^3^ | Tay | Tay | 56˚30’ | 004˚10’ | 43 | 76 | - | 21 | 31 | - |
| Rannoch | Ran | Tay | 56˚41’ | 004˚16’ | - | - | - | - | - | 11 |
| Awe^3^ | Awe | Awe | 56˚20’ | 005˚05’ | 34 | 36 | - | 25 | 28 | - |
| Eck | Eck | Cowal | 56˚01’ | 004˚58’ | - | - | - | - | - | 19 |
| Lubnaig | Lub | Forth | 56˚17’ | 004˚17’ | - | - | - | - | - | 20 |
| Dughaill^3^ | Dug | Ewe | 57˚28’ | 005˚20’ | 44 | 52 | - | 31 | 31 | - |
| Uaine | Uai | Ewe | 57˚31’ | 005˚23’ | - | - | - | - | - | 23 |
| naSealga | naS | - | 57˚79’ | 004˚30’ | 41 | 19 | - | 19 | 21 | - |
| Merkland | Mer | Shin | 58˚14’ | 004˚44’ | - | - | - | - | - | 22 |

**Siberian**

| Lake | Abb^1^ | Catchment | Lat. (N) | Lon. (E) | N phenotyped individuals | | | | N genotyped individuals | | | |
| --- | --- | --- | --- | --- | --- | --- | --- | --- | --- | --- | --- | --- |
|  |  |  |  |  | Bn | Pl | Pisc | Insct | Bn | Pl | Pisc | Insct |
| Kudushkit | Kud | Chaya | 56˚33’ | 110˚37’ | - | - | 20 | - | - | 20 | 19 | - |
| Davatchan | Dav | Chara, Olekma | 56˚27’ | 117˚33’ | 76 | 114 | - | - | 20 | 25 | - | - |
| Kalarskii Davatchan | KDa | Kalar,Vitim | 56˚16.5’ | 116˚38.5’ | - | 83 | 15/  81^2^ | - | - | 19 | 12/  21^2^ | - |
| Kiryalta-3 | Kir3 | Chara, Olekma | 57˚08.5’ | 119˚27’ | - | 23 | 30 | - | - | 20 | 19 | - |
| Kiryalta-4 | Kir4 | Chara, Olekma | 57˚06.5’ | 119˚28’ | - | 42 | 25 | - | - | 21 | 13 | - |
| Tokko^3^ | Tok | Tokko, Chara, Olekma | 57˚11’ | 119˚41’ | 42 | - | - | 47 | 18 | - | - | 19 |
| Kamkanda^3^ | Kam | Khani, Olekma | 57˚05.5’ | 199˚48.5’ | 146 | 163 | 26 | - | 20 | 19 | 20 | - |
| Bol’shoe Leprindo | BLe | Chara, Olekma | 56˚37’ | 117˚31’ | - | 25 | - | - | - | 10 | - | - |
| Maloe Leprindo | MLe | Chara, Olekma | 56˚36’ | 117˚22’ | - | 26 | - | - | - | 11 | - | - |

Note: ^1^Abbrevations used for these populations in the manuscript. ^2^ There are two piscivorous ecotypes in Kalarskii Davatchan, large piscivorous / small piscivorous ecotype (Fig. 1d). Ecotype abbreviations: Bn – benthivorous, PL – planktivorous, Uni – unimodal-planktivorous populations or without phenotypic information (Rannoch), Pisc – piscivorous, Insct – insectivorous. ^3^Subset of lakes for which RNAseq data were available for four individuals per ecotype.
