# Supplementary material for: Parallelism in eco-morphology and gene expression despite variable evolutionary and genomic backgrounds in a Holarctic fish
Source: PLoS Genet. 2020 Apr 17;16(4):e1008658. doi: 10.1371/journal.pgen.1008658 (PMC7164584; doi:10.1371/journal.pgen.1008658)
Supplement: S2 Table — Differences in trajectory lengths (ΔL, upper part) or angles (θ, lower part) below the diagonal and p-values are above the diagonal. (DOCX) [file pgen.1008658.s018.docx]

**Table S2.** Results of phenotypic trajectory analysis based on all seven linear traits. Differences in trajectory lengths (∆L, upper part) or angles (**𝛳**, lower part) below the diagonal and p-values are above the diagonal. Parallel (i.e. p > 0.05) ecotype pairs are highlighted in bold.

| **∆L/p-value** | Awe_bn-pl | Dav_bn-pl | Dug_bn-pl | Kam_bn-pl | Kam_pl-pisc | KDa_pl-pisc-s | KDa_pl-pisc | Kir3_pisc-pl | Kir4_pisc-pl | naS_bn-pl | Tay_bn-pl |
| --- | --- | --- | --- | --- | --- | --- | --- | --- | --- | --- | --- |
| Awe_bn-pl | 0 | 0.001 | 0.04 | 0.001 | **0.078** | **0.683** | 0.001 | 0.002 | 0.04 | **0.584** | **0.254** |
| Dav_bn-pl | 0.0699 | 0 | 0.013 | 0.049 | 0.01 | 0.001 | **0.512** | **0.979** | **0.061** | 0.001 | 0.001 |
| Dug_bn-pl | 0.0347 | 0.0352 | 0 | **0.244** | **0.824** | 0.037 | **0.185** | **0.056** | **0.811** | 0.017 | **0.336** |
| Kam_bn-pl | 0.0499 | 0.0200 | **0.0152** | 0 | **0.143** | 0.001 | **0.507** | **0.195** | **0.485** | 0.003 | 0.02 |
| Kam_pl-pisc | **0.0311** | 0.0388 | **0.0036** | **0.0188** | 0 | **0.083** | **0.123** | 0.044 | **0.625** | 0.033 | **0.49** |
| KDa_pl-pisc-s | **0.0061** | 0.0638 | 0.0285 | 0.0437 | **0.0249** | 0 | 0.003 | 0.001 | 0.036 | **0.291** | **0.322** |
| KDa_pl-pisc | 0.0594 | **0.0105** | **0.0247** | **0.0095** | **0.0283** | 0.0532 | 0 | **0.598** | **0.311** | 0.002 | 0.023 |
| Kir3_pisc-pl | 0.0703 | **0.0004** | **0.0356** | **0.0204** | 0.0392 | 0.0641 | **0.0109** | 0 | **0.139** | 0.001 | 0.009 |
| Kir4_pisc-pl | 0.0395 | **0.0304** | **0.0049** | **0.0103** | **0.0085** | 0.0334 | **0.0198** | **0.0307** | 0 | 0.015 | **0.255** |
| naS_bn-pl | **0.0110** | 0.0809 | 0.0456 | 0.0608 | 0.0420 | **0.0171** | 0.0703 | 0.0812 | 0.0505 | 0 | **0.096** |
| Tay_bn-pl | **0.0197** | 0.0502 | **0.0150** | 0.0302 | **0.0114** | **0.0135** | 0.0397 | 0.0506 | **0.0199** | **0.0307** | 0 |
| **𝛳/p-value** |  |  |  |  |  |  |  |  |  |  |  |
| Awe_bn-pl | 0 | 0.001 | 0.001 | 0.001 | 0.001 | 0.001 | 0.001 | 0.001 | 0.001 | 0.001 | 0.005 |
| Dav_bn-pl | 73.04 | 0 | 0.02 | **0.271** | 0.001 | 0.001 | 0.001 | 0.001 | 0.001 | 0.001 | 0.001 |
| Dug_bn-pl | 100.05 | 32.57 | 0 | **0.066** | 0.001 | 0.001 | 0.002 | 0.001 | 0.001 | 0.003 | 0.001 |
| Kam_bn-pl | 85.81 | **14.20** | **24.40** | 0 | 0.001 | 0.001 | 0.002 | 0.001 | 0.001 | 0.001 | 0.001 |
| Kam_pl-pisc | 87.22 | 120.36 | 129.13 | 119.15 | 0 | 0.001 | 0.001 | **0.742** | 0.007 | 0.001 | 0.001 |
| KDa_pl-pisc-s | 109.75 | 67.19 | 67.91 | 57.54 | 72.49 | 0 | **0.503** | 0.003 | **0.05** | 0.001 | 0.001 |
| KDa_pl-pisc | 115.10 | 74.48 | 68.77 | 63.23 | 69.84 | **17.87** | 0 | 0.022 | **0.35** | 0.001 | 0.001 |
| Kir3_pisc-pl | 94.41 | 111.25 | 115.13 | 107.00 | **16.46** | 58.83 | 54.08 | 0 | **0.126** | 0.001 | 0.001 |
| Kir4_pisc-pl | 98.00 | 78.35 | 78.59 | 71.50 | 52.45 | **33.51** | **26.81** | **37.22** | 0 | 0.001 | 0.001 |
| naS_bn-pl | 133.48 | 104.88 | 86.79 | 97.24 | 101.70 | 92.05 | 88.51 | 101.18 | 103.43 | 0 | 0.001 |
| Tay_bn-pl | 48.08 | 45.61 | 71.44 | 57.55 | 102.15 | 86.44 | 99.48 | 104.24 | 91.10 | 121.13 | 0 |
