# Supplementary material for: Parallelism in eco-morphology and gene expression despite variable evolutionary and genomic backgrounds in a Holarctic fish
Source: PLoS Genet. 2020 Apr 17;16(4):e1008658. doi: 10.1371/journal.pgen.1008658 (PMC7164584; doi:10.1371/journal.pgen.1008658)
Supplement: S3 Table — (DOCX) [file pgen.1008658.s019.docx]

**Table S3**. Effect sizes (partial η^2^) for each model term from trait-by-trait linear models.

| Trait/PC | Ecotype (η^2^) | Ecotype x Lake (η^2^) | Lake (η^2^) | Lineage (η^2^) | Ecotype x Lineage (η^2^) |
| --- | --- | --- | --- | --- | --- |
| phen.PC1 | 0.3012 | 0.0338 | 0.355 | 0.1873 | 0.0452 |
| phen.PC2 | 0.4499 | 0.031 | 0.2772 | 0.7783 | 0.0046 |
| phen.PC3 | 0.405 | 0.0329 | 0.172 | 0.211 | 0.0000 |
| phen.PC4 | 0.0732 | 0.1039 | 0.1874 | 0.0001 | 0.0001 |
| HDO | 0.5666 | 0.0857 | 0.332 | 0.9235 | 0.1048 |
| HDE | 0.1616 | 0.0624 | 0.3327 | 0.4066 | 0.0152 |
| HL | 0.0701 | 0.0102 | 0.2108 | 0.018 | 0.0166 |
| PFL | 0.4232 | 0.0356 | 0.2024 | 0.2041 | 0.0094 |
| ED | 0.3083 | 0.0360 | 0.1923 | 0.0025 | 0.0144 |
| ML | 0.1051 | 0.0114 | 0.287 | 0.1143 | 0.0146 |
| LJL | 0.0932 | 0.0159 | 0.1758 | 0.0083 | 0.0086 |

Note: phen.PC – PC1 to PC4 from principal components analysis performed on all seven linear traits. HDO – head depth at operculum, HDE – head depth at eye, HL – head length, PFL – pectoral fin length, ED – eye diameter, ML – maxilla length, LJL – lower jaw length.
