# Supplementary material for: Parallelism in eco-morphology and gene expression despite variable evolutionary and genomic backgrounds in a Holarctic fish
Source: PLoS Genet. 2020 Apr 17;16(4):e1008658. doi: 10.1371/journal.pgen.1008658 (PMC7164584; doi:10.1371/journal.pgen.1008658)
Supplement: S4 Table — (DOCX) [file pgen.1008658.s020.docx]

**Table S4.** Results of the *f4*- and *f3*-statistics for both lineages.

| *f4*-statistics | | | | | | | | |
| --- | --- | --- | --- | --- | --- | --- | --- | --- |
| PopA | **PopB** | **PopC** | **PopD** | ***f4*** | | **se** | | **Z-score** |
| Dav-bn | BLe | KDa-pisc | KDa-pisc-s | -0.00267318 | | 0.00055094 | | -4.85202 |
| Dav-bn | BLe | Kud-pisc | Kud-pl | 0.00172862 | | 0.000546439 | | 3.16342 |
| Dav-bn | Dav-pl | KDa-pisc | KDa-pl | -0.00281651 | | 0.00142346 | | -1.97865 |
| Kam-pl | Kam-bn | KDa-pisc | KDa-pisc-s | -0.00056964 | | 0.000272173 | | -2.09294 |
| Kam-pl | Kam-pisc | KDa-pisc | KDa-pisc-s | -0.00135753 | | 0.000374307 | | -3.62679 |
| KDa-pisc | KDa-pisc-s | Tok-bn | Tok-insct | -0.000578487 | | 0.000183728 | | -3.14862 |
| KDa-pisc | KDa-pisc-s | MLe | BLe | -0.000742263 | | 0.000269313 | | -2.75613 |
| Kir4-pisc | Kir4-pl | Kud-pisc | Kud-pl | -0.000837066 | | 0.000326568 | | -2.56322 |
| Kir4-pisc | Kir4-pl | KDa-pisc | KDa-pisc-s | 0.000539178 | | 0.00026962 | | 1.99977 |
| Kir4-pisc | Kir4-pl | Kir3-pisc | Kir3-pl | 0.0229266 | | 0.001653 | | 13.8697 |
| Kud-pisc | Kud-pl | KDa-pisc | KDa-pisc-s | -0.00175827 | | 0.000372013 | | -4.72636 |
| Kud-pisc | Kud-pl | KDa-pisc | KDa-pl | -0.00174521 | | 0.000525462 | | -3.32129 |
| Kud-pisc | Kud-pl | MLe | BLe | 0.000717697 | | 0.000270048 | | 2.65766 |
| Dug-bn | Eck | Dug-pl | Uai | 0.0216789 | | 0.00161344 | | 13.4364 |
| Dug-bn | Lub | Dug-pl | Uai | 0.0216151 | | 0.00153642 | | 14.0685 |
| Tay-pl | Tay-bn | Awe-pl | Dug-pl | -0.00484982 | | 0.00211385 | | -2.29431 |
| Tay-pl | Tay-bn | Awe-pl | Eck | -0.00888889 | | 0.00248355 | | -3.5791 |
| Tay-pl | Tay-bn | Awe-pl | Mer | -0.00576767 | | 0.00231408 | | -2.49242 |
| Tay-pl | Tay-bn | Awe-pl | naSeal-pl | -0.00527456 | | 0.00239611 | | -2.2013 |
| Tay-pl | Tay-bn | Awe-pl | Uai | -0.00525318 | | 0.00238727 | | -2.2005 |
| Tay-pl | Tay-bn | Awe-pl | Awe-bn | -0.000440399 | | 0.00040726 | | -1.0814 |
| Dug-bn | Dug-pl | Awe-pl | Awe-bn | -0.00018956 | | 0.000398061 | | -0.47621 |
| Dug-bn | Dug-pl | naSeal-bn | naSeal-pl | -0.00014003 | | 0.000363499 | | -0.38522 |
| Dug-bn | Dug-pl | Tay-pl | Tay-bn | 0.00029 | | 0.001732 | | 0.167554 |
| Dav-bn | Dav-pl | Kam-pl | Kam-bn | -0.00059 | | 0.00101958 | | -0.578558 |
| Kam-pl | Kam-pisc | KDa-pisc | KDa-pl | -0.00128912 | | 0.0012837 | | -1.00422 |
| Kam-pl | Kam-pisc | KDa-pisc-s | KDa-pl | 0.0000684 | | 0.00121126 | | 0.056479 |
| Kam-pl | Kam-pisc | Kir3-pisc | Kir3-pl | 6.46E-06 | | 0.0011322 | | 0.0057079 |
| Kam-pl | Kam-pisc | Kir4-pisc | Kir4-pl | 0.00034444 | | 0.0008770 | | 0.392734 |
| Kam-pl | Kam-pisc | Kud-pisc | Kud-pl | 0.0003731 | | 0.00032198 | | 1.15871 |
| Kir3-pisc | Kir3-pl | KDa-pisc | KDa-pl | 0.00048186 | | 0.00109459 | | 0.440217 |
| Kir3-pisc | Kir3-pl | Kud-pisc | Kud-pl | 0.00002218 | | 0.000334743 | | 0.066266 |
| Kir4-pisc | Kir4-pl | KDa-pisc | KDa-pl | 0.0006093 | | 0.00100214 | | 0.608 |
| Kir4-pisc | Kir4-pl | KDa-pisc-s | KDa-pl | 7.01E-05 | | 0.000954315 | | 0.07348 |
| Kud-pisc | Kud-pl | KDa-pisc-s | KDa-pl | 1.31E-05 | | 0.000354074 | | 0.0368675 |
| *f3*-statistics | | | | | | |  |  |
| Focal | **Ref1** | **Ref2** | ***f3*** | **se** | **Z-score** | |  |  |
| Dug-pl | Dug-bn | Uai | -0.0259701 | 0.00110819 | -23.4348 | |  |  |
| Dug-pl | Tay-bn | Uai | -0.00475052 | 0.00127475 | -3.72663 | |  |  |
| Dug-pl | Ran | Uai | -0.00448714 | 0.00123844 | -3.62322 | |  |  |
| Dug-pl | Tay-pl | Uai | -0.00434716 | 0.00126704 | -3.43097 | |  |  |
| Dug-pl | Uai | Lub | -0.00435496 | 0.00140769 | -3.0937 | |  |  |
| Dug-pl | Awe-pl | Uai | -0.0037364 | 0.00131955 | -2.83157 | |  |  |
| Dug-pl | Eck | Uai | -0.0042912 | 0.00153863 | -2.78897 | |  |  |
| Dug-pl | Uai | Awe-bn | -0.00371748 | 0.00133324 | -2.7883 | |  |  |
| Dug-pl | Uai | naSeal-pl | -0.00329079 | 0.00140305 | -2.34546 | |  |  |
| Dug-pl | naSeal-bn | Uai | -0.00295874 | 0.00140562 | -2.10494 | |  |  |
| Dug-pl | Uai | Mer | -0.0030708 | 0.00146056 | -2.10249 | |  |  |
| KDa-pisc-s | KDa-pisc | KDa-pl | -0.00249944 | 0.000420217 | -5.94796 | |  |  |
| KDa-pisc-s | Kud-pisc | KDa-pl | -0.00191366 | 0.000606432 | -3.15561 | |  |  |
| KDa-pisc-s | KDa-pisc | Tok-bn | -0.00136393 | 0.000597315 | -2.28343 | |  |  |

Note: se – standard error; Focal – Focal population in which admixture is detected; Ref – Reference populations 1 and 2.
