# Supplementary material for: Parallelism in eco-morphology and gene expression despite variable evolutionary and genomic backgrounds in a Holarctic fish
Source: PLoS Genet. 2020 Apr 17;16(4):e1008658. doi: 10.1371/journal.pgen.1008658 (PMC7164584; doi:10.1371/journal.pgen.1008658)
Supplement: S6 Table — (DOCX) [file pgen.1008658.s022.docx]

**Table S6.** Results of Fst genome scans and associated analyses between sympatric ecotypes.

| Lake | Eco | mean Fst | outlier Fst | 95t^h^ perc. | N. outlier | N.ZFst>2 | N.ZFst>3 | N.ZFst>4 | N. fixed SNPs | % fixed | Pst (mean) |
| --- | --- | --- | --- | --- | --- | --- | --- | --- | --- | --- | --- |
| Awe | Bn-Pl | 0.0113 | 0.1012 | 0.0653 | 175 | 185 | 86 | 40 | 0 | 0.00 | 0.448 |
| Tay | Bn-Pl | 0.2262 | 0.8483 | 0.7391 | 209 | 273 | 36 | 0 | 4 | 0.10 | 0.525 |
| Dughaill | Bn-Pl | 0.2524 | 0.7537 | 0.6814 | 178 | 142 | 0 | 0 | 0 | 0.00 | 0.852 |
| naSealga | Bn-Pl | 0.0136 | 0.1195 | 0.0795 | 131 | 146 | 60 | 27 | 0 | 0.00 | 0.509 |
| Davatchan | Bn-Pl | 0.3291 | 0.9898 | 0.9523 | 113 | 127 | 0 | 0 | 76 | 3.38 | 0.982 |
| Kamkanda | Bn-Pl | 0.1336 | 0.6300 | 0.4935 | 96 | 99 | 36 | 4 | 0 | 0.00 | 0.988 |
| Kamkanda | Pl-Pisc | 0.2445 | 0.8567 | 0.7592 | 90 | 106 | 1 | 0 | 1 | 0.06 | 0.836 |
| Kalarskii Dv | Pl-Pisc | 0.2976 | 0.9569 | 0.8748 | 85 | 99 | 0 | 0 | 35 | 2.08 | 0.878 |
| Kalarskii Dv | Pl-Pisc-s | 0.2587 | 0.9393 | 0.8549 | 83 | 102 | 0 | 0 | 18 | 1.08 | 0.761 |
| Kiryalta-3 | Pl-Pisc | 0.3074 | 0.9530 | 0.8845 | 59 | 51 | 0 | 0 | 15 | 1.26 | 0.853 |
| Kiryalta-4 | Pl-Pisc | 0.1837 | 0.8208 | 0.6731 | 86 | 112 | 36 | 0 | 0 | 0.00 | 0.798 |
| Kudushkit | Pl-Pisc | 0.1003 | 0.6621 | 0.4677 | 37 | 40 | 19 | 10 | 1 | 0.14 | - |
| Tokko | Bn-Insct | 0.2233 | 0.8593 | 0.7557 | 46 | 54 | 2 | 0 | 2 | 0.22 | 0.974 |

Note: Bn – benthivorous, Pl – planktivorous, Pisc – piscivorous, Pisc-s – small-piscicorous, Insct – insectivorous. 95t^h^ perc. – 95^th^ percentile of Fst distribution used for identifying outliers. N.outlier – Number of outlier loci identified. N.ZFst – number of SNPs with a z-transformed Fst above the threshold (2, 3 or 4). N.fixed SNPs – Number of fixed SNPs between ecotypes. % fixed – Percentage of fixed SNPs compared to all SNPs in the comparison. Pst (mean) – mean phenotypic divergence (Pst) across traits between sympatric ecotypes.
