# Supplementary material for: Parallelism in eco-morphology and gene expression despite variable evolutionary and genomic backgrounds in a Holarctic fish
Source: PLoS Genet. 2020 Apr 17;16(4):e1008658. doi: 10.1371/journal.pgen.1008658 (PMC7164584; doi:10.1371/journal.pgen.1008658)
Supplement: S7 Table — Shown are the four best fitting models for each population/comparison and the respective ΔAIC between them. (DOCX) [file pgen.1008658.s023.docx]

**Table S7.** The most likely demographic models for each lake population. Shown are the fours best fitting models for each population/comparison and the respective ∆AIC between them.

| Lake/Ecotypes | N pop. | N SNPs | Best model | 2^nd^ best (∆AIC) | 3^rd^ best (∆AIC) | 4^th^ best (∆AIC) |
| --- | --- | --- | --- | --- | --- | --- |
| Awe | 2pop | 15,891 | IMint | SCint (12.4) | IMchange (25.1) | SC (50.0) |
| Tay | 2pop | 15,000 | IMint | SCint (0.15) | SC (12.5) | IMchange (13.7) |
| naS | 2pop | 13,687 | IMint | SCint (4.8) | IMchange (21.8) | SC (104) |
| Dug-bn - Dug-pl | 2pop | 15,096 | IMint | IMchange (3.8) | SCint (68.8) | SC (69.1) |
| Dug-bn - Uai | 2pop | 11,372 | IMchange | IMint (10.3) | SCint (95.5) | SC (137) |
| Dug-pl - Uai | 2pop | 7,231 | IMint | IMchange (0.65) | SCint (1.9) | SC (4.1) |
| Dug | 3pop | 11,753 | IMint | HybMig (89.3) | IM (129) | Int (142) |
| Dav | 2pop | 6,931 | SCint | IMint (3.1) | IMchange (5.1) | SC (26.4) |
| Kir-3 | 2pop | 6.493 | SC | Imchange (3.8) | SCint (4.4) | IMint (6.7) |
| Kir-4 | 2pop | 5,641 | SCint | SC (0.65) | IMint (3.5) | IMchange (3.7) |
| Tok | 2pop | 4,285 | SC | SCint (2.9) | IMchange (3.3) | IMint (5.9) |
| Kam-PiscPl | 2pop | 6,686 | SC | SCint (0.4) | IMchange (2.9) | IMint (4.0) |
| Kam-BnPl | 2pop | 7,068 | SC | SCint (2.3) | IMchange (5.5) | IMint (7.8) |
| Kam-BnPisc | 2pop | 6,941 | SC | SCint (0.14) | IMint (4.1) | IMchange (4.9) |
| KDa-PiscPl | 2pop | 6,528 | IMint | SC (4.2) | SCint (4.5) | IMchange (9.7) |
| KDa-PiscSPl | 2pop | 6,718 | SC | SCint (2.7) | IMchange (3.9) | IMint (8.3) |
| KDa-PiscPiscS | 2pop | 6,700 | SC | IMchange (1.5) | SCint (2.1) | IMint (7.1) |
| Kud | 2pop | 6,194 | SCint | IMint (4.5) | IMchange (8.2) | SC (18.1) |
| Kam | 3pop | 6,634 | SC | IM (0.33) | SCint (4.3) | IMchange (4.6) |
| KDa | 3pop | 6,310 | SC | IntSC (5.27) | SCint (8.4) | Hyb (12.9) |

Note: 2pop models tested the demographic history for ecotype pairs and 3pop models modelled the demographic history for three populations/ecotypes. N SNPs gives the number of SNPs used to build the folded site frequency spectrum.
