# Supplementary material for: Parallelism in eco-morphology and gene expression despite variable evolutionary and genomic backgrounds in a Holarctic fish
Source: PLoS Genet. 2020 Apr 17;16(4):e1008658. doi: 10.1371/journal.pgen.1008658 (PMC7164584; doi:10.1371/journal.pgen.1008658)
Supplement: S8 Table — (DOCX) [file pgen.1008658.s024.docx]

**Table S8.** Genes containing ecotype-associated SNPs identified in the RDA analysis in both lineages.

| Contig | SNP | Pos | Chrom + Pos | Gene upstream | Gene downstream | Z-score (ATL) RDA1 | Z-score (SIB) RDA1 | Z-score (SIB) RDA2 |
| --- | --- | --- | --- | --- | --- | --- | --- | --- |
| Contig1179 | 5257_34 | 2295862 | Chr. 7 (19321141 - 22679631) | PTPRJ |  | ﻿-2.55 | ﻿-3.02 | 0.22 |
| Contig1218 | 6352_25 | 1446318 | Chr. 14 (26623548 -29584114) | NKAIN2 |  | ﻿-2.07 | ﻿-2.33 | ﻿-0.14 |
| Contig1811 | 20016_37 | 380212 | Chr. 14 (17253565 - 17984788) | NRP1 |  | ﻿-2.07 | ﻿-2.50 | ﻿2.11 |
| Contig2079 | 23905_27 | 770770 | Chr. 10 (27535018 - 28863258) | ZNF536 | ZNF507-like | ﻿-2.05 | ﻿-2.39 | ﻿-0.63 |
| Contig4128 | 42487_17 | 1064984 | Chr. 32 (29201853 - 30813734) | frrs1 | CASQ1 | ﻿-2.48 | ﻿-2.51 | ﻿-1.38 |
| Contig4165 | 42701_25 | 784583 | Chr. 25 (14886004 - 16291508) | SER1 | PLEK | ﻿2.56 | ﻿-2.50 | ﻿-0.96 |
| Contig826 | 60249_16 | 1161922 | Chr. 20b (51294406 - 54822464) | uncharacterized |  | ﻿-2.24 | ﻿0.17 | ﻿-2.56 |
| Contig949 | 65145_41 | 239650 | Chr. 31 (22995782 -24834051) | CTDP1 |  | ﻿2.21 | ﻿-1.25 | ﻿-3.15 |

Note: Pos = Position; Chrom + Posi= Chromosome corresponding to a contig, and start and end position of the contig on the corresponding chromosome in bp; Z-score (ATL) RDA1 = z-transformed loading for candidate SNP along RDA1 in Atlantic lineage; Z-score (SIB) RDA1 = z-transformed loading for candidate SNP along RDA1 in the Siberian lineage, associated with benthic-pelagic divergence; Z-score (SIB) RDA2 = z-transformed loading for candidate SNP along RDA2 in the Siberian lineage, associated with piscivorous divergence
