# Supplementary material for: Parallelism in eco-morphology and gene expression despite variable evolutionary and genomic backgrounds in a Holarctic fish
Source: PLoS Genet. 2020 Apr 17;16(4):e1008658. doi: 10.1371/journal.pgen.1008658 (PMC7164584; doi:10.1371/journal.pgen.1008658)
Supplement: S9 Table — (DOCX) [file pgen.1008658.s025.docx]

**Table S9.** Gene ontology overrepresentation results for ecotype-associated expressed genes (RDA).

| **Term** | **Geneset** | **Description** | **Genes** | **Enriched** | **P-value** | **FDR** |
| --- | --- | --- | --- | --- | --- | --- |
| GO_process | GO:0007049 | cell cycle | 61 | 1.9019678 | 2.99E-07 | 7.30E-05 |
| GO_process | GO:0051276 | chromosome organization | 28 | 2.49927495 | 2.70E-06 | 3.29E-04 |
| GO_process | GO:0007017 | microtubule-based process | 36 | 1.85176304 | 0.00015586 | 0.01267662 |
| GO_process | GO:0006325 | chromatin organization | 33 | 1.73669684 | 0.00096548 | 0.05889419 |
| GO_process | GO:0051301 | cell division | 20 | 2.02322258 | 0.00146254 | 0.07137213 |
| GO_process | GO:0071824 | protein-DNA complex subunit organization | 11 | 2.38451233 | 0.00453561 | 0.18444807 |
| GO_process | GO:0007059 | chromosome segregation | 14 | 1.99160973 | 0.00835272 | 0.28611823 |
| GO_process | GO:0048285 | organelle fission | 23 | 1.66193283 | 0.00938093 | 0.28611823 |
| GO_process | GO:0033554 | cellular response to stress | 42 | 1.4215207 | 0.01085075 | 0.2941758 |
| GO_process | GO:0007010 | cytoskeleton organization | 41 | 1.40332544 | 0.0144847 | 0.34976538 |
| KEGG | dre04110 | Cell cycle - Danio rerio (zebrafish) | 25 | 1.89315199 | 0.00100108 | 0.15116286 |
| KEGG | dre03040 | Spliceosome - Danio rerio (zebrafish) | 23 | 1.85671774 | 0.00207611 | 0.15674653 |
| KEGG | dre00561 | Glycerolipid metabolism - Danio rerio (zebrafish) | 12 | 2.05369128 | 0.01058763 | 0.53291069 |
| KEGG | dre00340 | Histidine metabolism - Danio rerio (zebrafish) | 6 | 2.56711409 | 0.02278405 | 0.62554397 |
| KEGG | dre00040 | Pentose and glucuronate interconversions - Danio rerio (zebrafish) | 5 | 2.85234899 | 0.02361859 | 0.62554397 |
| KEGG | dre04540 | Gap junction - Danio rerio (zebrafish) | 12 | 1.80148357 | 0.0290231 | 0.62554397 |
| KEGG | dre04330 | Notch signaling pathway - Danio rerio (zebrafish) | 9 | 1.97470315 | 0.0322557 | 0.62554397 |
| KEGG | dre00240 | Pyrimidine metabolism - Danio rerio (zebrafish) | 16 | 1.61073826 | 0.03466125 | 0.62554397 |
| KEGG | dre00970 | Aminoacyl-tRNA biosynthesis - Danio rerio (zebrafish) | 8 | 2.01342282 | 0.0381549 | 0.62554397 |
| KEGG | dre00310 | Lysine degradation - Danio rerio (zebrafish) | 10 | 1.82064829 | 0.04142675 | 0.62554397 |

Note: Terms – GO_process = Gene ontology biological processes, KEGG = KEGG pathways; Gene set – GO term ID or KEGG pathway ID; Genes – Number of ecotype-associated genes in each pathway; Enriched – Fold enrichment; FDR – False discovery rate.
