# Supplementary material for: Parallelism in eco-morphology and gene expression despite variable evolutionary and genomic backgrounds in a Holarctic fish
Source: PLoS Genet. 2020 Apr 17;16(4):e1008658. doi: 10.1371/journal.pgen.1008658 (PMC7164584; doi:10.1371/journal.pgen.1008658)
Supplement: S10 Table — Gene co-expression modules in network generated from 1,512 ecotype associated genes. (DOCX) [file pgen.1008658.s026.docx]

**Table S10. WGCNA networks.** Gene co-expression modules in network generated from 1,512 ecotype associated genes.

| Module | | Number genes in module | | | Significantly (FDR < 0.05) associated with | Ecotype associated with |  |  |
| --- | --- | --- | --- | --- | --- | --- | --- | --- |
| Blue | | 221 | | | Ecotype | pl |  |  |
| Cyan | | 42 | | | Ecotype | pl |  |  |
| Magenta | | 73 | | | Lake + Ecotype | pl |  |  |
| MidnightBlue | | 31 | | | Ecotype | pl |  |  |
| Salmon | | 43 | | | lake + Ecotype | pl |  |  |
| Black | | 98 | | | Ecotype | pl |  |  |
| Green | | 159 | | | Ecotype | pl |  |  |
| GreenYellow | | 43 | | | Ecotype | bn |  |  |
| LightCyan | | 73 | | | Lake + Ecotype | bn |  |  |
| Turquoise | | 384 | | | Ecotype | bn |  |  |
| Brown | | 162 | | | Ecotype | bn |  |  |
| Pink | | 73 | | | Ecotype | bn |  |  |
| Red | | 110 | | | Ecotype | bn |  |  |
| Module | **Ecotype associated with** | | **KEGG** | **KEGG pathway description** | | | | **FDR** |
| Blue | pl | | dre00240 | Pyrimidine metabolism - *Danio rerio* (zebrafish) | | | | 0.04 |
| Magenta | pl | | dre04510 | Focal adhesion - Danio rerio (zebrafish) | | | | 0.04 |
| Magenta | pl | | dre04512 | ECM-receptor interaction - Danio rerio (zebrafish) | | | | 0.04 |
| Black | pl | | dre00561 | Glycerolipid metabolism - Danio rerio (zebrafish) | | | | 0.02 |
| Black | pl | | dre04350 | TGF-beta signalling pathway - Danio rerio (zebrafish) | | | | 0.02 |
| Turquoise | bn | | dre04070 | Phosphatidylinositol signalling system - Danio rerio (zebrafish) | | | | 0.01 |
| Turquoise | bn | | dre00562 | Inositol phosphate metabolism - Danio rerio (zebrafish) | | | | 0.03 |

Note: Traits (lake and ecotype) significantly correlated (FDR < 0.05) with each module are given, as well the direction of expression for ecotype: pl = up-regulated in planktivorous (over benthivorous) and bn = up-regulated in benthivorous (over planktivorous). KEGG – KEGG pathway ID, FDR – False discovery rate
