# Supplementary material for: Parallelism in eco-morphology and gene expression despite variable evolutionary and genomic backgrounds in a Holarctic fish
Source: PLoS Genet. 2020 Apr 17;16(4):e1008658. doi: 10.1371/journal.pgen.1008658 (PMC7164584; doi:10.1371/journal.pgen.1008658)
Supplement: S11 Table — Ecotype-associated expressed genes that are associated with cis-eQTL. (DOCX) [file pgen.1008658.s027.docx]

**Table S11.** Cis-regulated ecotype-associated gene expression. Ecotype-associated genes that are associated with *cis*-eQTL.

| **Chrom** | **Pos** | **N.cis-SNPs** | **Gene** | **F-statistic** | **FDR** | **beta** | **R^2^** | **RDA.axis** | **RDA.loading** |
| --- | --- | --- | --- | --- | --- | --- | --- | --- | --- |
| Contig2861 | 682439 | 1 | GENE37518_R0 | -4.3035659 | 0.07864162 | -1.1812016 | 0.39811713 | 1 | 0.082149302 |
| Contig9511 | 57765 | 9 | RNA101009_R0 | -6.9147358 | 0.00147824 | -0.669493 | 0.63067299 | 1 | -0.094215753 |
| Contig706 | 35988 | 1 | RNA124088_R1 | 4.62134923 | 0.05210489 | 1.43091656 | 0.43270307 | 1 | -0.064353881 |
| Contig2100 | 384673 | 2 | RNA13698_R0 | -4.4057006 | 0.07225572 | -0.7968371 | 0.40940977 | 2 | -0.082842073 |
| Contig12095 | 1207 | 1 | RNA14706_R0 | -4.4032784 | 0.07225572 | -0.8662297 | 0.40914385 | 2 | -0.079057491 |
| Contig587 | 1174255 | 1 | RNA17901_R0 | -4.2087891 | 0.09003047 | -0.4451682 | 0.38749491 | 2 | 0.070797588 |
| Contig1414 | 2885789 | 1 | RNA18530_R0 | 4.97030319 | 0.03375758 | 2.21531134 | 0.46873016 | 1 | 0.072009723 |
| Contig1201 | 885031 | 2 | RNA21425_R0 | -4.1555588 | 0.09419291 | -0.4453406 | 0.38147066 | 2 | 0.071708183 |
| Contig904 | 1175920 | 1 | RNA21720_R0 | -4.8734871 | 0.04020891 | -0.4691819 | 0.45894637 | 1 | 0.107174449 |
| Contig1631 | 646839 | 2 | RNA23287_R1 | 4.2395809 | 0.0875681 | 0.26035486 | 0.39096072 | 2 | 0.090023064 |
| Contig2947 | 170266 | 2 | RNA25489_R0 | -4.566751 | 0.05634307 | -0.7769089 | 0.42687797 | 1 | 0.075613456 |
| Contig1397 | 391240 | 1 | RNA26428_R1 | -4.3621683 | 0.07529024 | -0.4727803 | 0.40461651 | 1 | -0.082573446 |
| Contig1358 | 911738 | 1 | RNA27893_R0 | -4.2044756 | 0.0902104 | -0.4292859 | 0.38700828 | 1 | -0.106208673 |
| Contig699 | 787330 | 3 | RNA35235_R1 | 4.54674664 | 0.05672388 | 0.55518712 | 0.42473128 | 1 | -0.094163469 |
| Contig3135 | 697831 | 1 | RNA46270_R0 | 4.65985404 | 0.05092883 | 0.53114402 | 0.43678109 | 2 | -0.058797069 |
| Contig1700 | 1584465 | 1 | RNA54079_R0 | 4.71775654 | 0.0464547 | 1.05908525 | 0.4428662 | 1 | -0.082845396 |
| Contig3633 | 211497 | 1 | RNA62277_R0 | 5.72605989 | 0.01237167 | 1.39145353 | 0.53938097 | 2 | -0.069869143 |
| Contig4257 | 38394 | 1 | RNA65452_R1 | -5.5394866 | 0.01759705 | -3.0711062 | 0.52288379 | 2 | -0.062621388 |
| Contig603 | 1161004 | 1 | RNA71628_R0 | 4.38937387 | 0.0724093 | 0.35069496 | 0.40761556 | 1 | 0.072100135 |

Note: Chrom – Contig, Pos – SNP position, N.cis-SNPs – Number of cis-QTL associated with expression of the particular gene. FDR – false discovery rate, beta – effect size of cis-eQTL. R^2^ – Effect size of the correlation between expression and genotype. RDA.axis – Axis of gene expression divergence from the RDA analysis (corresponds to Fig. 6d), RDA.loading – Loading of each gene along the corresponding RDA axis.

**Continued Table S11.**

| **Chrom** | **Pos** | **N.cis-SNPs** | **Gene** | **F-statistic** | **FDR** | **beta** | **R^2^** | **RDA.axis** | **RDA.loading** |
| --- | --- | --- | --- | --- | --- | --- | --- | --- | --- |
| Contig1708 | 223269 | 1 | RNA81939_R0 | 4.49981188 | 0.06100233 | 0.36702121 | 0.41966876 | 1 | -0.072706216 |
| Contig698 | 1344459 | 1 | RNA83442_R0 | -4.7282695 | 0.0464547 | -0.6191383 | 0.44396489 | 2 | 0.063960238 |
| Contig1005 | 1644002 | 1 | RNA85418_R0 | 4.44869915 | 0.06718682 | 0.44987576 | 0.4141147 | 2 | 0.086576251 |
| Contig985 | 1861483 | 1 | RNA86913_R1 | -5.0103838 | 0.032617 | -1.5054908 | 0.47273221 | 1 | 0.088755726 |
| Contig1560 | 1213339 | 1 | RNA8803_R1 | -4.6744939 | 0.05026973 | -0.8396593 | 0.43832501 | 2 | -0.075225058 |
| Contig836 | 348293 | 1 | RNA97564_R0 | 4.39942336 | 0.0724093 | 0.6248348 | 0.40872044 | 2 | -0.139056588 |

Note: Chrom. – Contig, Pos – SNP position, N.cis-SNPs – Number of cis-QTL associated with expression of the particular gene. FDR – false discovery rate, beta – effect size of cis-eQTL. R^2^ – Effect size of the correlation between expression and genotype. RDA.axis – Axis of gene expression divergence from the RDA analysis (corresponds to Fig. 6d), RDA.loading – Loading of each gene along the corresponding RDA axis.
